# Ferroelectric-assisted charge-transfer plasmons in graphene/CuInP_2_S_6_

**DOI:** 10.1038/s41467-026-76909-2

**Published:** 2026-08-17

**Authors:** Yinming Shao, Hae Yeon Lee, Jin Zhang, S. L. Moore, Shizhe Zhou, Carlton Drew, Anjaly Rajendran, Jacob Amontree, Thomas P. Darlington, Daniel G. Chica, Chun-Ying Huang, Seng Huat Lee, Saugata Sarker, Kenji Watanabe, Takashi Taniguchi, Venkatraman Gopalan, Zhiqiang Mao, X.-Y. Zhu, Xavier Roy, P. James Schuck, Angel Rubio, James C. Hone, D. N. Basov

**Affiliations:** 1 Department of Physics, Columbia University, New York, NY USA; 2 Department of Physics, The Pennsylvania State University, University Park, PA USA; 3 Materials Research Institute, The Pennsylvania State University, University Park, PA USA; 4 Department of Mechanical Engineering, Columbia University, New York, NY USA; 5 Department of Materials Science and NanoEngineering, Rice University, Houston, TX USA; 6 Max Planck Institute for Structure and Dynamics of Matter and Center for Free-Electron Laser Science, Hamburg, Germany; 7 Two-Dimensional Crystal Consortium, The Pennsylvania State University, University Park, PA USA; 8 Department of Electrical Engineering, Columbia University, New York, NY USA; 9 Department of Chemistry, Columbia University, New York, NY USA; 10 Department of Materials Science and Engineering, The Pennsylvania State University, University Park, PA USA; 11 Research Center for Electronic and Optical Materials, National Institute for Materials Science, Tsukuba, Japan; 12 Research Center for Materials Nanoarchitectonics, National Institute for Materials Science, Tsukuba, Japan; 13 Nano-Bio Spectroscopy Group, Universidad del País Vasco (UPV/EHU), San Sebastián, Spain; 14 The Initiative for Computational Catalysis (ICC) and the Center for Computational Quantum Physics (CCQ), Flatiron Institute, New York, NY USA

**Keywords:** Optical properties and devices, Surfaces, interfaces and thin films

## Abstract

Two-dimensional materials and their van der Waals heterostructures provide a versatile platform for novel optoelectronic functionalities. Interfacial charge transfer in such stacks enables high-quality graphene plasmons, but the charge-doping is fixed by work-function differences between constituent layers. Ferroelectric substrates have been explored for non-volatile, switchable doping, yet oxide ferroelectrics yield only modest doping due to imperfect interfaces. Here, we report efficient charge transfer between graphene and the van der Waals ferroelectric CuInP_2_S_6_, where the atomically-sharp interface yields unexpectedly large doping and high-quality plasmons, despite orders-of-magnitude smaller polarizations compared to oxide ferroelectrics. Infrared nano-imaging directly visualizes graphene plasmons whose doping depends on the adjacent ferroelectric layer. Combined nano-infrared and piezoresponse force microscopies reveal that graphene doping and CuInP_2_S_6_ polarization increase with thickness and saturate in tandem, linking charge-transfer doping and out-of-plane ferroelectricity. Our results establish ferroelectric-assisted charge-transfer plasmons in graphene/CuInP_2_S_6_ as a promising route to probe and control nanoscale ferroelectric textures.

## Abstract (web summary)

The authors show that a van der Waals ferroelectric material efficiently transfers charge to graphene, creating high-quality plasmon polaritons whose properties vary with the ferroelectric material’s thickness and polarization.

## Introduction

Charge transfer at interfaces typically occurs when two materials with dissimilar work functions (ϕ) are brought into contact. As vdW heterostructures can be assembled from atomically thin layers without lattice or symmetry matching^1–3^, they enable efficient interfacial charge transfer that dictates various optical^4,5^ and electronic^6–8^ behaviors. The remarkable efficiency of charge transfer at vdW interfaces also offers unique opportunities for engineering local doping at high densities without causing structural damage^9–13^. For instance, interfacing graphene (ϕ = 4.6 eV) with high-work-function vdW materials such as RuCl_3_ (ϕ = 6.1 eV)^10^ enables the latter to act as charge acceptors. This leads to substantial hole doping in graphene, up to n≈3 × 10^13 ^cm^−2^ (corresponding to ${E}_{F}\approx 0.6\,{{{\rm{eV}}}}$), beyond what is achievable with typical electrostatic gating. The interfacial charge-transfer process also maintains the high carrier mobility of graphene, resulting in exceptionally high plasmonic quality factor in graphene^9,12^, matching that of the highest quality hBN-encapsulated graphene devices^14^. However, the amount of charge transfer is anchored by the work-function difference and is not tunable once the heterostructure is fabricated.

Ferroelectric materials with spontaneous and switchable electric polarization offer tantalizing opportunities for massive and tunable charge transfer. Early work examined the use of a canonical ferroelectric PbZr_0.3_Ti_0.7_O_3_ (PZT) as a gate dielectric for tuning graphene plasmons^15^. However, the plasmonic contrasts were correlated with the corrugation of the PZT surface instead of its polarization, limiting its controllability. Ferroelectric control of carrier density through the polarization direction has been demonstrated in graphene on PZT^16,17^ and on LiNbO_3_^18^. However, the amount of charge carrier doping achieved is limited to $\sim 3\times {10}^{12}{{{\rm{c}}}}{{{{\rm{m}}}}}^{-2}$ due to surface effects^18^, well below the theoretical limit given the large electric polarization of LiNbO_3_ (*P*≈86 ${{{\rm{\mu }}}}{{{\rm{C}}}}/{{{\rm{c}}}}{{{{\rm{m}}}}}^{2}$). The advent of vdW ferroelectrics^19,20^ with atomically-sharp interfaces and out-of-plane polarizations, such as CuInP_2_S_6_^21–23^ and *α*-In_2_Se_3_^24,25^, unlocks a new paradigm for ferroelectric tuning of high-quality charge-transfer plasmons in graphene^26,27^. Here, we demonstrate prominent and tunable charge transfer in graphene coupled to a prototypical vdW ferroelectric CuInP_2_S_6_ (CIPS)^21–23^. High-quality plasmon polaritons in hBN/graphene/CIPS heterostructures are visualized directly through infrared nano-imaging^28,29^, complemented by transport, Raman spectroscopy, and piezoresponse force microscopy (PFM) measurements. Importantly, both charge transfer and polarization rise with CIPS thickness and eventually saturate, pointing to a ferroelectric-assisted charge-transfer (FACT) plasmonic response. We establish FACT plasmons as charge-transfer graphene plasmons whose carrier density and dispersion are controlled by ferroelectric polarization, beyond interfacial work-function mismatch.

Bulk CIPS is a layered ferroelectric semiconductor with a transition temperature (${T}_{{{{\rm{c}}}}}$) between 310‒315 K^22,30–35^ (see Supplementary Figs. S12, S13) and a spontaneous polarization of *P*≈5 ${{{\rm{\mu }}}}{{{\rm{C}}}}/{{{\rm{c}}}}{{{{\rm{m}}}}}^{2}$ at room temperature^23,36^. The vdW gap and weak interlayer coupling endow CIPS with various unusual ferroelectric properties, such as giant negative piezoelectricity^21,37–39^ and a quadruple-well energy profile^23^. The ferroelectricity in CIPS is mainly attributed to the displacement of Cu^+^ ions along the out-of-plane direction^21,23^. When ferroelectric CIPS is interfaced with graphene, the out-of-plane polarization field *P* within CIPS is expected to induce charge doping in graphene. This charge transfer in turn suppresses the depolarization field^40,41^ in CIPS and stabilizes ferroelectricity in the 2D limit^26,42^, a long-standing challenge in thin-film oxide ferroelectrics^43–45^. In the ideal case of graphene screening all the polarization charges (surface charge density ${\sigma }_{b}=\pm P$), the upper limit of charge doping for the graphene/CIPS (G/CIPS) structure can be estimated as $n={\sigma }_{b}/e\approx 3.1\times {10}^{13}{{{\rm{c}}}}{{{{\rm{m}}}}}^{-2}$, corresponding to a maximum Fermi energy $|{E}_{F}|\approx 0.65\,{{{\rm{eV}}}}$. Previous studies of graphene/CIPS heterostructures indeed show varying degrees of charge doping in graphene^27,46,47^, and the polarization of CIPS is anticipated to increase with layer thickness^33,48^. However, the expected increase of charge transfer in graphene with increasing CIPS thickness^27,42^ has not been demonstrated experimentally.

## Results

**Fig. 1:**
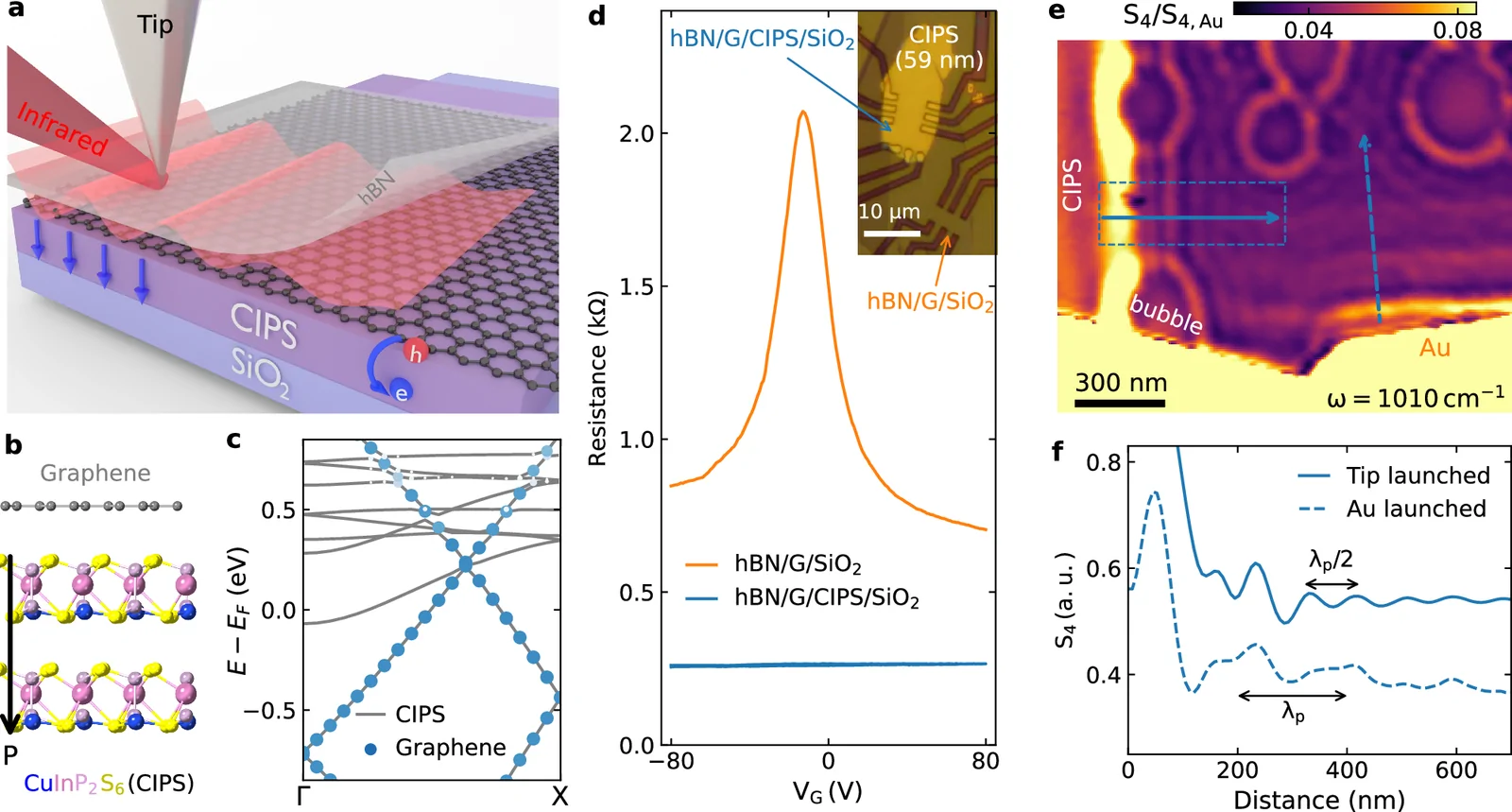
Charge-transfer plasmons in graphene on vdW ferroelectric CuInP_2_S_6_ (CIPS). **a** Schematic of the charge-transfer process at the graphene/CIPS interface and the nano-infrared measurements. **b** Side view of the lattice structure of graphene (gray) on bilayer CIPS, featuring mobile Cu atoms (blue) embedded in the vdW layer formed by In (pink), P (purple), and S (yellow) atoms. The black arrow indicates the polarization direction of CIPS. **c** Calculated band structure of graphene/2L-CIPS structure shown in (**b**). The graphene Dirac bands are colored in blue and show clear hole doping. **d** Resistance as a function of back-gate voltage (V_G_) for graphene on SiO_2_/Si substrate (orange curve) and for graphene/CIPS/SiO_2_/Si (blue curve). Both graphene devices are covered with top hBN (inset). The hBN/G/SiO_2_ device shows only a slight doping with the charge neutrality peak close to V_G_ = 0 V, whereas the hBN/G/CIPS device shows negligible gate-dependence and indicates a high doping level. **e** Au-normalized near-field amplitude (${S}_{4}/{S}_{4,{Au}}$) of a G/CIPS structure showing oscillations originating from SPPs launched by the metallic tip and the Au edge. **f** Line profiles of the tip-launched (solid line) and Au-launched plasmon polaritons (dashed line) along the paths indicated in panel (**e**) at the laser frequency of $\omega=1010{{{\rm{c}}}}{{{{\rm{m}}}}}^{-1}$.

In this work, thin flakes of CIPS were exfoliated from bulk crystals of CIPS onto SiO_2_/Si substrates, with thicknesses ranging from ~ 4 nm to 180 nm. Monolayer graphene was exfoliated and picked up with thin hBN and transferred on top of the CIPS flakes (see Methods). In Fig. 1a, we show a schematic of the infrared nano-imaging experiment on the hBN/G/CIPS sample, where the infrared laser is focused on the apex of the metallic tip that launches and collects nanoscale plasmon polaritons^9,49,50^. DFT calculations of the G/CIPS heterostructure (Fig. 1b) predict a modest charge transfer that hole-dopes graphene slightly (${E}_{F}\approx 0.2\,{{{\rm{eV}}}}$, Fig. 1c), and the charge transfer vanishes when the polarization direction switches (*P* points toward graphene, see Supplementary Fig. S15). This polarization-dependent charge transfer is consistent with recent studies of vdW ferroelectric interfaces, where interfacial charge redistribution was shown to tune out-of-plane dipoles and polarization^51^. This small charge doping in graphene can be checked directly by comparing transfer curves of field-effect transistor devices of bare graphene and hBN/G/CIPS on the same SiO_2_/Si substrate (inset of Fig. 1d). As shown in Fig. 1d, while the pristine graphene (orange curve) exhibits a resistive Dirac peak near zero gate voltage, the hBN/G/CIPS device (blue curve) shows negligible gate tunability across − 80 to 80 V, indicative of high charge doping. Such high charge-transfer doping leads to plasmonic features that can be visualized directly in real space. In Fig. 1e, we examined the near-field optical amplitude (${S}_{4}$, see “Methods”) near an Au contact region of the same sample at $\omega=1010\,{{{\rm{c}}}}{{{{\rm{m}}}}}^{-1}$, and observed prominent fringe patterns characteristic of charge-transfer plasmons^9,12,13^. Line profiles (Fig. 1f) perpendicular to the graphene edge (solid blue line) and the Au edge (dashed blue line) further reveal different periodicities that correspond to tip-launched and Au-launched plasmon polaritons, respectively.

**Fig. 2:**
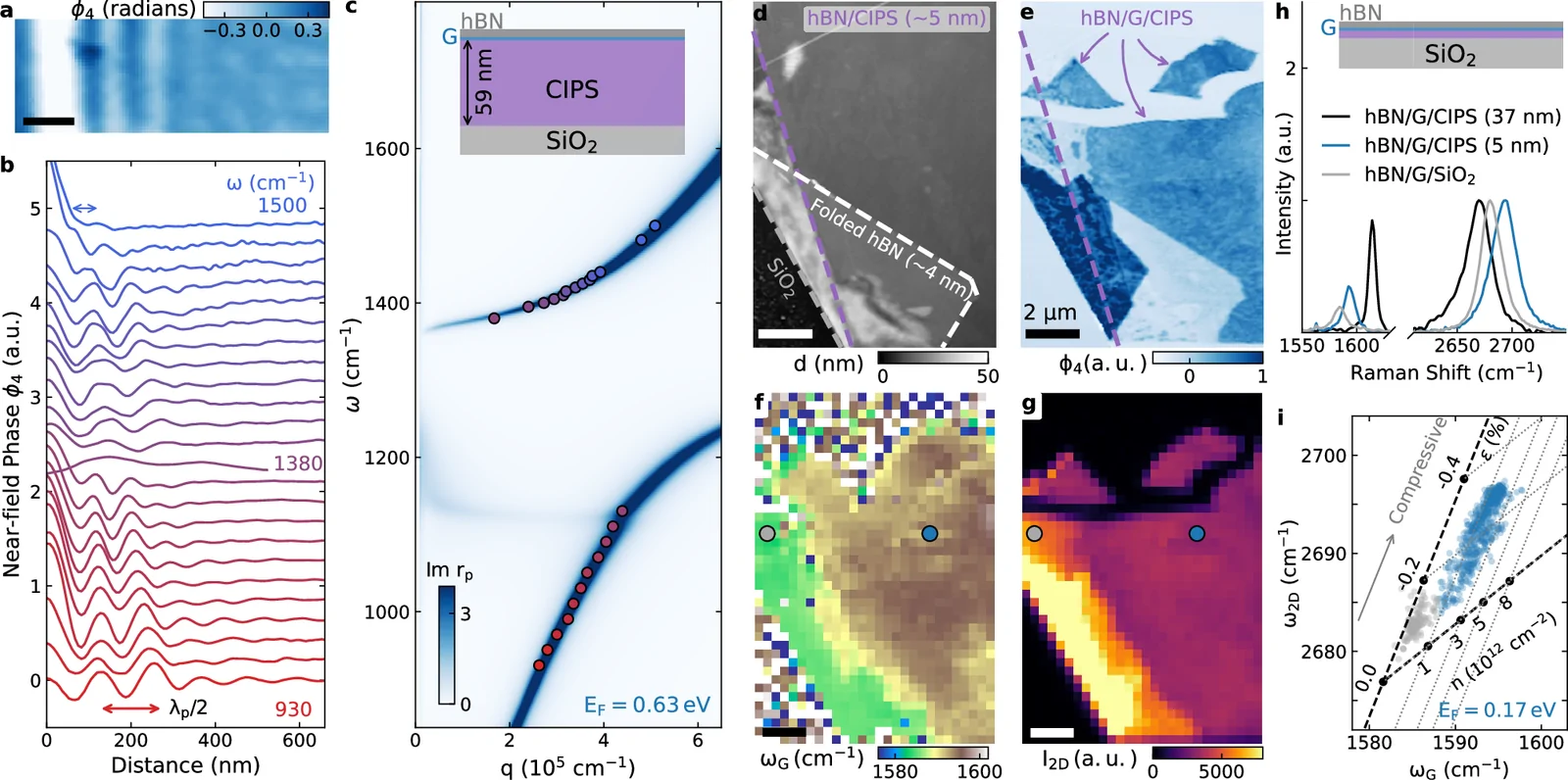
Tunable charge-transfer doping in G/CIPS with layer thickness. **a** Near-field phase (${\phi }_{4}$) for the hBN/G/CIPS (59 nm) sample in Fig. 1e inside the dashed rectangle region. Scale bar indicates 100 nm. **b** Line profiles of the averaged ${\phi }_{4}$ in panel (**a**) as a function of distance from the left graphene edge, showing a systematic change in polariton wavelength with laser frequency ($\omega=930-1500\,{{{\rm{c}}}}{{{{\rm{m}}}}}^{-1}$). Line profiles are shifted vertically for clarity. **c** The extracted polariton momentum ${q}_{p}=2\pi /{\lambda }_{p}$ from (**b**) (colored dots), overlaid on the theoretical model of the imaginary part of the reflection coefficient Im r_p_ for the heterostructure (inset). The Fermi energy of graphene used in the model is ${E}_{F}=0.63{{{\rm{eV}}}}$. **d** Topography map of G/CIPS ( ~ 5 nm) covered by thin hBN (~ 4 nm). Purple and gray dashed lines indicate the boundary of CIPS and SiO_2_, respectively. The white dashed line marks part of hBN that folded on top of the hBN/G/CIPS region. **e** Near-field phase of the same region in (**d**), showing clear contrast in graphene regions (blue) but without clear polariton fringes. The Raman maps of the fitted G peak position (${\omega }_{G}$) and the intensity of the 2D peak (${I}_{2D}$) on the same region are shown in (**f**, **g**), respectively. Scale bars in (**d**–**f**) indicate 2 ${{{\rm{\mu }}}}{{{\rm{m}}}}$. **h** Raman spectra of graphene in the frequency range of G and 2D modes at the gray (hBN/G/SiO_2_) and blue (hBN/G/CIPS (5 nm)/SiO_2_, top inset) dots in (**f**) are shown as gray and blue curves. Raman spectrum of another sample hBN/G/CIPS (37 nm)/SiO_2_ is also shown for comparison. **i** Correlation between the graphene G and 2D Raman modes, showing a slight doping of graphene on ~ 5 nm thin CIPS, corresponding to ${E}_{F}=0.17{{{\rm{eV}}}}$.

Remarkably, the observed plasmon polariton wavelength (Fig. 1f, ${\lambda }_{p}\approx 200\,{{{\rm{nm}}}}$ at $1010\,{{{\rm{c}}}}{{{{\rm{m}}}}}^{-1}$) in hBN (4 nm)/G/CIPS (59 nm) is large^9,49^ and indicates a doping level far exceeding the calculated ${E}_{F}\approx 0.2\,{{{\rm{eV}}}}$ for G/2 L CIPS. To extract more precisely the charge-transfer doping, we carried out frequency-dependent nano-imaging experiments to examine plasmonic dispersions. In Fig. 2a, we focus on the relatively homogeneous edge region marked by the dashed rectangle in Fig. 1e and show the near-field phase contrast (${\phi }_{4}$) that peaks at the edge^52,53^. The averaged line profiles in this region as a function of laser frequencies (Fig. 2b) show a systematic decrease in ${\lambda }_{p}$ from ω=930 to 1150 ${{{\rm{c}}}}{{{{\rm{m}}}}}^{-1}$ and from ω=1395 to 1500 ${{{\rm{c}}}}{{{{\rm{m}}}}}^{-1}$. The monotonic decrease of ${\lambda }_{p}$ with increasing frequency is noticeably disrupted above 1380 ${{{\rm{c}}}}{{{{\rm{m}}}}}^{-1}$ due to hybridization with hBN phonons^14,54^. In Fig. 2c, the extracted polariton momenta (${q}_{p}=2\pi /{\lambda }_{p}$) from Fig. 2b are organized in the dispersion ($\omega,{q}$) plot and overlaid on the calculated imaginary part of the reflection coefficient Im r_p_ (color map). The Im r_p_ map reflects divergence in r_p_ and provides an instructive way to both visualize the plasmonic dispersion and to extract the charge doping level in graphene^9,14^. The colormap is calculated for hBN (4 nm)/G/CIPS (59 nm) on a SiO_2_/Si substrate (Fig. 2c inset) using experimental dielectric functions for CIPS (see Supplementary Fig. S2). The extracted Fermi energy of graphene that provides the best agreement with the data (colored dots) is ${{|E}}_{F}|=0.63$ eV ($n\approx 2.92\times {10}^{13}{{{\rm{c}}}}{{{{\rm{m}}}}}^{-2}$). Bubble-reflected^9,55^ plasmon fringes in the nearby region (Fig. 1e) yield a consistent ${{|E}}_{F}|=0.6$ eV (see Supplementary Fig. S10), confirming the high doping extracted from edge-reflected plasmons. Such high doping levels in G/CIPS would require a back gate voltage ${{|V}}_{g}|\approx 385$ V to reach the Dirac peak for 285-nm-thick SiO_2_ dielectric^49^, consistent with the negligible gate-dependence within $\pm \,80$ V shown in Fig. 1d.

Interestingly, the charge-transfer doping in graphene on ultrathin CIPS is greatly reduced. The topography image and the corresponding near-field phase (${\phi }_{4}$) image of a hBN/G/CIPS (5 nm) heterostructure are shown in Fig. 2d and Fig. 2e, respectively. In both images, CIPS is located on the right of the purple dashed line, and the hBN/G/CIPS regions show clear contrast in ${\phi }_{4}$. Although the near-field phase contrasts of graphene do not display well-defined polariton fringes as shown in Fig. 2a, the short-wavelength inhomogeneities (Fig. 2e) are characteristic features of strong scattering^56^ and/or low charge doping^57^ for graphene plasmons. To probe the low charge doping level in the hBN/G/CIPS (5 nm), we employed confocal Raman spectroscopy (see Methods) combined with correlation analysis of the G and 2D modes^58,59^. The fitted graphene G mode frequency map for the same sample area as Fig. 2d, e is presented in Fig. 2f. A distinct boundary is observed: the pristine hBN/G/SiO_2_ region shows ${\omega }_{G}\approx 1584{{{\rm{c}}}}{{{{\rm{m}}}}}^{-1}$ (green), while the hBN/G/CIPS (5 nm)/SiO_2_ region exhibits ${\omega }_{G}\approx 1593{{{\rm{c}}}}{{{{\rm{m}}}}}^{-1}$ (brown). The 2D mode intensity map ${I}_{2D}$ (Fig. 2g) also shows good correlation with the graphene regions mapped through near-field phase in Fig. 2e. Representative point spectra from pristine graphene (gray) and thin-CIPS-contacted graphene (blue) are shown in Fig. 2h, along with the spectrum from graphene on thicker CIPS (37 nm, black) for comparison. Both the pronounced upshift in ${\omega }_{G}$ and the reduced 2D/G intensity ratios (${I}_{2D}/{I}_{G}$) indicate enhanced charge doping^60,61^ in graphene on thicker CIPS. To further separate the impacts of strain and doping, we plot the established ${\omega }_{G}$ and ${\omega }_{2D}$ peak shift trajectories for pure strain and pure doping^58,59^ in Fig. 2i (black dashed lines), together with the peak shifts obtained from Raman mapping (Fig. 2f). Clusters of gray and blue dots represent the pristine graphene regions with negligible doping and hBN/G/CIPS (5 nm) regions with a slight doping of $\sim 2-3\times {10}^{12}{{{\rm{c}}}}{{{{\rm{m}}}}}^{-2}$ (${E}_{F}\approx 0.16-0.20{{{\rm{eV}}}}$), respectively, in close agreement with the DFT prediction for G/2 L CIPS (Fig. 1c).

The impact of CIPS thickness on charge-transfer doping levels in graphene indicates a connection between the charge-transfer and the ferroelectric polarizations. Indeed, CIPS exhibits various thickness-dependent phenomena^38,48^ originating from its ferroelectric polarizations. Early PFM measurements have shown the gradual suppression of the out-of-plane ferroelectric polarizations of CIPS in the thin limit^21,33^, quantified by the piezoelectric coefficient^45^. Moreover, recent studies demonstrate that the work function of CIPS depends sensitively on its thickness^62^, which directly impacts the charge-transfer process. To further demonstrate the ferroelectric-assisted nature of the charge-transfer plasmon at the graphene/CIPS interface, we fabricated CIPS/graphene/SiO_2_ (CIPS/G) structures that allow for co-located mapping of plasmonic responses and piezoelectric responses.

**Fig. 3:**
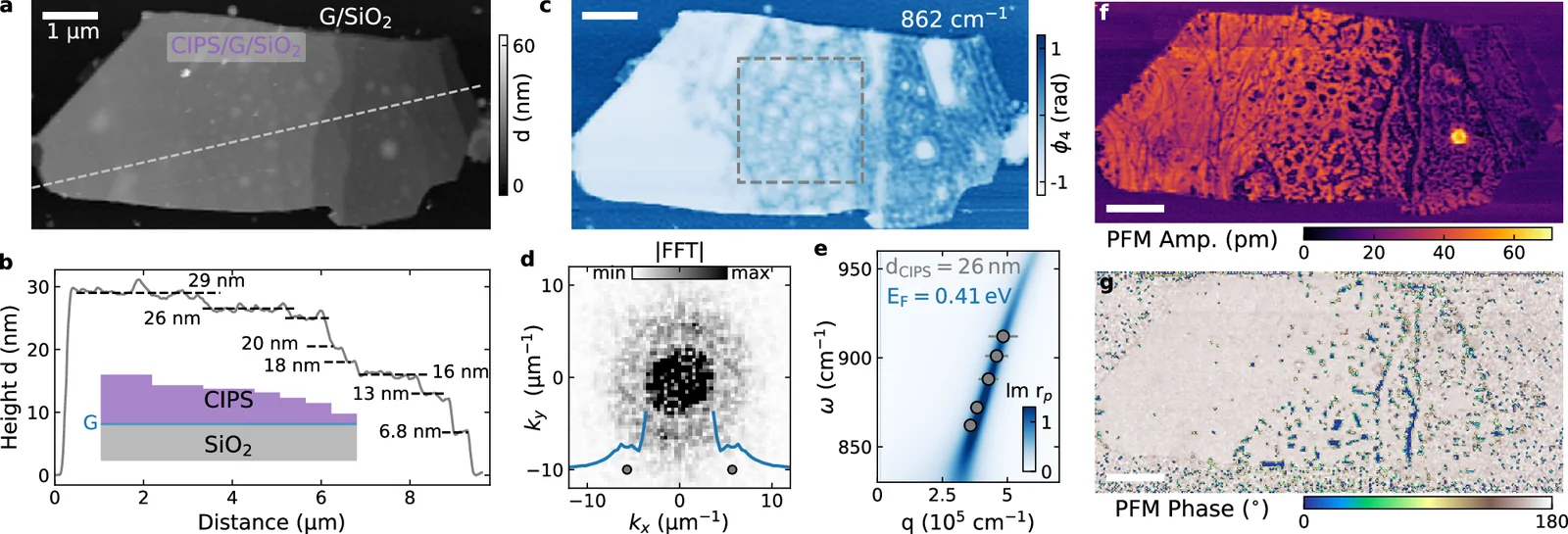
Correlation between charge transfer and ferroelectricity in CIPS/G. **a** Topography image of a CIPS/graphene sample with varying CIPS thickness on SiO_2_/Si substrate. **b** The line profile along the white dashed line in (**a**). Inset is a schematic of the CIPS terraces on graphene/SiO_2_. **c** Near-field phase (${\phi }_{4}$) of the CIPS/G sample at $\omega=862\,{{{\rm{c}}}}{{{{\rm{m}}}}}^{-1}$, showing short-wavelength inhomogeneities that also vary with CIPS thickness. **d** Fast Fourier transform (FFT) amplitude of the gray dashed region in (**c**) (26 nm-thick CIPS). The radially averaged line profile (blue lines) shows a weak mode (gray dots) indicative of the plasmon polaritons. **e** Extracted polariton momenta (${q}_{p}=2{\pi k}_{p}$) as a function of laser frequency (gray dots), overlaid on the Im r_p_ calculation. The modeled Fermi energy in graphene is 0.41 eV. **f** PFM amplitude map of the same CIPS/G sample, showing nanoscale domains and a gradual decrease in piezoresponse with decreasing CIPS thickness. **g** Corresponding PFM phase map, showing domains predominantly aligned along one polariz**a**tion direction. Scale bars in (**a**, **c**, **f** and **g**) indicate 1 ${{{\rm{\mu }}}}{{{\rm{m}}}}$.

The topography image of a terraced CIPS/G sample is displayed in Fig. 3a, with the thickness of CIPS decreasing from 29 nm to ~ 7 nm from left to right (Fig. 3b). The near-field phase (${\phi }_{4}$) data in Fig. 3c show rich and inhomogeneous patterns, characteristic of charge-transfer plasmons scattering off bubbles at the interface^9,12^. Although clear plasmonic fringes are not evident, the plasmon polariton momentum can still be extracted via a 2D fast Fourier transform (FFT) of the near-field images^56,57^. In Fig. 3d, we show the 2D FFT amplitude on a linear scale for the gray dashed region in Fig. 3c. The FFT amplitude reveals a weak ring feature, with a radius ${k}_{p}$ that can be more clearly identified by the radially averaged line profile (blue lines in Fig. 3d). Both the real-space pattern and the FFT exhibit expected dispersion with laser frequencies (see Supplementary Fig. S9). The plasmonic dispersion allows the determination of graphene Fermi energy (${E}_{F}\approx 0.41\,{{{\rm{eV}}}}$) for this CIPS/G region when compared to Im r_p_ simulations, as shown in Fig. 3e. The lack of plasmonic features for thicker CIPS (29 nm) on graphene is also explained by the suppressed Im r_p_ modeled with the same doping level (Supplementary Fig. S21).

The exposed CIPS surfaces in the CIPS/G structure also allow direct PFM measurements on the same sample. The PFM amplitude and phase data are shown in Fig. 3f, g, respectively. The overall PFM amplitude decreases in thinner CIPS (Fig. 3f), consistent with previous reports^21,33,48^. Interestingly, the PFM amplitude shows “bubble-like” features reminiscent of the plasmonic response (Fig. 3c), yet distinct from conventional ferroelectric domains in bare CIPS^21,63^. The PFM phase of CIPS/G further reveals a nearly-uniform single domain^62^, with the ferroelectric polarization aligned in the same direction (see also Supplementary Fig. S17). The nearly-uniform single domains in thin CIPS ( < 100 nm) can also be verified in second harmonic generation measurements (Supplementary Fig. S11), which provide an independent probe of the ferroelectric polarizations^21,64^.

**Fig. 4:**
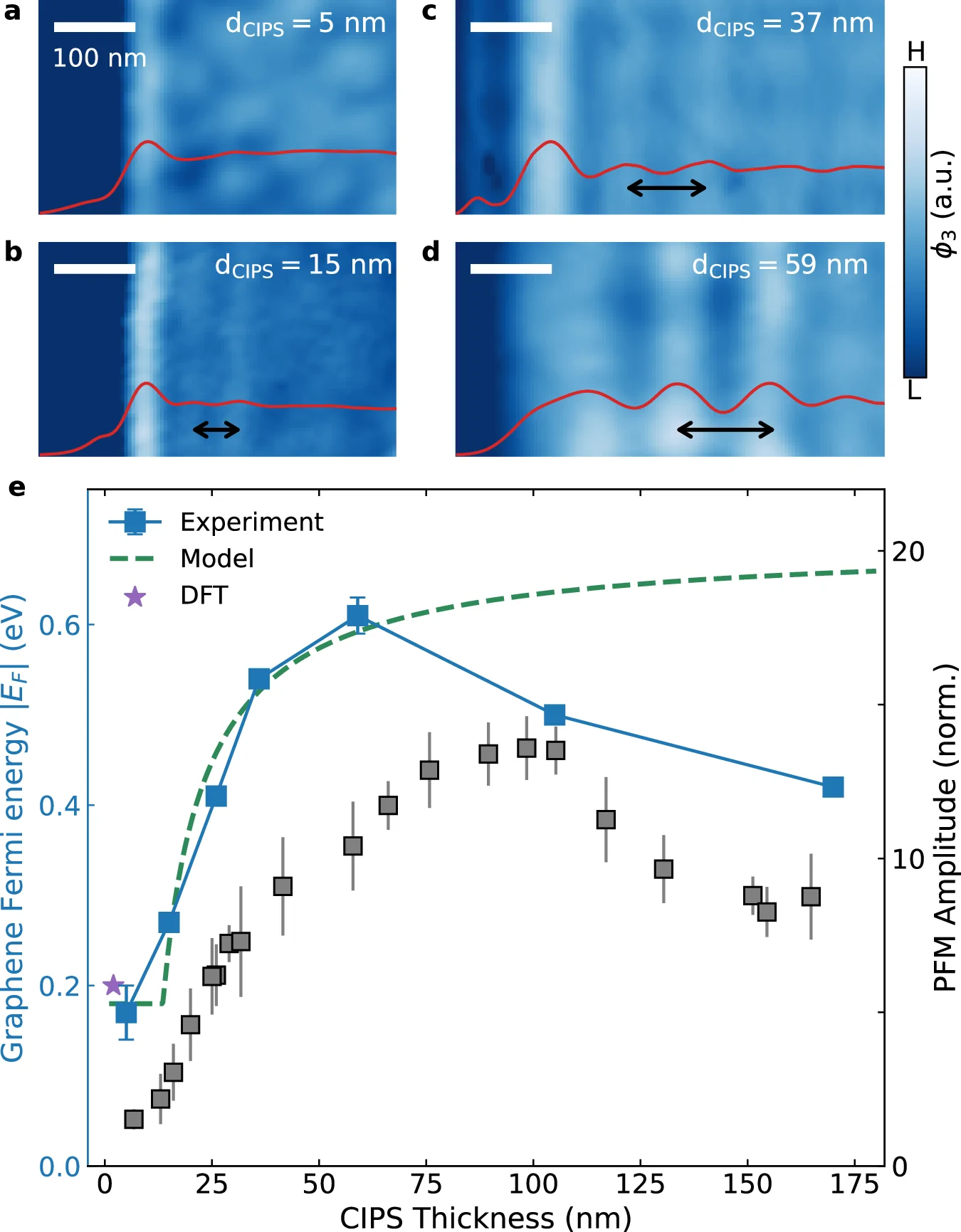
Ferroelectric-assisted charge transfer (FACT). **a**–**d** Near-field phases for the G/CIPS samples with CIPS thicknesses of 5 nm (**a**), 15 nm (**b**), 37 nm (**c**), and 59 nm (**d**). Red curves are the averaged line profiles of the images, and the black arrows indicate the plasmon polariton half wavelength (${\lambda }_{p}/2$). The laser frequencies used were $\omega=900\,{{{\rm{c}}}}{{{{\rm{m}}}}}^{-1}$ except in (**d**) $\omega=930{{{\rm{c}}}}{{{{\rm{m}}}}}^{-1}$. Scale bars indicate 100 nm. **e** Summary of the graphene Fermi energy ($|{E}_{F}|$) for G/CIPS and CIPS/G samples as a function of CIPS thickness (blue squares, left axis). The green dashed line shows the graphene $|{E}_{F}|$ from the electrostatic model (see Supplementary Fig.  S16). The purple star indicates the DFT prediction for the graphene $|{E}_{F}|$ in G/2 L CIPS. Gray squares represent the PFM amplitude (right axis, normalized by the bias voltage) for CIPS of various thicknesses, showing an increase with thickness up to around 100 nm, followed by a slight decrease at larger thicknesses. Error bars reflect uncertainties from plasmon-wavelength extraction, comparison with calculations, and/or areal statistics across regions of the same thickness.

We summarize in Fig. 4 the observed thickness-dependent behavior of both charge transfer in graphene and the piezoresponse of CIPS. In the thin limit (~5 nm CIPS), the plasmonic fringes are not well developed (Fig. 4a and Fig. 2e) and the doping level ${E}_{F}\approx 0.16-0.20\,{{{\rm{eV}}}}$ is extracted from Raman analysis (Fig. 2i). For thicker CIPS (Figs. 4b-4d), clear plasmonic fringes are present in the G/CIPS structures. Since the plasmon polariton wavelength in graphene scales as ref. ^65^ ${\lambda }_{p}\propto \sqrt{n}\propto {E}_{F}$, the observed increase in ${\lambda }_{p}$ for G/CIPS structures with increasing CIPS thickness d (black arrows in Fig. 4b–d) directly reflects enhanced charge-transfer doping for $d < 60\,{{{\rm{nm}}}}$.

Remarkably, as shown in Fig. 4e, the increase in charge-transfer doping with CIPS thickness (blue squares) closely follows the enhancement of the PFM amplitude (gray squares) for CIPS thicknesses below ~ 100 nm. The PFM amplitude, normalized by tip bias voltage, provides an accurate local measure of the ferroelectric polarization^66,67^. Microscopically, the out-of-plane CIPS polarization produces bound surface charge, which is partially screened by graphene and thereby modifies the graphene/CIPS interfacial electrostatic potential. An electrostatic model of FACT plasmons in graphene on single-domain CIPS captures the rise of |${E}_{F}$| below 100 nm, as shown by the green dashed line (see also Supplementary Information Sec. VII and Supplementary Fig. S16). This FACT mechanism is further supported by domain-resolved PFM and near-field measurements (see Supplementary Fig. S14), which show different graphene doping levels on opposite CIPS polarization domains within the same graphene/CIPS heterostructure.

Interestingly, both the charge-transfer doping level in graphene and the PFM amplitude in CIPS saturate and then decrease slightly above ~ 100 nm, as shown in Fig. 4e. While the saturation of PFM amplitude in CIPS above ~ 100 nm has been reported^48^ (Supplementary Fig. S17), the saturation of the charge-transfer doping in graphene with substrate thickness is rare. The saturation and slight reduction of the charge-transfer doping in G/CIPS heterostructures atd> 100 nm can be attributed to the presence of domains with opposite out-of-plane polarizations, which are more prevalent in thicker CIPS flakes (see Supplementary Fig. S17d–f). In addition, in-plane polarizations in CIPS have also been found to coexist with out-of-plane polarizations in CIPS films thicker than 90 nm^68^, which will further complicate the charge-transfer process at the graphene/CIPS interface. The strong correlation between the charge-transfer doping and ferroelectric polarization, observed both in the growth and saturation regions, underscores the ferroelectric-assisted nature of the observed plasmons.

In summary, we have demonstrated that the interfacial charge transfer process, long regarded as an intrinsic surface phenomenon dictated by work function differences, can be actively tuned at the graphene/CIPS interface through the thickness of CIPS. Beyond thickness, the ferroelectric polarization in CIPS can also be tuned electrically^21,27^ and mechanically (flexoelectricity)^69,70^, offering several non-volatile knobs for engineering charge-transfer plasmons in graphene. Furthermore, the coexistence of in-plane and out-of-plane polarizations in thicker CIPS^68^ supports polarization rotations and skyrmion-like topological textures^63,71^, enabling plasmonic readout of nanoscale polarization bubbles via FACT plasmons in graphene/CIPS. Our work establishes the FACT plasmon as a platform that intertwines a high-quality vdW interface with the rich tunability offered by ferroelectricity. The FACT plasmon response in completed heterostructures remains stable within experimental uncertainty over six months (Supplementary Fig. S20), providing initial evidence of device-level stability and motivating future retention and endurance studies. Ferroelectric control of polaritons at the nanoscale, as demonstrated here, opens new opportunities for high-density, reconfigurable, and on-chip nanophotonic devices.

## Methods

### Synthesis of CuInP_2_S_6_ crystals

The reagents, copper powder (99.999%, -100 mesh, Alfa Aesar), indium ingot (99.999%, Alfa Aesar), red phosphorus chunks (99.99+%, Sigma Aldrich) and sulfur pieces (Alfa Aesar, 99.9995%) were used as received with no further purification. The indium metal was cut into ~ 1 mm size pieces to increase surface area. Single crystals of CuInP_2_S_6_ were synthesized using a modified reactive flux method^72^ described as follows. Copper (0.1504 grams), indium (0.2717 grams), phosphorus (0.4398 grams), and sulfur (1.1382 grams) were added in the ratio of 1:1:6:15 (Cu:In:P:S) (total mass: 2.000 grams) into a fused silica tube of outer and inner diameter of 12.7 mm and 10.5 mm, respectively. The tube was flame sealed at a pressure of ~ 30 mTorr and to a length of 10 cm. The tube was placed upright in a computer-controlled box furnace so that the reaction mixture would remain at the bottom of the tube. The furnace was then programmed to the following heating profile: Heat to 350 °C in 6 h, dwell for 12 h, heat to 665 °C in 12 h, dwell for 240 h, cool to ambient temperature over 12 h. CAUTION:[*The reaction of ethanol and P/S compounds can produce pungent and hazardous thiol-containing volatile compounds*.] All manipulations involving the removal of the P/S flux were performed in a well-ventilated fume hood. The silica ampoule was opened, and the extracted ingot was placed in a 20 mL scintillation vial with pure ethanol (~ 10 mL). The P/S flux was slowly dissolved with ethanol at ~ 50 °C. Over the course of a few hours, the reacted ethanol was decanted, and fresh ethanol aliquots were added every ~ 15 minutes. After the bubbling stopped and the P/S flux appeared to be fully removed, the product with fresh ethanol was allowed to sit overnight to remove residual P/S flux. The solution was then decanted, and the product was washed several times with ethanol, followed by acetone. The uncapped vial with the product was left open for ~ 24 h to off-gas residual volatiles from the product. Thin yellow platelets of CuInP_2_S_6_ were mechanically isolated from the main mass of crystals for exfoliation.

### Device fabrication and transport characterization

The device fabrication process is described below. CuInP_2_S_6_ (CIPS), hBN, and graphene were mechanically exfoliated onto SiO_2_ (285 nm)/Si substrates from bulk crystals, respectively.

For the hBN/G/CIPS heterostructure, thin hBN (< 5 nm) and monolayer graphene were sequentially picked up using a polycarbonate (PC) dry pick-up^73^ at 100 °C. The resulting hBN/G stack was then placed onto an as-exfoliated CIPS flake at 200 °C. To form electrical contacts, small windows were opened by etching small regions of the top hBN and graphene above CIPS and directly SiO_2_ by reactive ion etching under CHF_3_ and O_2_ (45 sccm, 15 W RF power, 45 mTorr, 10 s). A second electron beam lithography step defined the metal leads, after which Au (100 nm)/Ti (2 nm) was deposited by electron beam evaporation. This geometry yields edge contacts to graphene^74^, and the two-step lithography keeps the leads on top of hBN, preventing short circuits (Fig. 1d, inset). Additional device-fabrication images, a process schematic, and AFM thickness characterization are provided in Supplementary Figs. S3–S5. Monolayer graphene was further verified by AFM and Raman spectroscopy (Supplementary Fig. S22).

For CIPS/G/SiO_2_ heterostructures, exfoliated CIPS flakes are directly transferred onto exfoliated graphene on SiO_2_/Si chips using PDMS. The PDMS residue is then rinsed by acetone and IPA.

Electrical measurements were performed in a vacuum probe station at room temperature and elevated temperatures using a semiconductor parameter analyzer (Keysight B1500A). Si was used for the back gate, and gate voltage (V_g_) was applied from − 80 V to + 80 V to avoid dielectric breakdown (285 nm SiO_2_) with 0.1 V of source-drain voltage (V_ds_).

### Scanning near-field optical measurements

Infrared nano-imaging was carried out at room temperature under ambient conditions using a scattering-type scanning near-field optical microscope (s-SNOM, Neaspec/Attocube). Tunable continuous-wave infrared lasers from Daylight Solutions were used and the laser light (with frequencies $\omega=860\,{{{\rm{c}}}}{{{{\rm{m}}}}}^{-1}$ to $1500\,{{{\rm{c}}}}{{{{\rm{m}}}}}^{-1}$) was focused onto commercial (Arrow) AFM probes with nominal tapping frequencies of $f=75\,{{{\rm{kHz}}}}$ or $285\,{{{\rm{kHz}}}}$. The near-field scattering amplitude (${S}_{n}$) and phase (${\phi }_{n}$) were collected simultaneously using a pseudo-heterodyne interferometric technique. The detected optical signal was demodulated at the third or fourth harmonic (*n* = 3 or 4) of the tip tapping frequency f to minimize far-field contributions to the scattered light. No external tip bias or gate bias was applied during the infrared nano-imaging measurements. Additional frequency-dependent near-field maps and dispersion analysis are provided in Supplementary Figs. S6–S8.

### Piezoresponse force microscopy measurements

The piezoresponse force microscopy (PFM) was performed using a Neaspec instrument with PFM capability and a Bruker Dimension Icon system at room temperature and under ambient conditions. The PFM measurements were conducted with an AC bias voltage of 1 V at 10 kHz – 20 kHz, far below the tip resonances (Arrow NCPt, resonance frequency $\sim 285\,{{{\rm{kHz}}}}$, spring constant ~ 42 N/m). No DC tip bias or gate bias was applied during the PFM measurement.

### Raman characterization

Raman mappings of the graphene/CIPS samples were performed using a commercial Renishaw inVia Raman spectrometer with 532 nm excitation (power ~ 0.3 mW) and collection in a 180-degree back-scatter geometry (1800 lines/mm grating). All Raman mapping measurements were taken under ambient conditions.

Temperature-dependent Raman measurements on thick CIPS flakes were performed in a Bruker Senterra microscope with 532 nm excitation (power ~ 20 µW). The CIPS flakes on SiO_2_/Si substrates were heated with a small metal ceramic heater (24 W), and the temperature was controlled by a Lakeshore 335 controller.

### Second harmonic generation (SHG) measurements

Temperature- and angle-dependent SHG measurements were carried out with 80 MHz femtosecond pulses (800 nm, 80 fs) generated by a Ti:sapphire oscillator (Tsunami, Spectra-Physics). The incident beam was first sent through a polarizer to ensure polarization purity. The incident beam was then reflected by a 650 nm short-pass dichroic mirror, sent through an achromatic half-waveplate, and subsequently focused onto the samples with a 50x objective. The SHG signal transmitted through the dichroic mirror and entered the photomultiplier tube (PMT). An additional 700 nm short-pass filter was placed before the PMT to reduce residual fundamental light. The power of the incident beam after being focused onto the samples was measured to be ~ 425 µW. All the samples were secured in an Oxford Instruments Microstat HiRes2 with a 0.5 mm thick UVFS window. Prior to cooling, the chamber was maintained at high vacuum, around 10^-5 ^mbar. The samples were cooled with liquid nitrogen and maintained at different temperatures with an Oxford Instruments MercuryiTC controller.

### Density functional theory calculations

The optimized atomistic and electronic structures were calculated by density functional theory (DFT) using the Vienna ab initio simulation package (VASP)^75^. The electronic wave function was described using projector-augmented wave (PAW) pseudopotential with the exchange correlation interaction treated using Perdew-Burke-Ernzerhof (PBE) method^76–78^. The weak van der Waals interactions between different layers were described with the zero-damping D3 method of Grimme (DFT-D3)^79^. The polarization properties were calculated using the Berry phase method^80^. The plane wave cutoff energy was set as 400 eV, while the convergence thresholds for force and energy were 0.01 eV/Å and 10^−5 ^eV, respectively. For the graphene/CIPS heterojunctions, a large vacuum layer of approximately 20 Å was used to avoid interactions between adjacent bulks. A 5 × 4 × 1 k-grid with dipole correction was used for the electronic structure calculations of the heterojunctions.

## Supplementary information

Supplementary Information

Transparent Peer Review file

## Source data

Source Data

## Data Availability

All data needed to evaluate the conclusions of this study are available within the paper, its Supplementary Information, and the Source Data file. Source data are provided in this paper.
