# Supplementary material for: Ferroelectric-assisted charge-transfer plasmons in graphene/CuInP2S6
Source: Nat Commun. 2026 Aug 17;17:10102. doi: 10.1038/s41467-026-76909-2 (PMC13597499; doi:10.1038/s41467-026-76909-2)
Supplement: Supplementary file 1 [file 41467_2026_76909_MOESM1_ESM.pdf]

Supplementary Information for: Ferroelectric-assisted charge-transfer (FACT) plasmons in graphene/$CuInP_2S_6$

Table of Contents

I. Scanning electron microscopy (SEM) and Energy-dispersive X-ray spectroscopy (EDS) Characterizations

II. Modeling of Reflectance and Transmission Spectra of $CuInP_2S_6$.

III. Device Fabrication Steps and Schematics

IV. Additional Infrared Nano-imaging maps

V. Second Harmonic Generation Mapping and Raman Spectra of exfoliated $CuInP_2S_6$ flakes

VI. Correlation between CIPS polarization domains and graphene doping

VII. Electrostatic model for ferroelectric-assisted charge transfer in G/CIPS

VIII. FACT plasmons in nanoscale structures and long-term stability

IX. Characterization of monolayer graphene

References

I. Scanning electron microscopy (SEM) and Energy-dispersive X-ray spectroscopy (EDS) Characterizations

Crystals of $CuInP_2S_6$ (CIPS) were mounted on an aluminum stub with conductive carbon tape. The crystals were exfoliated once with scotch tape to reveal a pristine surface. SEM images were measured using a ZEISS Sigma VP SEM with an accelerating voltage of 15 kV. Spectra of the emitted X-rays were collected using a Bruker XFlash® 6 | 30 detector. The accelerating voltage for EDS measurements was 15 kV. Elemental composition was determined by fitting the collected spectra with the Bruker ESPRIT 2 software package, as shown in Figure S1.

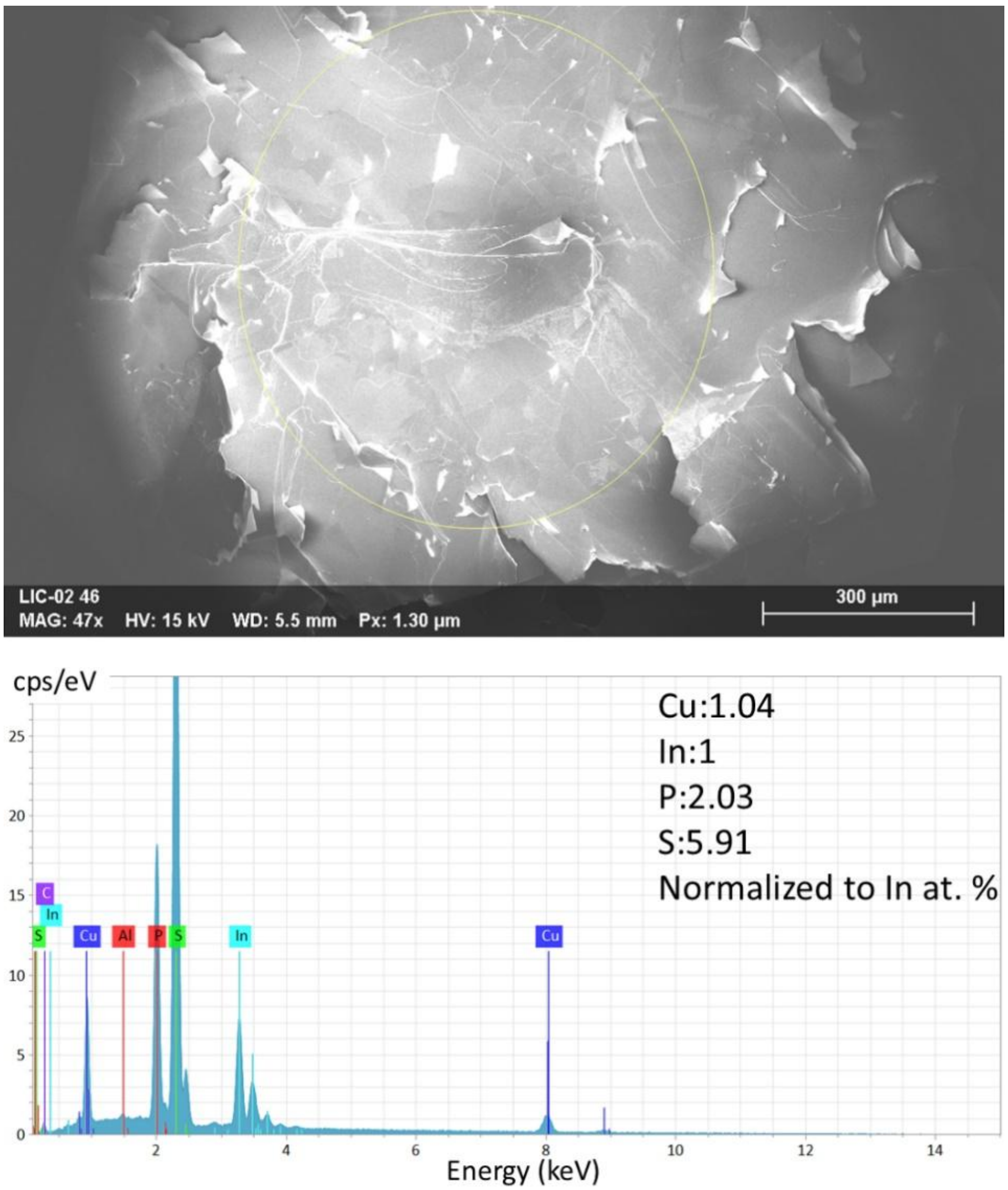

Figure S1. Scanning electron microscopy (SEM, top) overview and energy-dispersive X-ray spectroscopy (EDS, bottom) of a $CuInP_2S_6$ crystal. The yellow circle in the top panel indicates the area over which the EDS spectrum was acquired. Quantitative EDS analysis of the marked area gives $Cu_{1.04}InP_{2.03}S_{5.91}$(normalized to In = 1), consistent with the nominal $CuInP_2S_6$ stoichiometry.

II. Modeling of Reflectance and Transmission Spectra of $CuInP_2S_6$.

Optical reflectance and transmittance spectra on $CuInP_2S_6$ were measured using a Hyperion 2000 microscope coupled to a Bruker Vertex 80V Fourier-transform infrared spectrometer. A tungsten-halogen lamp served as the broadband light source, covering a photon energy range of ~0.3 to 2.5 eV. Unpolarized light was focused onto the sample with a 15X objective, and the microscope aperture was adjusted to be smaller than the sample area. The reflectance and transmittance spectra were normalized to a nearby bare substrate region. A mercury-cadmium-telluride detector and a silicon detector were used for the near-infrared and visible ranges, respectively. As shown in Figure S2, the broadband reflectance (blue) and transmission (orange) spectra of thick CIPS flake exfoliated on fused silica substrate show characteristic Fabry-Perot oscillations, consistent with the large bandgap[1,2] of CIPS ($E_g \approx 2.8$ eV ≈ 22583.5 cm^{-1}). Simultaneous fits (black dashed lines) of the reflectance and transmission spectra based on a multilayer model[3] capture the main spectral features and allow the extraction of the dielectric constant of CIPS.

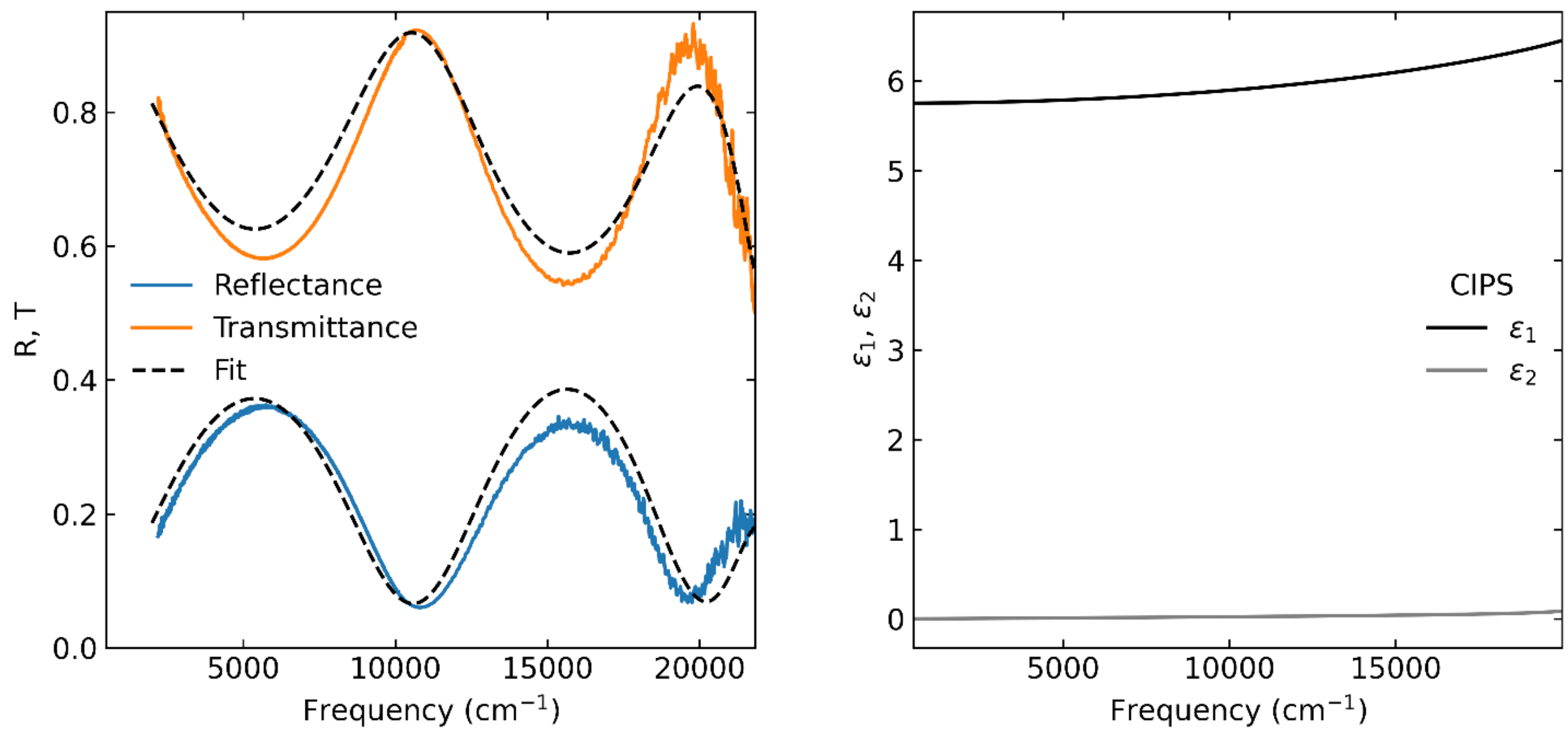

Figure S2. (Left) Broadband reflectance and transmission spectra on a thick CIPS flake exfoliated on a fused silica substrate. Black dashed lines indicate Lorentzian model fits to the reflectance and transmission spectra. (Right) The complex dielectric functions ($\varepsilon = \varepsilon_1 + i\varepsilon_2$) of CIPS extracted from the fitting with Lorentzian model.

The multilayer model consists of thin CIPS with thickness *d* on top of a ~500 µm thick fused silica substrate. The refractive index of fused silica is obtained from literature[4]. The dielectric responses of the CIPS layer as a function of frequency are parameterized by Lorentzian oscillators[5]:

$$\varepsilon(\omega) = \varepsilon_\infty + \sum_j \frac{\Omega_j^2}{\omega_j^2 - \omega^2 - i\gamma_j\omega}$$

where ω_j, Ω_j^2 and γ_j are the oscillator frequency, the oscillator strength and the linewidth of the jth oscillator. ε_∞ is the high-frequency dielectric permittivity that results from the high-energy transitions outside our measurement window. For CIPS, a single oscillator with $\omega_j = 29898\ \text{cm}^{-1}$, $\Omega_j = 23295\ \text{cm}^{-1}$, $\gamma_j = 2111.6\ \text{cm}^{-1}$ and $\varepsilon_\infty = 5.08$ captures the main spectral features below the bandgap (Figure S2).

III. Device Fabrication Steps and Schematics

In Figure S3 and Figure S4, we provide additional details regarding the device fabrication steps for the hBN/graphene/CIPS/SiO_2/Si heterostructure. In the hBN/G/CIPS devices, graphene is encapsulated by a thin top hBN layer. Since hBN is insulating, the contact regions must first be opened before metal deposition. We therefore used the first lithography step to define small contact windows, followed by reactive ion etching to locally expose the graphene edge/contact region. A second lithography step was then used to define the extended metal leads, followed by Ti/Au deposition. This process confines the metal–graphene contact to the pre-defined etched windows, while the larger metal leads remain on top of insulating hBN. Compared with a one-step process in which etching and metal deposition are performed using the same pattern, this two-step process provides more precise control of the contact area and effective channel geometry and reduces the possibility of unintended metal contact to graphene, leakage, or shorting. The thicknesses of the hBN and CIPS were measured by atomic force microscopy, as shown in Figure S5.

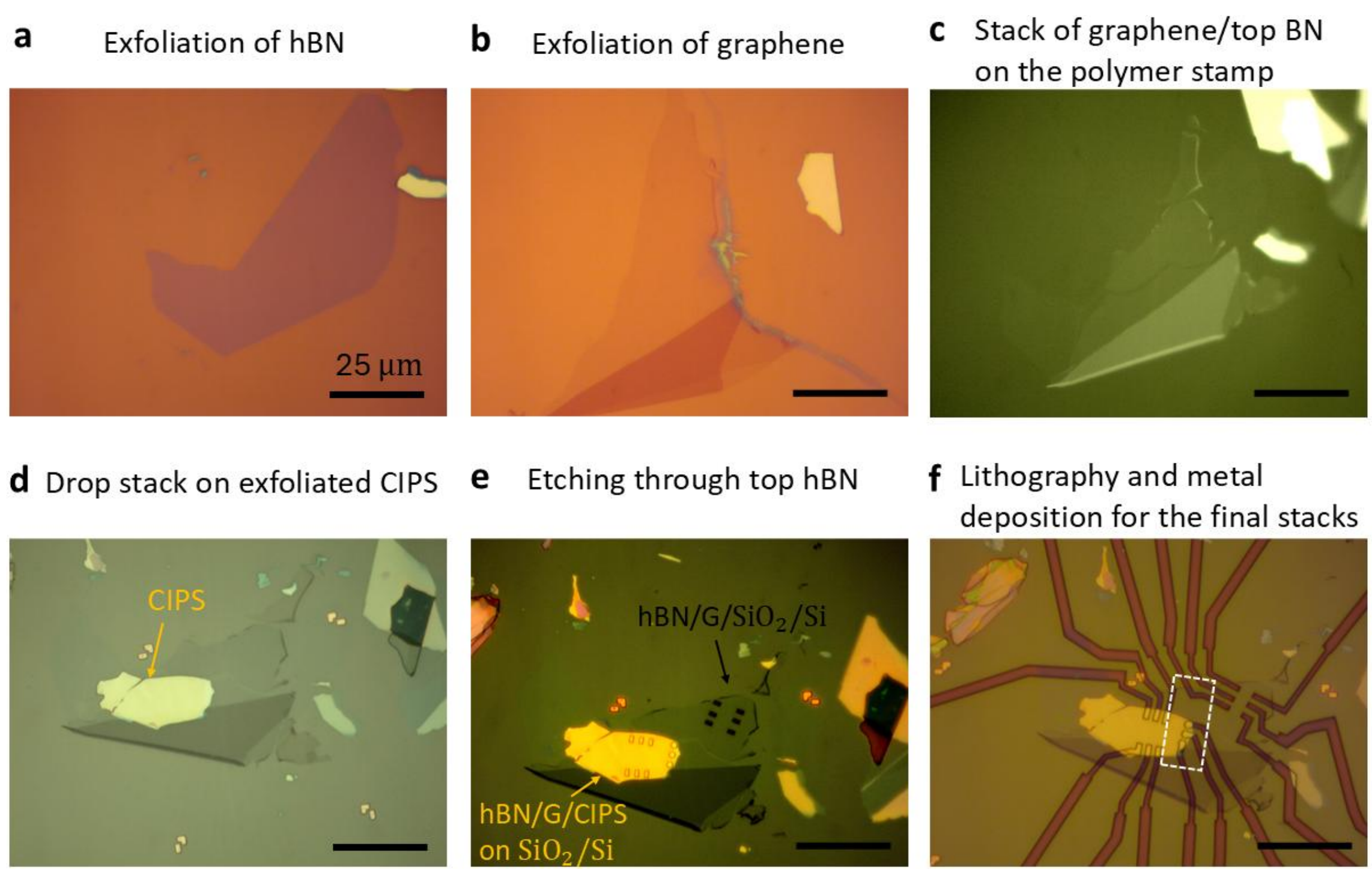

Figure S3. Optical images of the fabrication of hBN/graphene/CIPS/SiO_2/Si and hBN/graphene/SiO_2/Si heterostructures shown in Fig. 1 of the main text. Scalebars are all 25 µm.

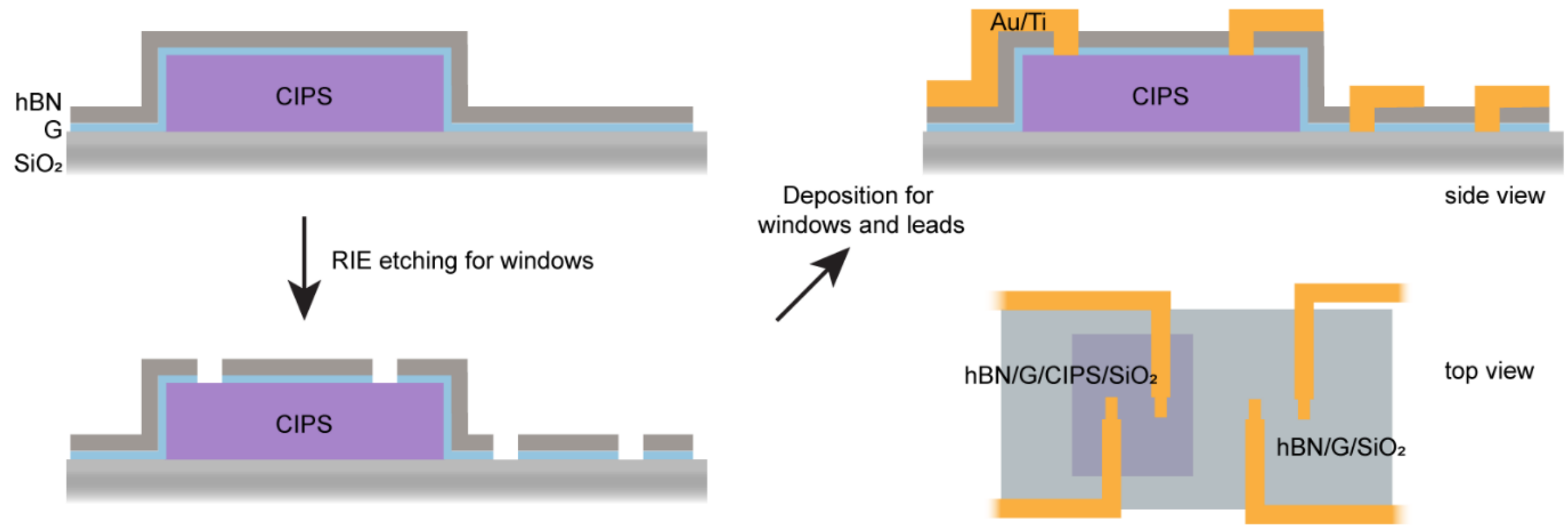

Figure S4. Schematic of the fabrication of hBN/graphene/CIPS devices. A hBN/Graphene flake is placed on a CIPS/SiO_2/Si stack (top left), then reactive ion etching (RIE) through the top hBN opens windows in the encapsulation layer (bottom left). Subsequent Ti/Au deposition defines the contact electrodes and window regions (top right, side view; bottom right, top view).

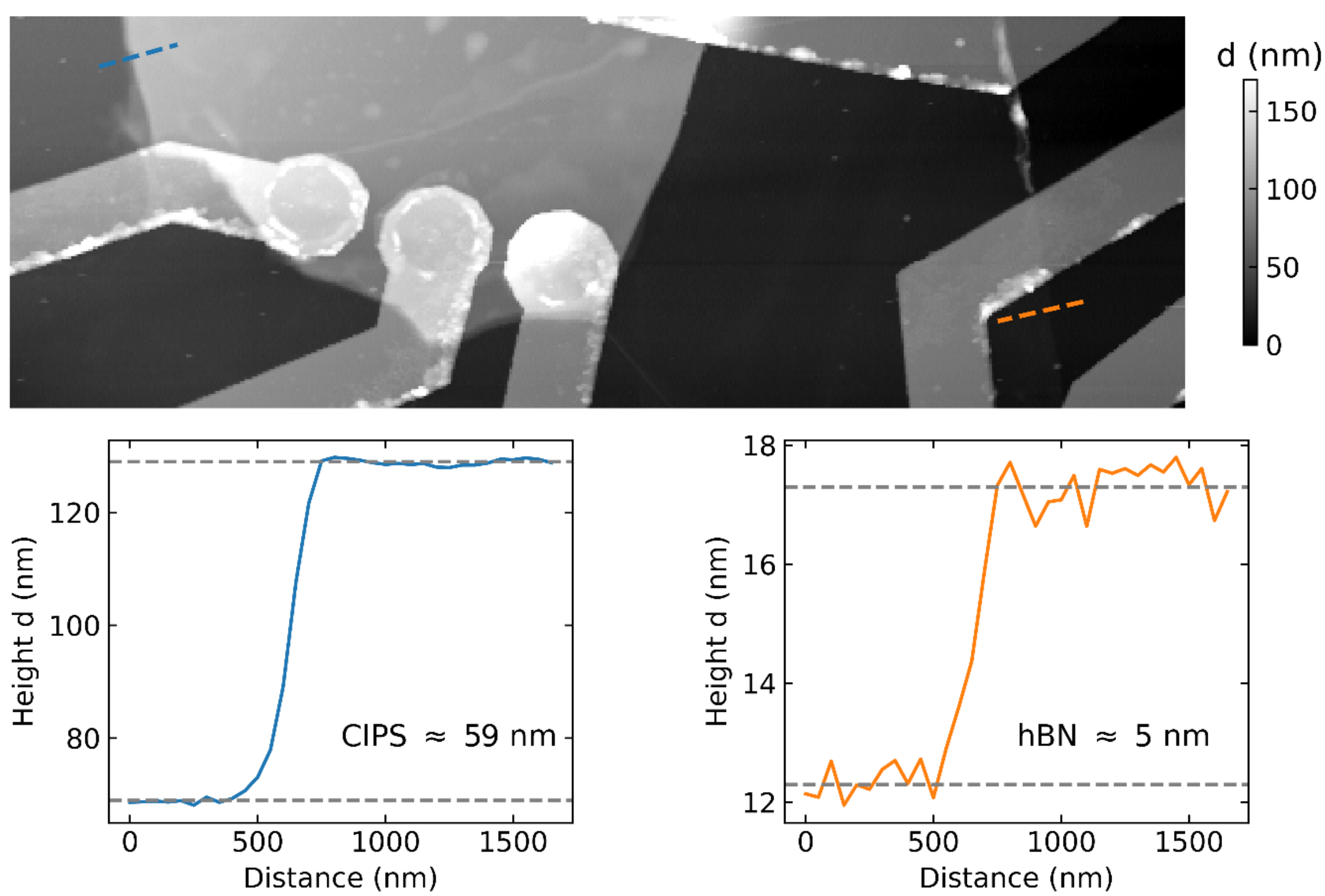

Figure S5. (Top) Topography image of hBN/graphene/CIPS/SiO_2/Si device shown in Fig. 1 of the main text. The field of view corresponds to the white dashed box in Fig. S3f. (Bottom) Height profiles of CIPS (blue) and hBN (orange) extracted along the blue and orange dashed lines in the topography image, respectively.

IV. Additional infrared Nano-imaging maps

Additional near-field imaging and dispersion analysis for graphene on thick CIPS are shown in Figs. S6-S8.

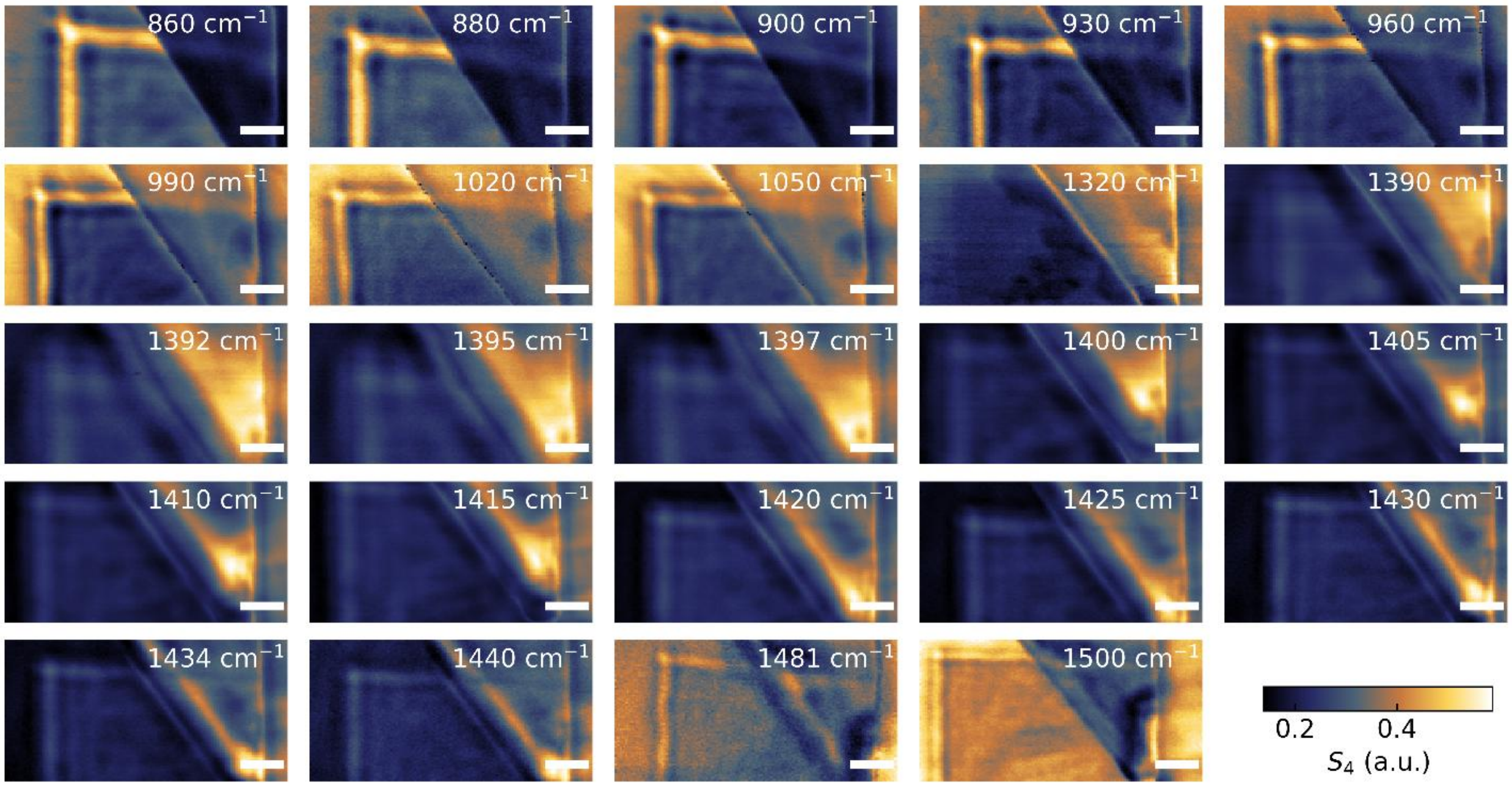

Figure S6. Frequency-dependent near-field amplitude (S_4) maps for hBN/Graphene/CIPS/SiO_2/Si for 170 nm thick CIPS. Scale bars in all panels are 100 nm.

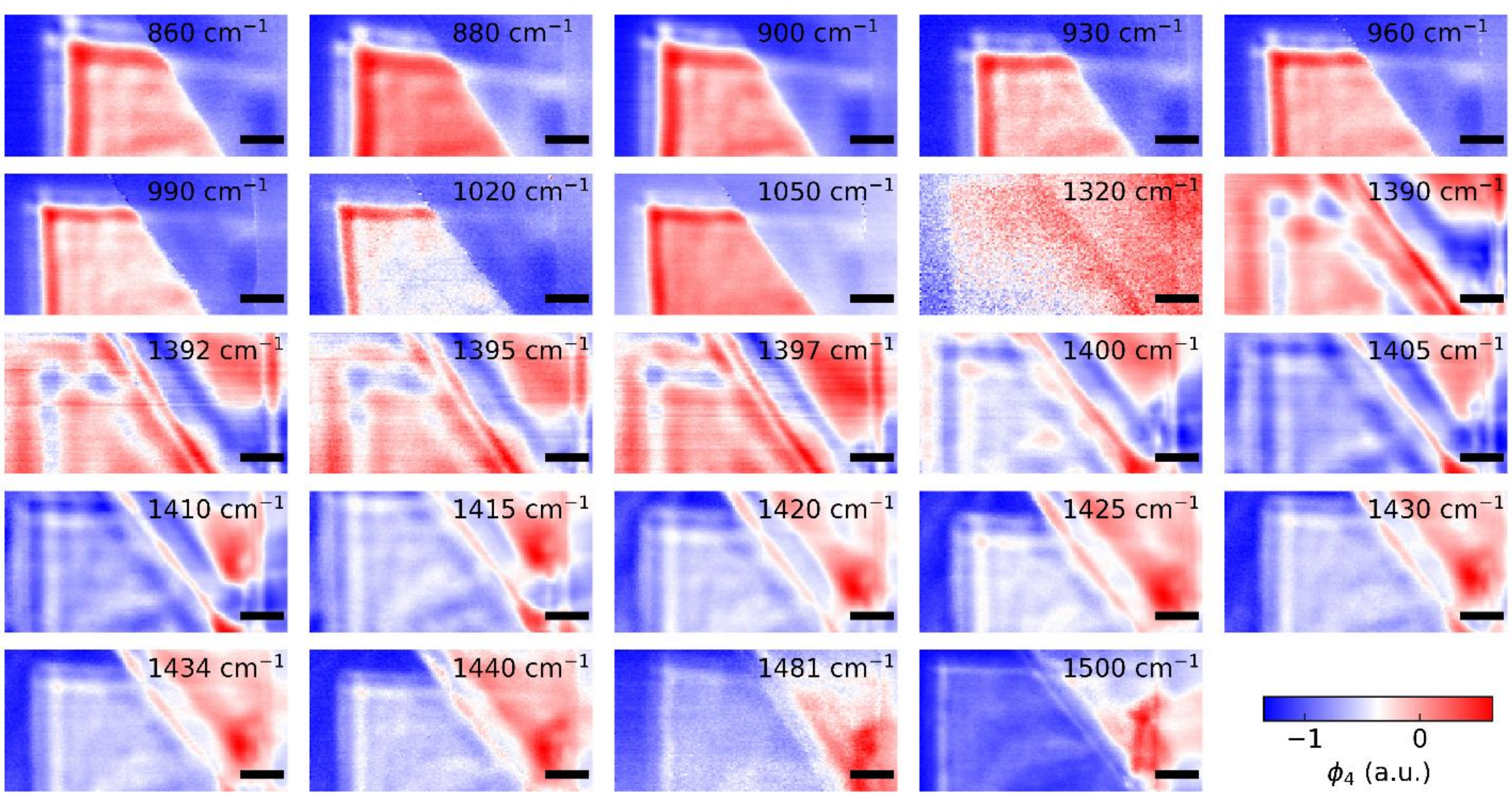

Figure S7. Frequency-dependent near-field phase (ϕ_4) maps for hBN/Graphene/CIPS/SiO_2/Si for 170 nm thick CIPS. Scale bars in all panels are 100 nm.

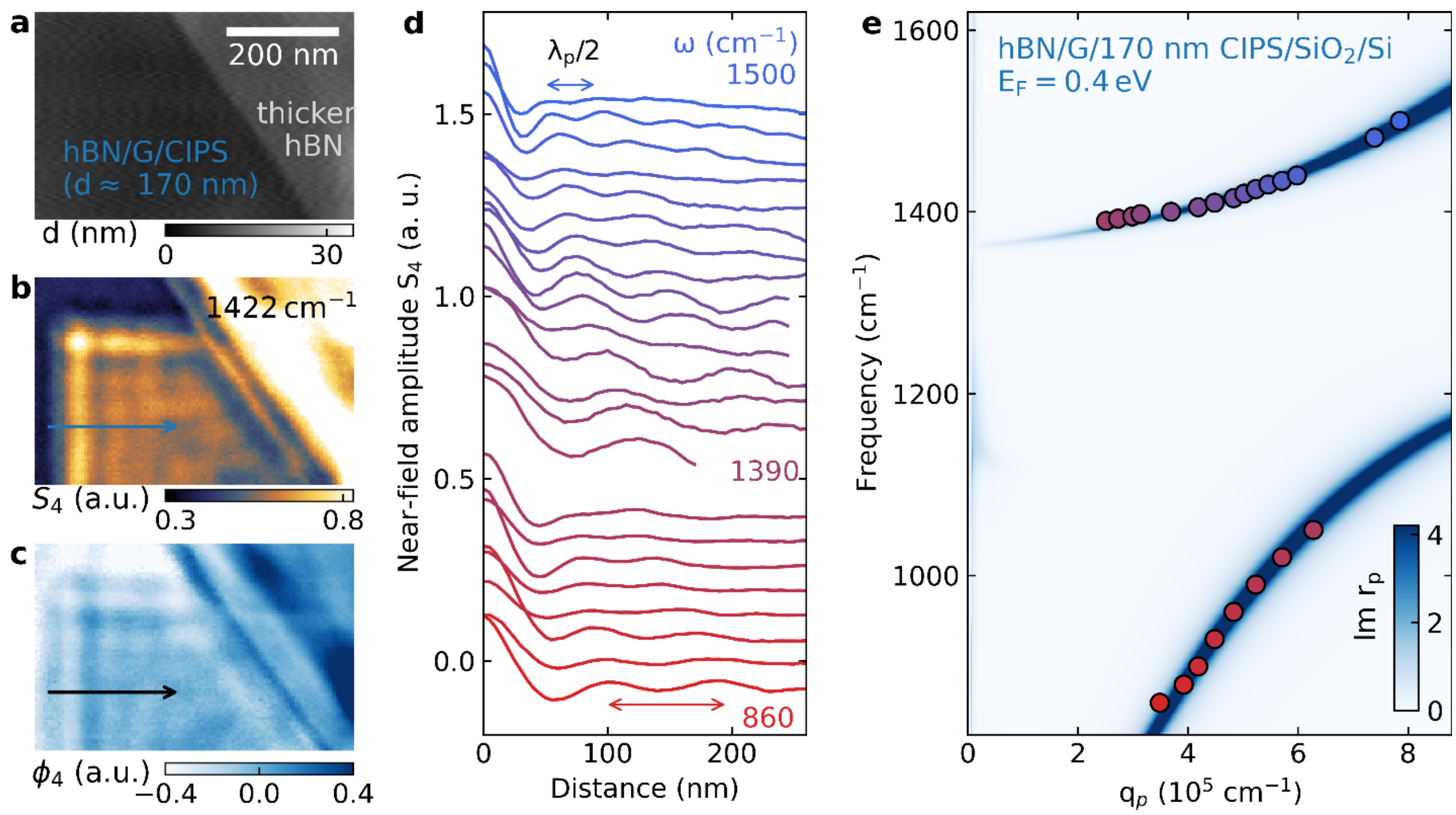

Figure S8. Charge-transfer plasmons in graphene on thick CIPS. **a**, Topography of hBN/graphene on thick CIPS ($d \approx 170$ nm). Top right corner indicates a step in the hBN. The near-field amplitude (S_4) and phase (ϕ_4) maps at laser frequency of $\omega = 1422$ cm^{-1} for the hBN/G/CIPS (170 nm) sample are shown in **b** and **c**. The amplitude and phase maps show plasmonic fringes with the same periodicity. Scale bar indicates 200 nm. **d**, Line profiles of the averaged S_4 in **b** as a function of distance from the left graphene edge, showing a systematic change in polariton wavelength with laser frequency ($\omega = 860 - 1500$ cm^{-1}). Line profiles are shifted vertically for clarity. **e**, Extracted polariton momenta (colored dots) $q_p = 2\pi/\lambda_p$ from **d**, overlaid on the theoretical model of the imaginary part of the reflection coefficient Im r_p for the hBN/G/CIPS heterostructure. The Fermi energy of graphene used in the model is $E_F = 0.4$ eV.

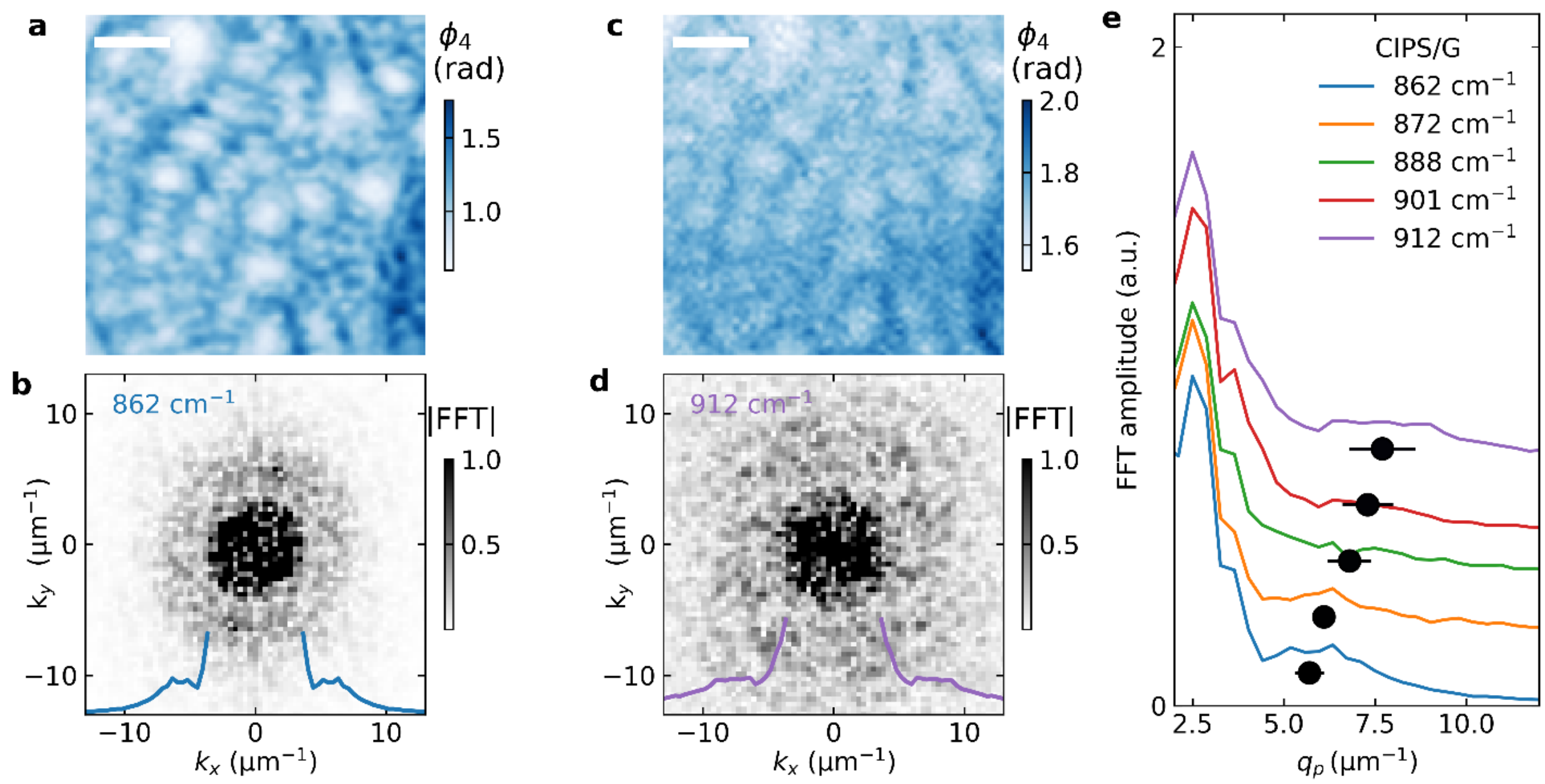

Figure S9. Near-field phase (ϕ_4) of the 26 nm CIPS/Graphene sample at $\omega = 862\ \mathrm{cm}^{-1}$ (**a**) and at $\omega = 912\ \mathrm{cm}^{-1}$ (**c**), showing short-wavelength inhomogeneities that change with laser frequency, characteristic of plasmonic origin. The fast Fourier transform (FFT) amplitude of phase maps in **(a)** and **(c)** are shown in **(b)** and **(d)**, respectively. The radially averaged line profile (blue and purple lines) shows a weak peak indicative of the plasmon polaritons. **e**, The extracted polariton momenta ($q_p = 2\pi k_p$) as a function of laser frequencies (black dots), overlaid on the radially averaged FFT amplitude profiles (solid lines) for $\omega = 862\ \mathrm{cm}^{-1}$ to $\omega = 912\ \mathrm{cm}^{-1}$.

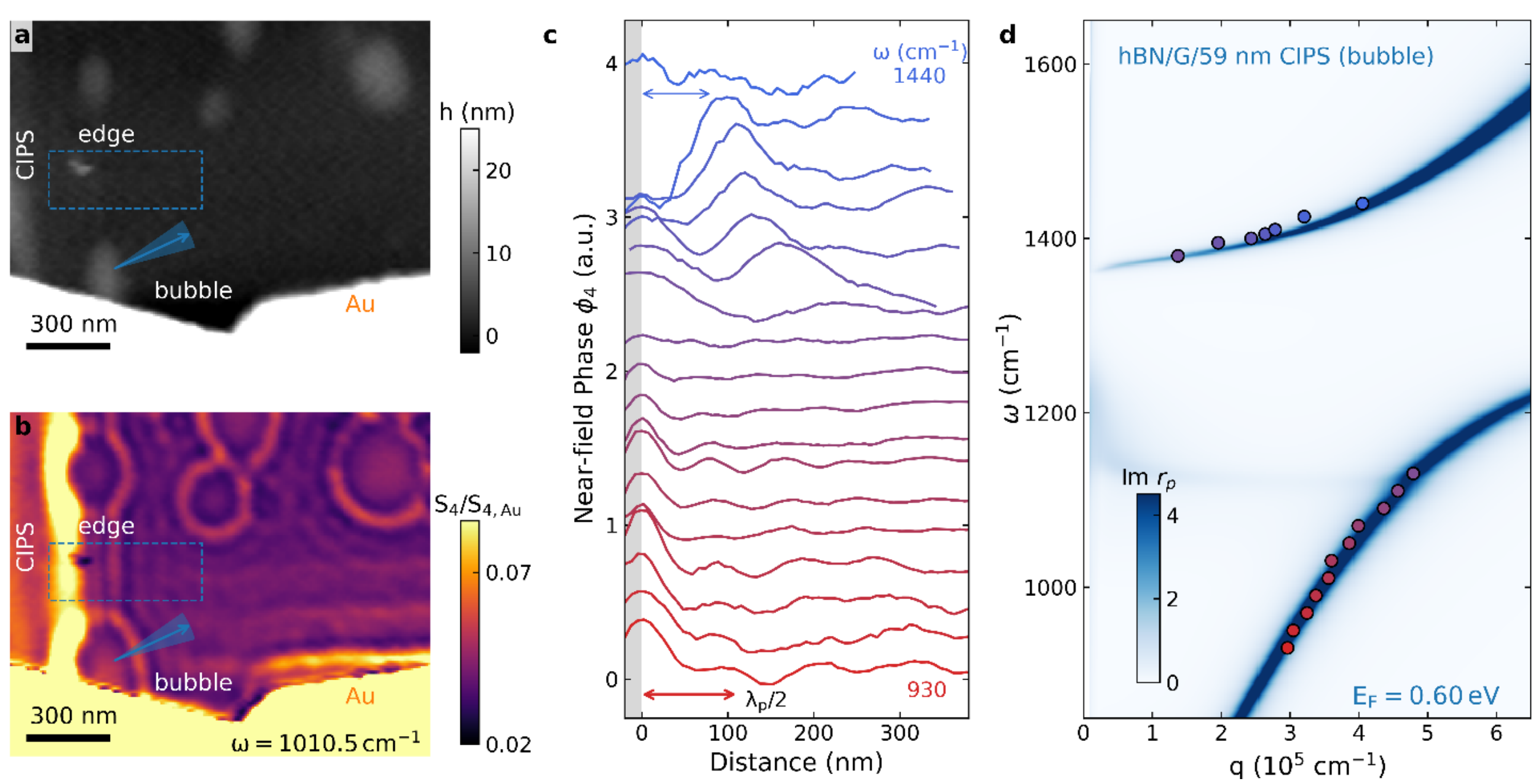

Figure S10. Bubble-reflected graphene plasmon dispersion in hBN/graphene/59 nm CIPS. **a,** AFM topography of the hBN/graphene/59 nm CIPS device near the CIPS edge and Au contact,

showing local bubbles that act as nanoscale plasmon reflectors. **b,** Au-normalized near-field amplitude image, $S_4/S_{4,\mathrm{Au}}$, acquired at $\omega = 1010.5\ \mathrm{cm}^{-1}$ in the same region as **a**. Clear plasmon interference fringes are observed around the bubble and device edges. **c,** Near-field phase line profiles, ϕ_4, taken from the bubble region over laser frequencies from 930 to 1440 cm^{-1}. The fringe periodicity changes with increasing frequency and corresponds to $\lambda_p/2$, consistent with tip-launched plasmons reflected by the bubble. **d,** Extracted plasmon momenta q_p overlaid on the calculated imaginary part of the *p*-polarized reflection coefficient, $\mathrm{Im}\, r_p$, for the hBN/graphene/CIPS heterostructure. The dispersion is well described by a graphene Fermi energy of $E_F = 0.60$ eV, close to the value extracted from the edge-reflected plasmon (blue dashed rectangles) dispersion of the same device in Fig. 2c of the main text, $E_F = 0.63$ eV.

V. Second Harmonic Generation Mapping and Raman Spectra of exfoliated $CuInP_2S_6$ flakes

The SHG mapping measurements were performed using a Solstice Ace Ti: Sapphire laser (800 nm, 100 fs pulse width, 80 MHz repetition rate) incident normally on the sample through a 0.75 NA objective. The reflected SHG signal was collected using a photomultiplier tube (PMT). The sample was mounted with the crystallographic *(000l)* plane normal facing the incoming beam. The raw polarization-dependent SHG response was first measured in a rotating-polarizer (0–360°) and fixed-analyzer (0°, 45°, 90°, and 135° relative to the laboratory X-axis) geometry (Figure S11). The data from Fig. S11a geometry were simultaneously fitted in the rotating polarizer fixed analyzer geometry (only two orthogonal analyzer positions) using SHAARP.ml package[6].

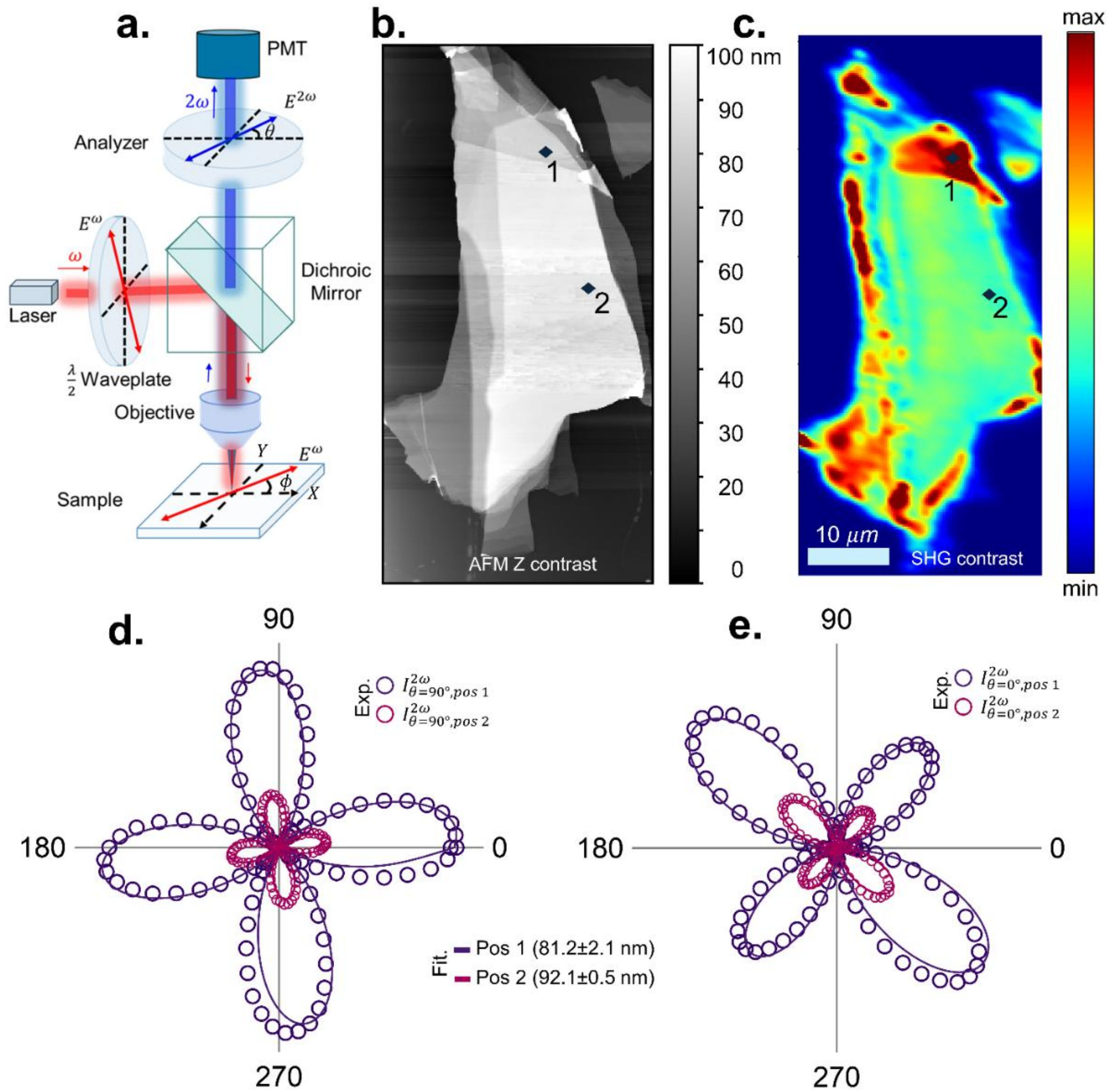

Figure S11. **a,** Schematic illustration of the second-harmonic generation (SHG) experimental geometry (rotating polarizer and fixed analyzer) used in this study. **b,** Atomic force microscopy (AFM) height/contrast image of the CIPS flake. **c,** SHG microscopy map of the same flake measured under normal-incidence geometry; scale bar: 10 μm. **d–e,** SHG polarimetry in the rotating-polarizer/fixed-analyzer configuration for two orthogonal analyzer geometries, respectively.

$CuInP_2S_6$ crystallizes in the monoclinic *m* point group in the ferroelectric phase. In second-harmonic generation (SHG), the incident electric field E^{ω} at frequency ω induces a nonlinear polarization $P^{2\omega}$ at frequency 2ω within a noncentrosymmetric medium. This interaction is described by the relation ($P_i^{2\omega} = d_{ijk}E_j^{\omega}E_k^{\omega}$) where the indices (*i, j, k*) correspond to the Cartesian polarization components, and d_{ijk} represents the elements of the second-order nonlinear susceptibility tensor. SHG mapping measurements on the exfoliated $CuInP_2S_6$ flakes (Fig. S11b) were carried out using a fundamental excitation at 800 nm, producing a second-harmonic output

at 400 nm. The SHG tensor for the point group *m* is represented in Voigt notation. The expression is given by:

$$\begin{pmatrix} d_{11} & d_{12} & d_{13} & 0 & d_{15} & 0 \\ 0 & 0 & 0 & d_{24} & 0 & d_{26} \\ d_{31} & d_{32} & d_{33} & 0 & d_{35} & 0 \end{pmatrix}$$

Here, $d_{11}, d_{12}, d_{13}, d_{15}, d_{24}, d_{26}, d_{31}, d_{32}, d_{33}$, and d_{35} are non-zero SHG coefficients. The SHG polarimetry measurement was performed by rotating the polarization of incident light while having a fixed analyzer configuration.

In the normal incidence reflection geometry (Fig. S11a), we analyze the rotating-polarizer/fixed-analyzer configuration. At the experimental geometry (sample normal || crystallographic (*000l*) plane normal) and analyzer ||X and analyzer||Y, the SHG intensities are given by,

$$I_x^{2\omega} = f_{11} d_{11} \cos(2\theta) + f_{22} d_{22} \sin(2\theta)$$

$$I_Y^{2\omega} = f_{33} d_{26} \cos(\theta) \sin(\theta)$$

Here, f_{11}, f_{22} and f_{33} are the Fresnel coefficients of the sample. Normal-incidence reflection measurements under the two experimental geometries were fitted using these equations.

The normal-incidence reflection measurements under the two experimental geometries were simultaneously fitted, as shown in Fig. S11d-e. The excellent agreement between the experiment and simulation confirms the homogeneity of the material.

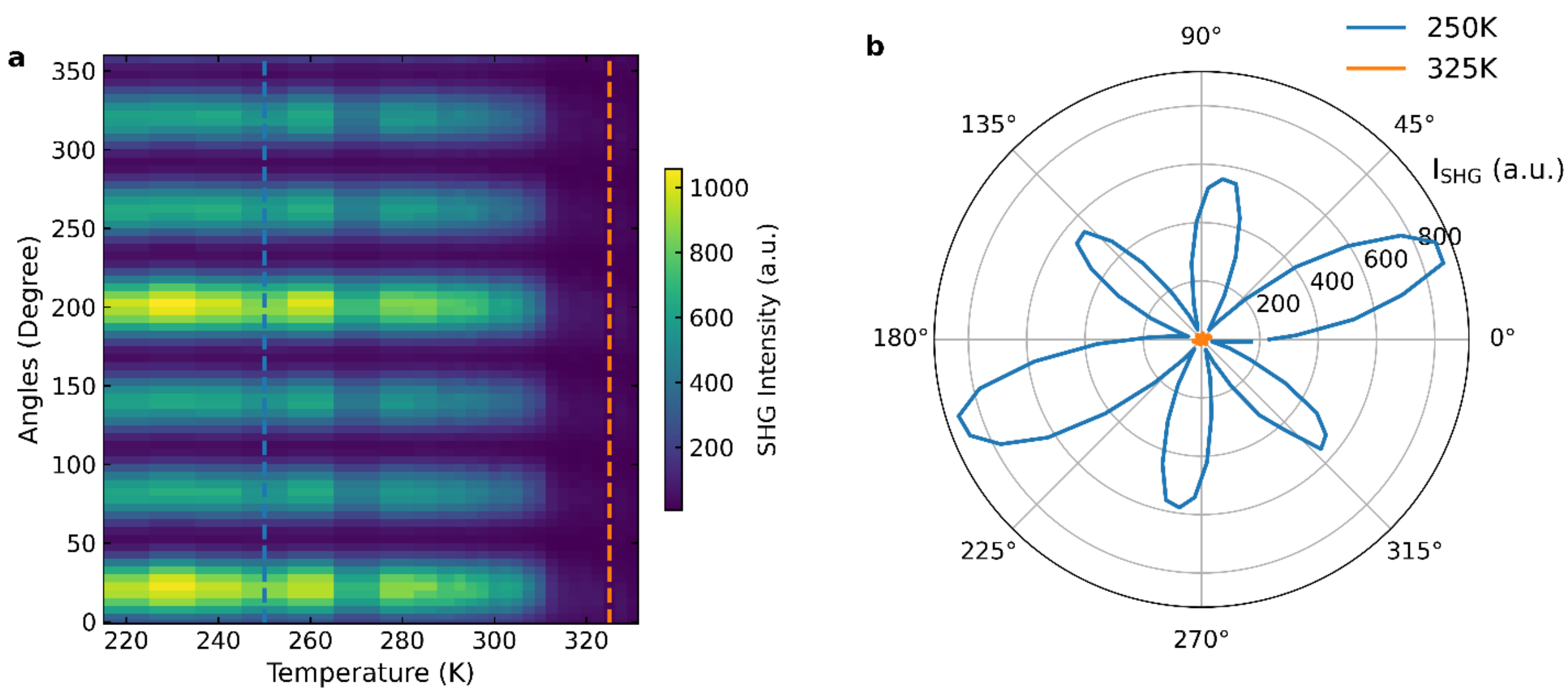

Figure S12. Second Harmonic Generation (SHG) of thick $CuInP_2S_6$. **a,** Temperature- and angle-dependent SHG intensity of thick CIPS flakes exfoliated on SiO_2/Si substrates. The SHG intensities are strongly suppressed above the ferroelectric transition temperature ($T_C \approx 315$ K). **b,** Polar plot of the SHG intensity as a function of the polarization angle of the incident light at below (250 K, blue) and above (325 K, orange) the T_C. The angular pattern represents the effects of strain

and the symmetry of the shifted Cu and In atoms responsible for the broken inversion symmetry, consistent with previous reports[7,8].

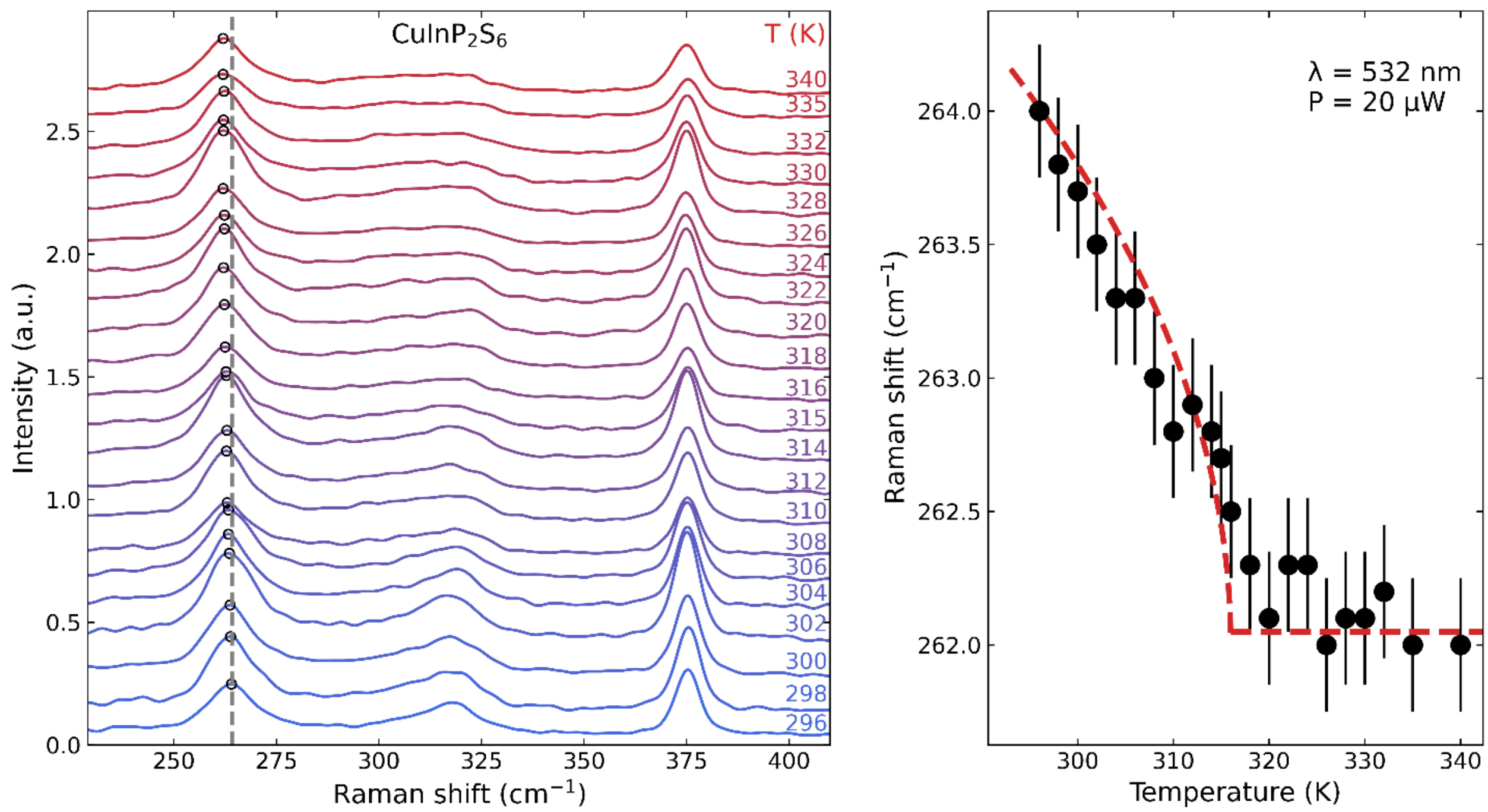

Figure S13. Temperature-dependent Raman spectra of thick $CuInP_2S_6$. (Left) Raman spectra of a thick CIPS flake (~40 nm) from T = 296 K to 340 K, showing the temperature evolution of three representative Raman phonons in CIPS (264 cm^{-1}, 315 cm^{-1}, 375 cm^{-1} at 296 K). Black circles highlight an A_g phonon that gradually softens from ∼264 cm^{-1} at room temperature to ∼262 cm^{-1} at 340 K, consistent with previous reports[9–11]. (Right) Phonon frequency of the A_g mode (black dots) as a function of temperature. The red dashed line is a guide to the eye, indicating the ferroelectric transition temperature T_C near 315 K.

VI. Correlation between CIPS polarization domains and graphene doping

To provide additional evidence that opposite CIPS polarizations modulate graphene doping, we performed domain-resolved PFM and near-field optical measurements on an hBN/graphene/CIPS heterostructure, as shown in Fig. S14. These measurements compare opposite polarization domains within the same graphene sheet and on the same CIPS flake, thereby minimizing sample-to-sample variation.

In Fig. S14a, AFM topography identifies the hBN/graphene/CIPS region, with the dashed purple lines marking the graphene-covered area. The PFM phase image in Fig. S14b reveals two main CIPS domains within this region, labeled $P\downarrow$ and $P\uparrow$, corresponding to opposite out-of-plane polarization orientations. The corresponding near-field optical phase image ϕ_3 in Fig. S14c shows domain-dependent contrast in the graphene-covered area. Line profiles extracted across the

domain boundary, shown in Figs. S14d, S14e, further show that the $P\downarrow$ domain produces a larger near-field phase shift, indicating higher graphene doping, whereas the $P\uparrow$ domain produces a weaker phase shift and lower doping. These spatially correlated PFM and near-field measurements support polarization-dependent charge transfer at the graphene/CIPS interface.

We note that many small polarization bubbles with lateral sizes below 100 nm are also present in the PFM image. These small features make quantitative extraction of local doping at every bubble unreliable. However, they do not produce resolvable near-field contrast in the bare CIPS region or in the weakly doped $P\uparrow$ graphene-covered region. In contrast, small polarization bubbles in the more strongly doped $P\downarrow$ graphene-covered region scatter propagating plasmons, further supporting the assignment of the enhanced near-field contrast to higher graphene doping in this domain. Thus, the domain-resolved measurements provide device-level evidence that local CIPS polarization modulates graphene doping and the resulting FACT plasmon response.

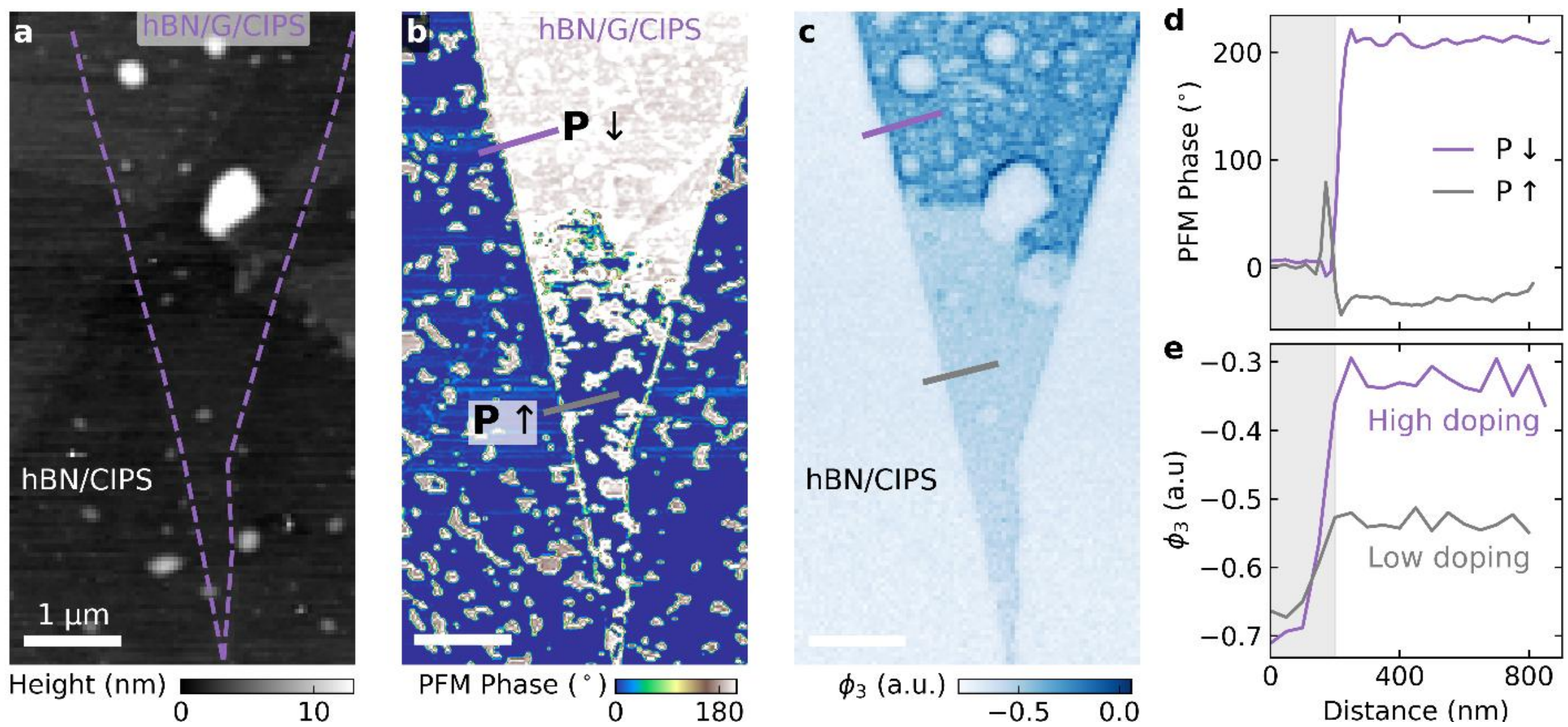

Figure S14. Correlation between CIPS polarization domains and graphene doping. **a,** AFM topography of a hBN/graphene/CIPS heterostructure region. The top hBN covers the entire CIPS region. The dashed purple lines mark the graphene-covered area. **b,** PFM phase image showing two main CIPS domains inside the graphene-covered region with opposite out-of-plane polarizations, labeled $P\downarrow$ and $P\uparrow$. **c,** Near-field optical phase ϕ_3 measured over the same region, showing varying phase contrast across the graphene-covered area relative to the surrounding CIPS. **d,e,** Line profiles taken along the colored lines in **b,c**. The $P\downarrow$ domain shows a larger near-field phase shift, indicating higher graphene doping, whereas the $P\uparrow$ domain shows weaker doping. These spatially correlated PFM and near-field measurements support polarization-dependent charge transfer at the graphene/CIPS interface. Scale bars, 1 µm.

The domain-dependent charge-transfer contrast is further supported by DFT calculations for the opposite CIPS polarization direction compared to Figs. 1b,1c of the main text, which predict negligible charge-transfer doping in graphene (Fig. S15).

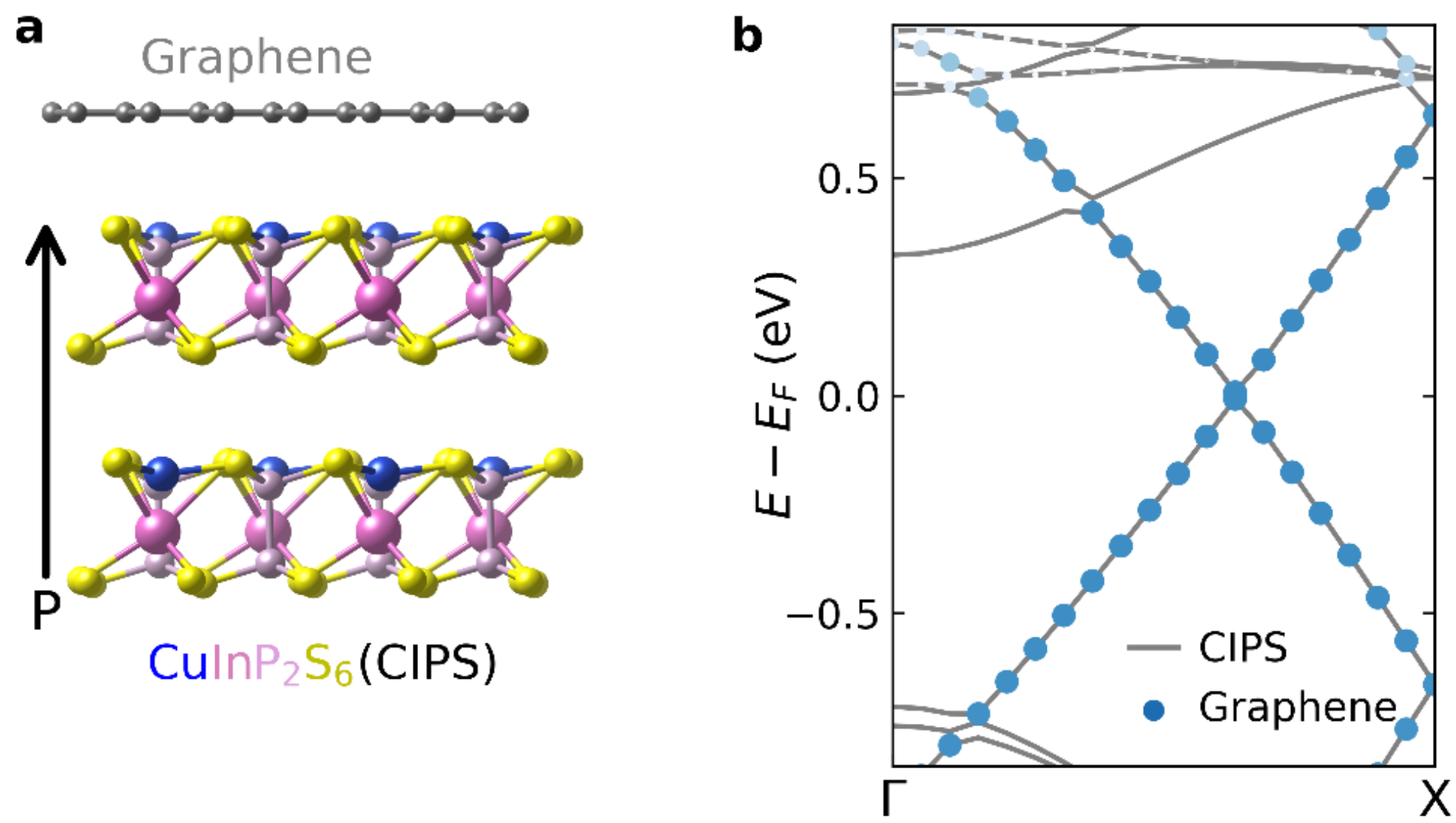

Figure S15. Absence of charge-transfer doping in graphene/bilayer $CuInP_2S_6$ for the opposite polarization direction. **a**, Side view of the lattice structure of graphene (gray) on bilayer CIPS, with a polarization direction pointing towards graphene (black arrow), opposite to the polarization direction in Fig. 1b. **b**, Calculated electronic band structure of graphene/2L-CIPS structure shown in **a**. The graphene Dirac bands (blue) show negligible doping, in contrast to Fig. 1c in the main text. The Dirac-point shifts in graphene induced by opposite CIPS polarizations are consistent with prior calculations[12]. Similar polarization-direction-dependent charge transfer has also been predicted in graphene/In_2Se_3 heterostructures[13].

VII. Electrostatic model for ferroelectric-assisted charge transfer in G/CIPS

The charge-transfer process in graphene/CIPS can be viewed as a ferroelectric extension of ordinary work-function-mediated charge transfer in graphene heterostructures. In conventional charge-transfer graphene systems, the carrier density is determined by the work-function mismatch, graphene band filling, and the electrostatic voltage drop across the interfacial spacer. In graphene/CIPS, the out-of-plane polarization of CIPS provides an additional electrostatic contribution.

The microscopic pathway is illustrated schematically as follows. The thickness-dependent net out-of-plane polarization of CIPS produces bound surface charge at the CIPS surfaces,

$$\sigma_b(d) = \hat{\boldsymbol{n}} \cdot \mathbf{P}_{\text{eff}}(d) \simeq \pm P_{\text{eff}}(d). \qquad \text{(S1)}$$

Here $P_{\text{eff}}(d)$ denotes the effective interfacial polarization sensed by graphene. It can be smaller than the bulk CIPS polarization in ultrathin flakes because finite screening and depolarization-field

effects suppress the net out-of-plane polarization. The bound surface charge modifies the graphene/CIPS interfacial potential and induces an image charge in graphene. Because graphene has a finite density of states and is separated from CIPS by a van der Waals gap, this induced charge does not perfectly compensate the polarization charge. Instead, the graphene carrier density is determined self-consistently by graphene band filling, the graphene/CIPS gap capacitance, and the residual depolarization field in CIPS.

This continuum picture is consistent with recent treatments of vdW ferroelectric interfaces[14], where interfacial charge redistribution and dipole formation are treated quantitatively from first principles. In our system, DFT calculations explicitly capture the atomistic graphene/CIPS interface, including the relaxed graphene/CIPS separation and charge transfer in ultrathin CIPS, as shown in Figs. 1b and 1c of the main text and Fig. S15. The calculated $|E_F| \approx 0.2$ eV for graphene on bilayer CIPS provides a microscopic reference for the baseline interfacial charge transfer. For thicker CIPS, we describe the additional ferroelectric-induced charge transfer using the electrostatic model below.

Following the work-function-mediated charge-transfer model of Kim et al.[15], the conventional charge-transfer balance can be written as

$$\hbar v_F \sqrt{\pi n} + \frac{en}{C_{\mathrm{gap}}} = \Delta\Phi_{\mathrm{WF}}, \qquad \text{(S2)}$$

where n is the graphene carrier density, $E_F = \hbar v_F \sqrt{\pi n}$, and $\Delta\Phi_{\mathrm{WF}} = \Phi - \Phi_G$ is the work-function mismatch between graphene, Φ_G, and the active layer, Φ. The areal gap capacitance is $C_{\mathrm{gap}} = \varepsilon_0 \varepsilon_{\mathrm{gap}} / t_{\mathrm{gap}}$, where $t_{\mathrm{gap}}$is the graphene/CIPS van der Waals gap. We use $t_{\mathrm{gap}} = 0.34$nm, corresponding to the DFT-relaxed vertical separation between the graphene carbon plane and the top sulfur plane of CIPS.

In graphene/CIPS, the total graphene carrier density can be separated into a baseline work-function contribution and a ferroelectric-induced contribution,

$$n(d) = n_0 + n_{\mathrm{FE}}(d), \qquad \text{(S3)}$$

where n_0 is the baseline charge-transfer density in the ultrathin-CIPS limit and $n_{\mathrm{FE}} = n - n_0$ is the additional ferroelectric-induced carrier density. The baseline density is determined from the ultrathin-limit Fermi energy, $E_{F,0} = 0.18$ eV, which gives $n_0 = 2.4 \times 10^{12}$ cm^{-2}.

The polarization-induced graphene charge only partially compensates the CIPS bound charge. The residual uncompensated polarization charge is

$$\sigma_{\mathrm{res}}(d) = P_{\mathrm{eff}}(d) - e[n(d) - n_0]. \qquad \text{(S4)}$$

In a monodomain dielectric approximation, this residual sheet charge produces an average depolarization field in CIPS,

$$E_{\text{dep}}(d) = \frac{P_{\text{eff}}(d) - e(n - n_0)}{\varepsilon_0 \varepsilon_b}, \qquad \text{(S5)}$$

where ε_b is the static background dielectric constant of CIPS ($\varepsilon_b = 7$ from Ref.[16]). Equivalently, the depolarization voltage can be written using the CIPS depolarization capacitance per unit area,

$$C_{\text{dep}}(d) = \frac{\varepsilon_0 \varepsilon_b}{d}. \qquad \text{(S6)}$$

The resulting charge-transfer balance is

$$\hbar v_F \sqrt{\pi n} + \frac{en}{C_{\text{gap}}} = \Delta\Phi_{\text{WF}} + \frac{P_{\text{eff}}(d) - e(n - n_0)}{C_{\text{dep}}(d)}. \qquad \text{(S7)}$$

The left side is the graphene band-filling cost plus the voltage drop across the graphene/CIPS gap. The right side contains the baseline work-function drive and the depolarization-field drive caused by the residual polarization charge. Setting $P_{\text{eff}} = 0$ reduces Eq. S7 to the ordinary work-function charge-transfer model.

Subtracting the baseline equation at $n = n_0$ gives a more transparent finite-screening form,

$$\hbar v_F \left(\sqrt{\pi n} - \sqrt{\pi n_0}\right) + \frac{e(n - n_0)}{C_{\text{gap}}} = \frac{P_{\text{eff}}(d) - e(n - n_0)}{C_{\text{dep}}(d)}. \qquad \text{(S8)}$$

This expression shows how graphene screening and the CIPS depolarization field determine the final carrier density. Adding charge to graphene screens the polarization charge and lowers the CIPS depolarization energy, but it costs graphene band-filling energy and gap-charging energy. The equilibrium density is reached when these contributions balance.

To model the thickness dependence, we use the PFM amplitude as an experimental indicator of the thickness-dependent out-of-plane ferroelectric response. The rising branch of the PFM data below ~ 100 nm is fit using

$$A_{\text{PFM}}(d) = A_0 + A_1 f(d), \qquad \text{(S9)}$$

with $f(d) = 0$ below a critical thickness d_c, and $f(d) = \left(\frac{d - d_c}{d}\right)^{\beta}, d > d_c$.

The fit gives $d_c = 13.5$ nm and $\beta = 1.1$, as shown in Fig. S16a. We then write

$$P_{\text{eff}}(d) = P_{\text{bulk}}^{\text{model}} f(d), \qquad \text{(S10)}$$

where $P_{\text{bulk}}^{\text{model}}$ is an effective polarization scale for the net interfacial polarization that enters the electrostatic model.

Using the PFM-derived $P_{\text{eff}}(d)$ as the input, Eq. S7 reproduces the observed increase of graphene $|E_F|$ in the single-domain thickness range, as shown in Fig. S16b. For the representative model curve in Fig. S16b (green dashed line), we use an effective polarization scale $P_{\text{bulk}}^{\text{model}} \approx$

$5.3\ \mu C/cm^2$, close to the commonly reported room-temperature polarization scale of CIPS. The resulting quantitative comparison is consistent with electrostatic compensation of the CIPS polarization charge. For example, at $d = 59$ nm, the measured $E_F = 0.61$ eV gives a graphene carrier density of $n = 2.7 \times 10^{13}\ cm^{-2}$. After subtracting the baseline density $n_0 = 2.4 \times 10^{12}\ cm^{-2}$, the ferroelectric-induced charge density is $e(n - n_0) = 4.0\ \mu C/cm^2$. This value is essentially equal to the effective $P_{eff}(59\ nm) = 4.01\ \mu C/cm^2$ used in the representative model curve, indicating that graphene nearly compensates the available interfacial polarization charge near the maximum-doping thickness.

Overall, this electrostatic model provides a microscopic-to-continuum pathway from CIPS polarization to graphene doping. CIPS polarization produces bound charge, the bound charge induces graphene image charge, and the residual uncompensated charge generates a depolarization field. The final graphene carrier density is set by the self-consistent balance between depolarization-field screening, graphene band filling, and vdW-gap charging. The observed graphene doping is quantitatively close to the expected polarization-charge scale, while its thickness dependence follows the finite-size recovery of the out-of-plane CIPS polarization.

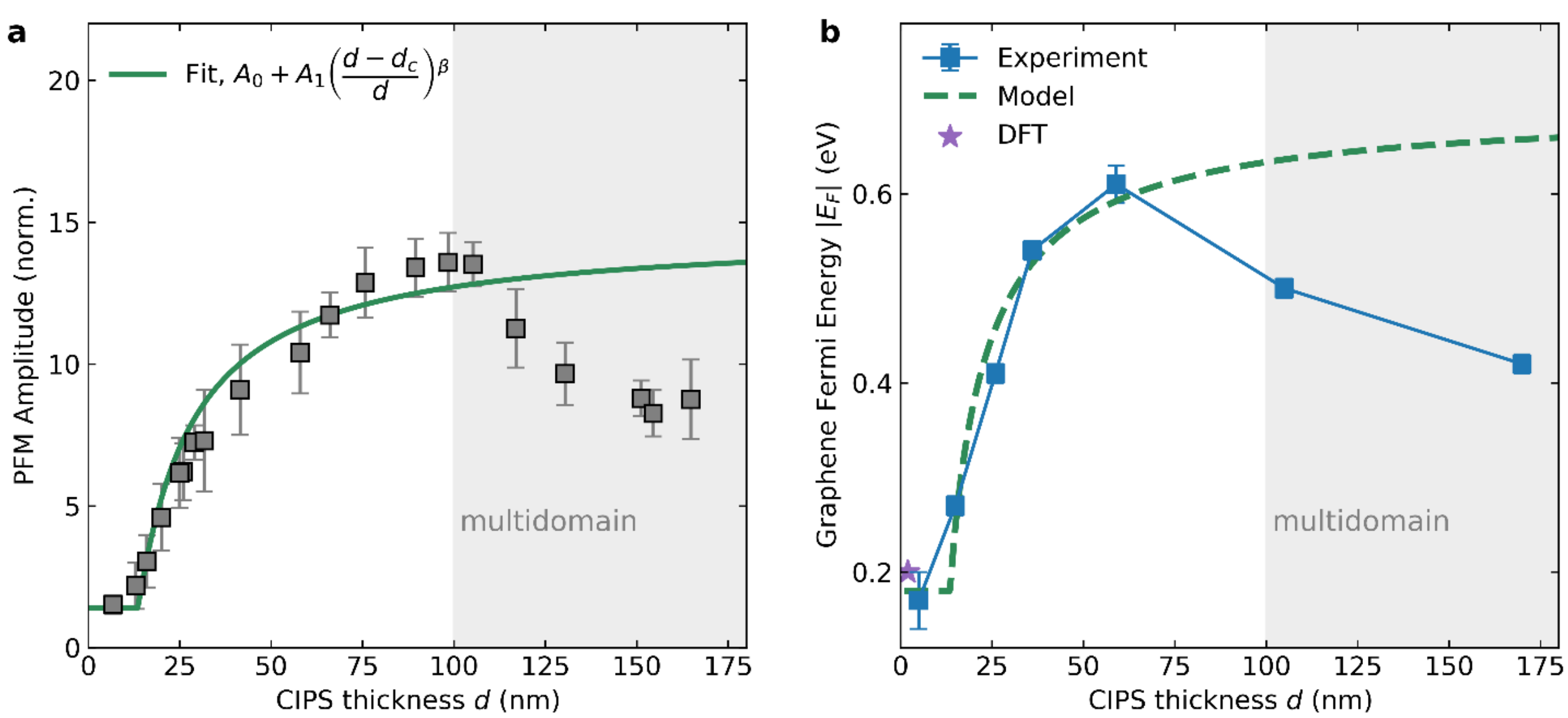

Figure S16. Thickness-dependent PFM response and electrostatic charge-transfer model. **a,** Normalized PFM amplitude as a function of CIPS thickness. The green curve is a fit to the rising PFM branch below 100 nm using $A_{PFM}(d) = A_0 + A_1 f(d)$, where $f(d) = 0$ for $d \leq d_c$ and $f(d) = ((d - d_c)/d)^\beta$ for $d > d_c$. The fit gives $d_c = 13.5$ nm and $\beta = 1.1$. The gray shaded region marks the thickness range where multidomain or mixed-polarization textures become important, and the single-domain fit is no longer valid. **b,** Graphene Fermi energy (blue squares) extracted from plasmon dispersion as a function of CIPS thickness. The green dashed curve is the electrostatic charge-transfer model using $P_{eff}(d) = P_{bulk}^{model} f(d)$ as the thickness-dependent polarization input. The purple star shows the DFT-calculated Fermi energy for graphene on bilayer

CIPS. The model captures the rise of $|E_F|$ in the single-domain thickness range (<100 nm), while the reduced $|E_F|$ in thicker CIPS is consistent with domain averaging that lowers the net interfacial polarization charge sensed by graphene.

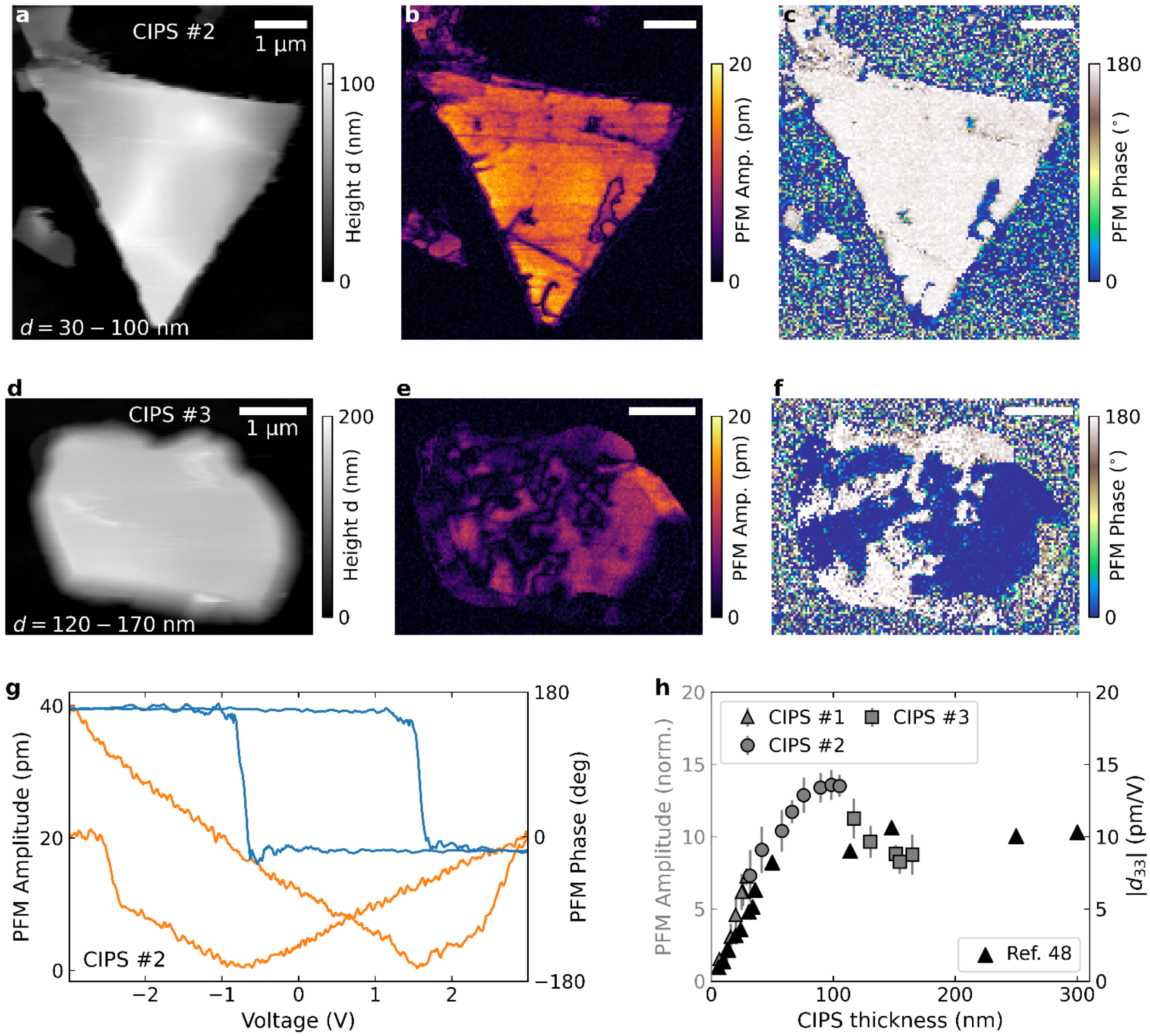

Figure S17. Summary of PFM measurements on $CuInP_2S_6$ flakes with varying thickness. **a**, Topography image of the CIPS #2 sample on a SiO_2/Si substrate with CIPS thickness ranging from ~30 to ~100 nm. PFM amplitude image (**b**) shows a larger piezoresponse in the thicker triangular-shaped flake than the thinner surrounding flakes. **c**, Corresponding PFM phase image, indicating that a single domain dominates the ferroelectric polarization direction for the ~100-nm-thick flake. **d-f**, Topography (**d**), PFM amplitude (**e**) and PFM phase **(f)** images of the thicker CIPS #3 flake (~120 – 170 nm), revealing greater domain variations. Scale bars are all 1 μm. **g**, Bias sweep of the PFM amplitude (orange) and phase (blue) for the ~100-nm-thick region of CIPS #2, showing expected switching behavior. **h**, Thickness dependence of the normalized PFM amplitude obtained from CIPS #1, #2, and #3 (gray symbols) and the thickness dependence of the piezoelectric coefficient from Ref.[17] (black triangles).

VIII. FACT plasmons in nanoscale structures and long-term stability

We further examined FACT plasmon behavior near local nanoscale structures in graphene/CIPS heterostructures. As shown in Fig. S18, FACT plasmons remain visible near a nanoscale bubble, a bilayer graphene domain wall, and a graphene/CIPS edge. These local structures mainly modify the propagation geometry of FACT plasmons rather than eliminating the plasmonic response. The nanoscale bubble, with a lateral size of ~100 nm, locally perturbs interfacial charge transfer and scatters plasmons into a ring-like interference pattern (Fig. S18b). The bilayer graphene domain wall is not resolved in the AFM topography, but produces narrow near-field contrast and acts as a line-like plasmon reflector (Fig. S18d). Near the graphene/CIPS edge, the sharp boundary between graphene-covered CIPS and bare CIPS supports edge-confined plasmons[18] that propagate along the graphene edge (Fig. S18f). These measurements show that FACT plasmons remain sensitive to local nanoscale structures while preserving clear propagating plasmon signatures.

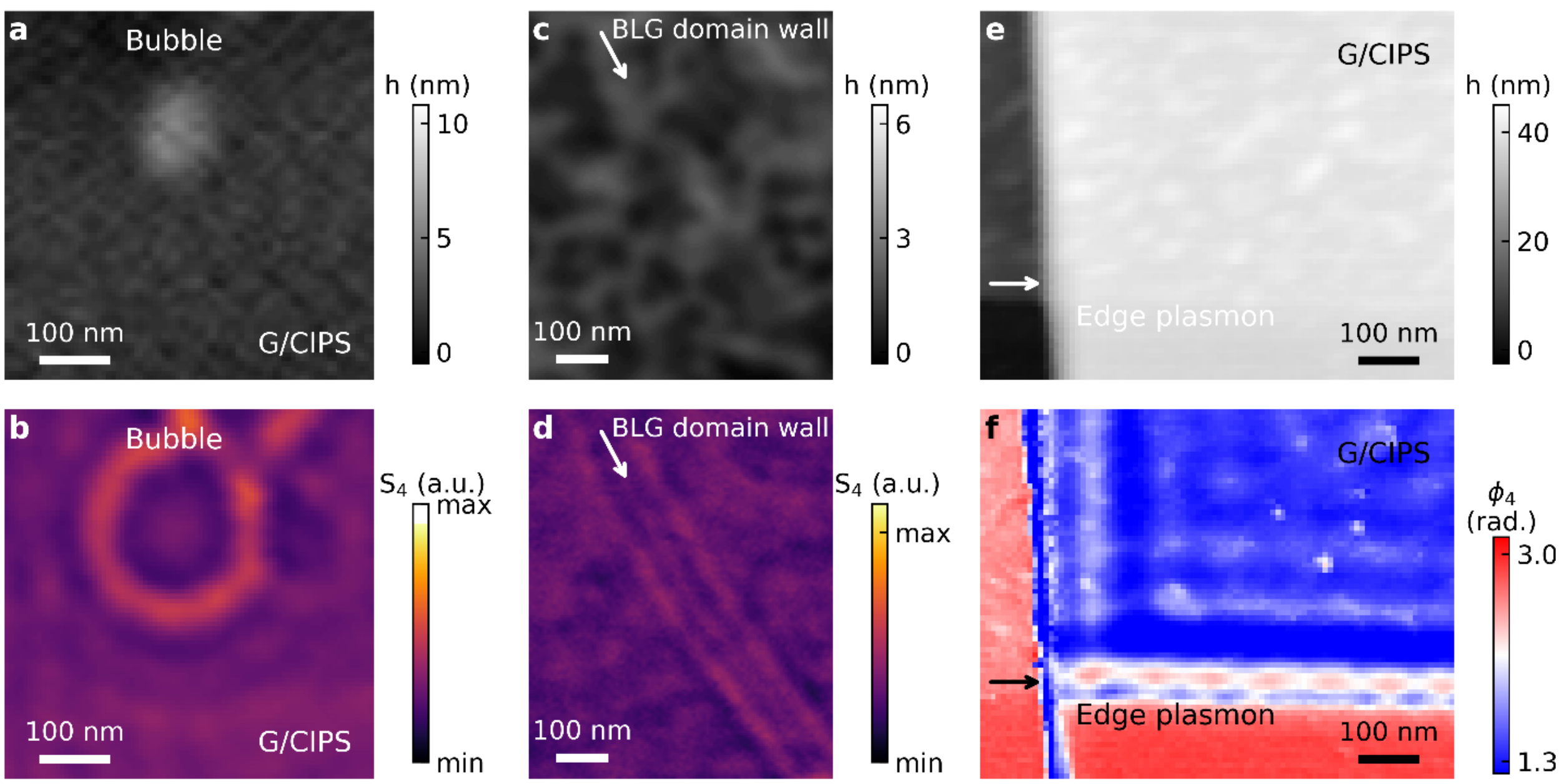

Figure S18. FACT plasmon response near local nanoscale structures. **a,b,** AFM topography and near-field amplitude S_4 around a nanoscale bubble in graphene/CIPS. The bubble, ~100 nm in lateral size, locally perturbs the charge transfer at the interface and scatters FACT plasmons, producing a ring-like near-field fringe pattern. **c,d,** Topography and near-field amplitude S_4 image near a bilayer graphene (BLG) domain wall. The atomic-scale BLG domain wall is not resolved in the AFM topography (white arrow), but it acts as a sharp line scatterer that reflects FACT plasmons. **e,f,** Topography and near-field phase ϕ_4 near a sharp graphene/CIPS edge. In addition to plasmons propagating in the interior of graphene, the sharp boundary between graphene-covered CIPS and bare CIPS also supports edge-confined plasmons that propagate along the graphene edge (black arrow).

We also investigated whether FACT plasmon behavior extends beyond monolayer graphene by measuring hBN/bilayer graphene/CIPS heterostructures. Prior near-field studies of bilayer graphene showed that tensile and shear domain-wall solitons produce distinct plasmon reflection profiles[19], with characteristic fringe spacings of $\lambda_p/2$ and $3\lambda_p/4$, respectively. In our hBN/bilayer graphene/CIPS device, near-field amplitude images acquired at 900 cm^{-1} show clear plasmonic fringes near both the graphene edge and internal line-like features assigned to bilayer graphene domain walls (Fig. S19a). The edge profile shows a fringe spacing of approximately $\lambda_p/2$, consistent with conventional edge-reflected graphene plasmons. In contrast, the two domain-wall profiles show different interference patterns: DW 1 exhibits peak spacing close to $3\lambda_p/4$, while DW 2 shows spacing close to $\lambda_p/2$ (Fig. S19b). These profiles are consistent with the established near-field signatures of different bilayer graphene domain-wall solitons.

To further verify the plasmonic origin of the domain-wall contrast, we measured frequency-dependent line profiles across DW 1 from 860 to 1050 cm^{-1}. The interference peaks shift systematically with infrared frequency, as expected for dispersive plasmon polaritons rather than static topographic or electrostatic contrast (Fig. S19c). The extracted plasmon momenta agree well with the calculated hBN/bilayer graphene/CIPS plasmon dispersion using E_F=0.3 eV (Fig. S19d). This agreement indicates that charge transfer from CIPS also dopes bilayer graphene sufficiently to support propagating plasmons without an applied gate voltage.

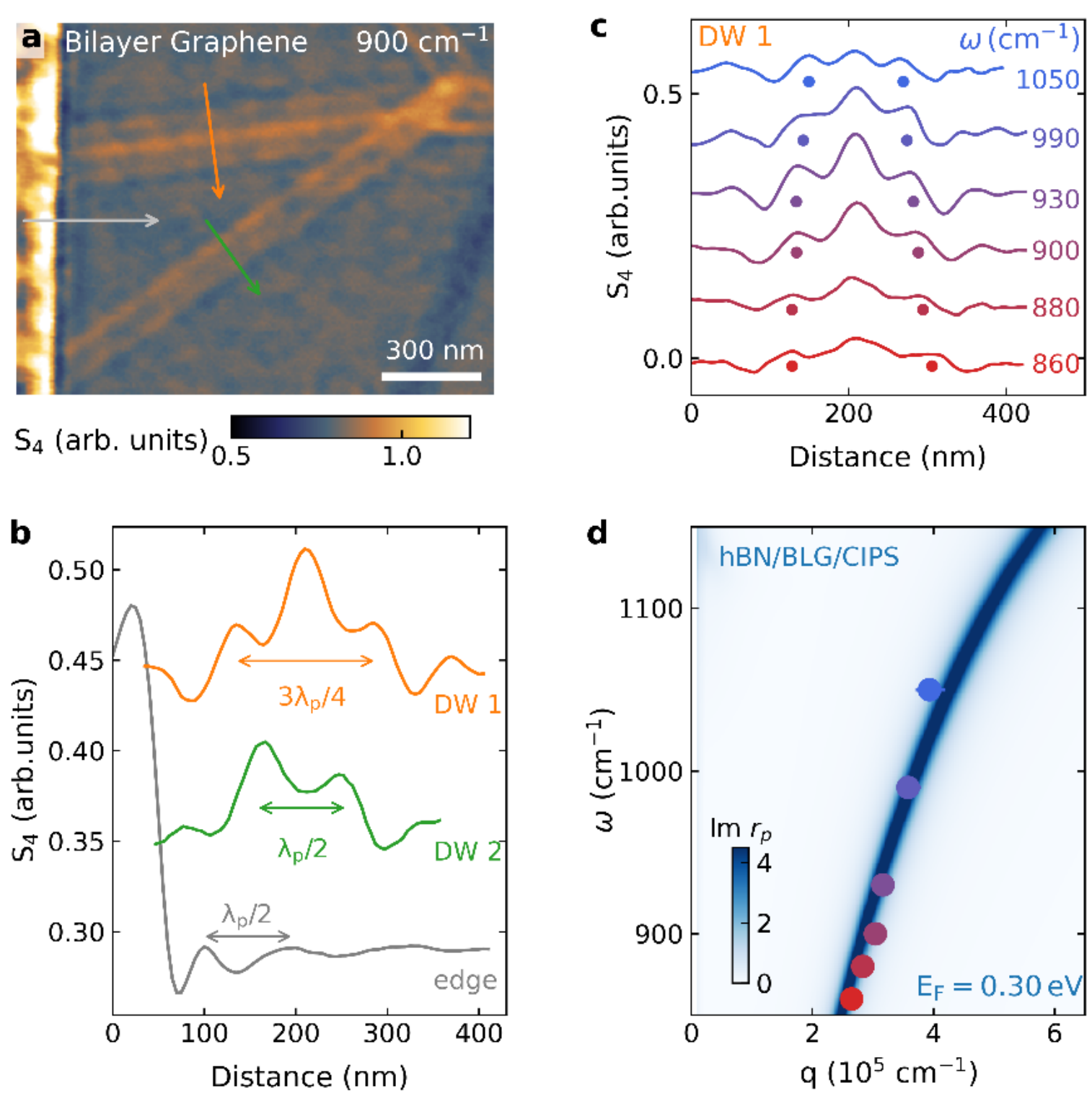

Figure S19. Domain-wall plasmons in bilayer graphene on CIPS. Different types of domain walls (tensile vs shear) in bilayer graphene are characterized by different fringe spacings ($\lambda_p/2$ vs $3\lambda_p/4$), as demonstrated in Ref. [19]. **a**, Near-field amplitude image S_4 of the hBN/bilayer graphene

(BLG)/CIPS heterostructure at 900 cm^{-1}. Colored arrows mark representative linecuts across the edge and domain-wall regions. **b**, Line profiles extracted from panel **a**. The edge profile shows a fringe spacing of approximately $\lambda_p/2$, while domain-wall profiles DW 1 and DW 2 show spacing consistent with $3\lambda_p/4$ and $\lambda_p/2$, respectively. **c**, Near-field line profiles across DW 1 measured at different infrared frequencies, with extracted peak positions indicated by colored dots. **d**, Plasmon dispersion extracted from the DW 1 fringe spacing, compared with the calculated hBN/BLG/CIPS plasmon dispersion (Im r_p) for $E_F = 0.3$ eV.

The plasmonic response of few-layer graphene beyond bilayer graphene is expected to be more complicated than that of monolayer graphene. Recent near-field infrared measurements on trilayer graphene[20] showed that Bernal (ABA) and rhombohedral (ABC) stacking support distinct plasmonic responses, with different plasmon wavelengths and strong plasmon reflection at ABA/ABC lateral junctions. These results indicate that, although FACT plasmons should persist in thicker few-layer graphene on CIPS, their doping response and plasmon dispersion are expected to be further complicated by stacking order, internal domain structure, and stacking-dependent optical conductivity.

Finally, we examined the long-term stability of the FACT plasmon response in a completed hBN/graphene/CIPS device. Figure S20 compares near-field phase images of the same device region measured on Nov. 19, 2025 and May 24, 2026. The overall graphene phase contrast and the edge-reflected plasmon fringe remain visible after ~6 months. Line profiles extracted across the graphene/CIPS edge show similar fringe spacing in the two measurements (Fig. S20c), indicating that the plasmon wavelength λ_p, and therefore the charge-transfer-induced graphene doping level, remains stable over this time period. These results show that the FACT plasmon response is retained in completed hBN/graphene/CIPS heterostructures over extended time.

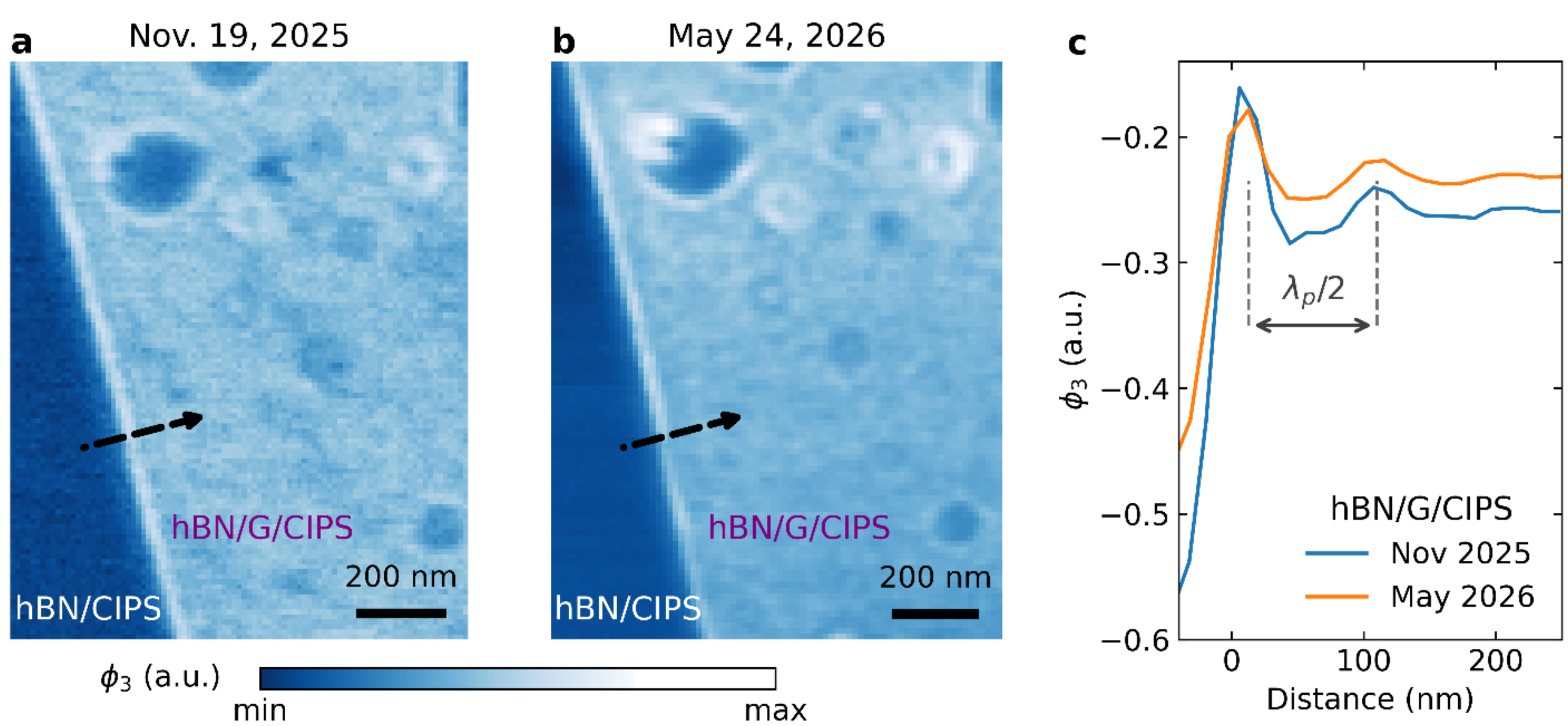

Figure S20. Long-term stability of the FACT plasmon response. **a,b,** Near-field phase ϕ_3 images of the same hBN/graphene/CIPS device region measured on Nov. 19, 2025 and May 24, 2026, respectively. The graphene/CIPS edge and the associated edge-reflected plasmon fringe remain clearly visible after ~6 months. Dashed arrows indicate the positions used to extract the line profiles. **c,** Line profiles of ϕ_3 across the graphene/CIPS edge for the two measurements. Similar fringe spacing indicates that the plasmon wavelength (λ_p), and therefore the charge-transfer-induced graphene doping level, remains stable over more than 6 months. These results provide device-level evidence that the FACT plasmon response is retained in completed hBN/graphene/CIPS heterostructures over extended time.

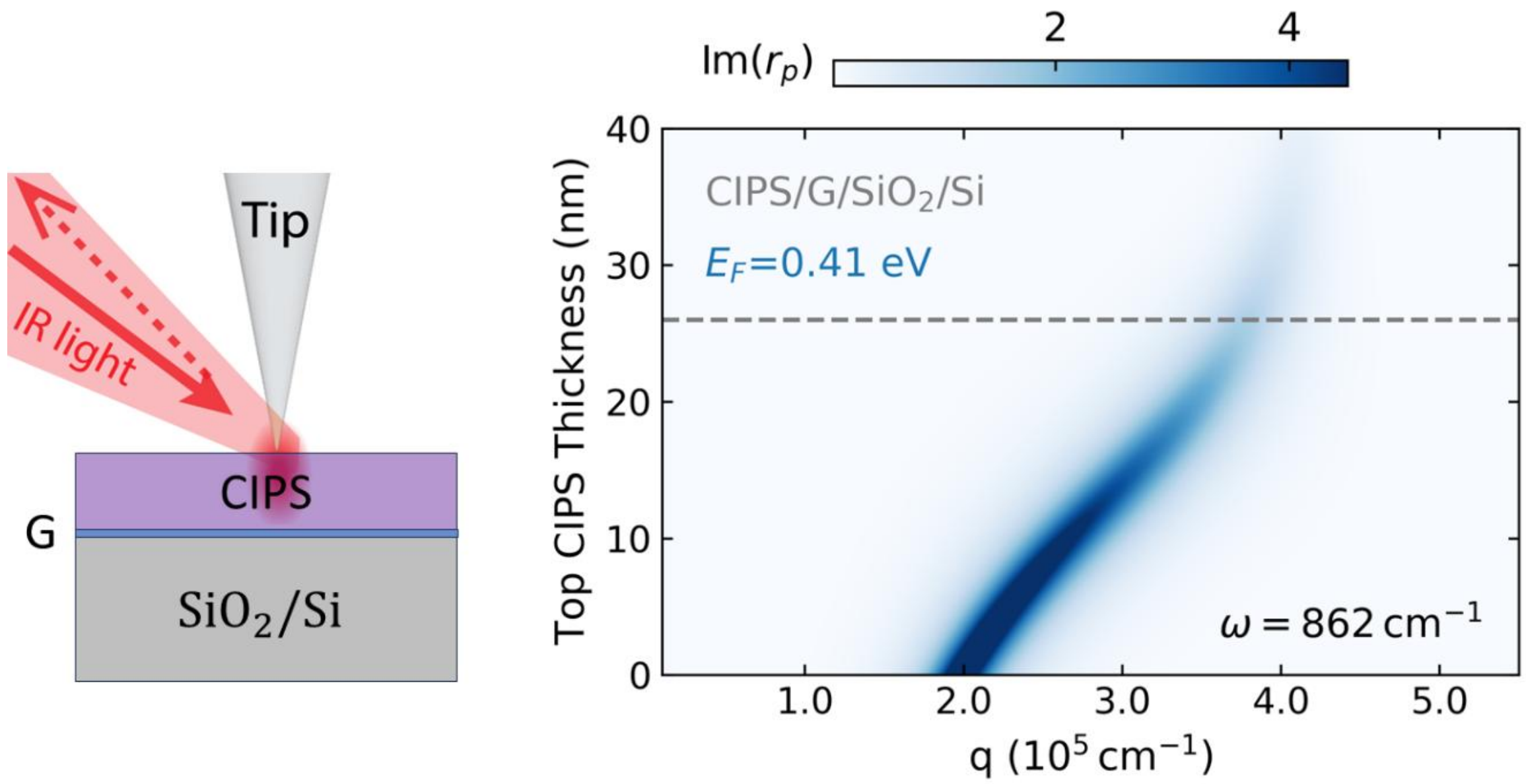

Figure S21. Impact of the top CIPS layer thickness on the near-field optical contrast. (Left) Schematic of the near-field measurements on CIPS/graphene/SiO_2/Si structure. An infrared laser (IR, red arrow) is focused onto the metallic tip (silver), which is raster-scanned across the sample. (Right) Calculated imaginary part of the reflection coefficient (Im r_p) for the CIPS/graphene/SiO_2/Si structure as a function of the top CIPS layer thickness. At fixed graphene Fermi energy ($E_F = 0.41$ eV) and frequency ($\omega = 862$ cm^{-1}), the polariton momentum q increases with increasing CIPS thickness. However, as the top CIPS thickness increases (gray dashed line marks 26 nm), the overall near-field contrast diminishes since the charge-transfer interface is buried beneath CIPS.

IX. Characterization of monolayer graphene

We confirmed the monolayer nature of the graphene used in the main-text measurements by AFM, near-field optical imaging, and Raman spectroscopy, as shown in Fig. S22. Figure S22a shows an

AFM topography image near the folded hBN boundary shown in Fig. 2d of the main text, where adjacent hBN/graphene/CIPS and hBN/CIPS regions can be identified on the same 5 nm CIPS flake. The height profile taken across the graphene edge, marked by the dashed orange line in Fig. S22a, shows a step height of approximately 0.4 nm (Fig. S22b), consistent with monolayer graphene.

Near-field imaging of the same region further confirms the presence of graphene. The near-field phase image in Fig. S22c shows clear contrast only in the hBN/graphene/CIPS region, whereas the neighboring hBN/CIPS region shows no comparable graphene-related response. In addition, Raman spectra taken from the same graphene flake on SiO_2, corresponding to the gray points in Figs. 2f and 2g of the main text, show a G peak at 1584.5 cm^{-1} and a 2D peak at 2680.9 cm^{-1}, with an intensity ratio $I_{2D}/I_G = 5.97$ (Fig. S22d). The large 2D-to-G intensity ratio and the measured phonon frequencies are characteristic of high-quality, weakly doped monolayer graphene, confirming that the graphene used in the hBN/graphene/CIPS device is monolayer[21].

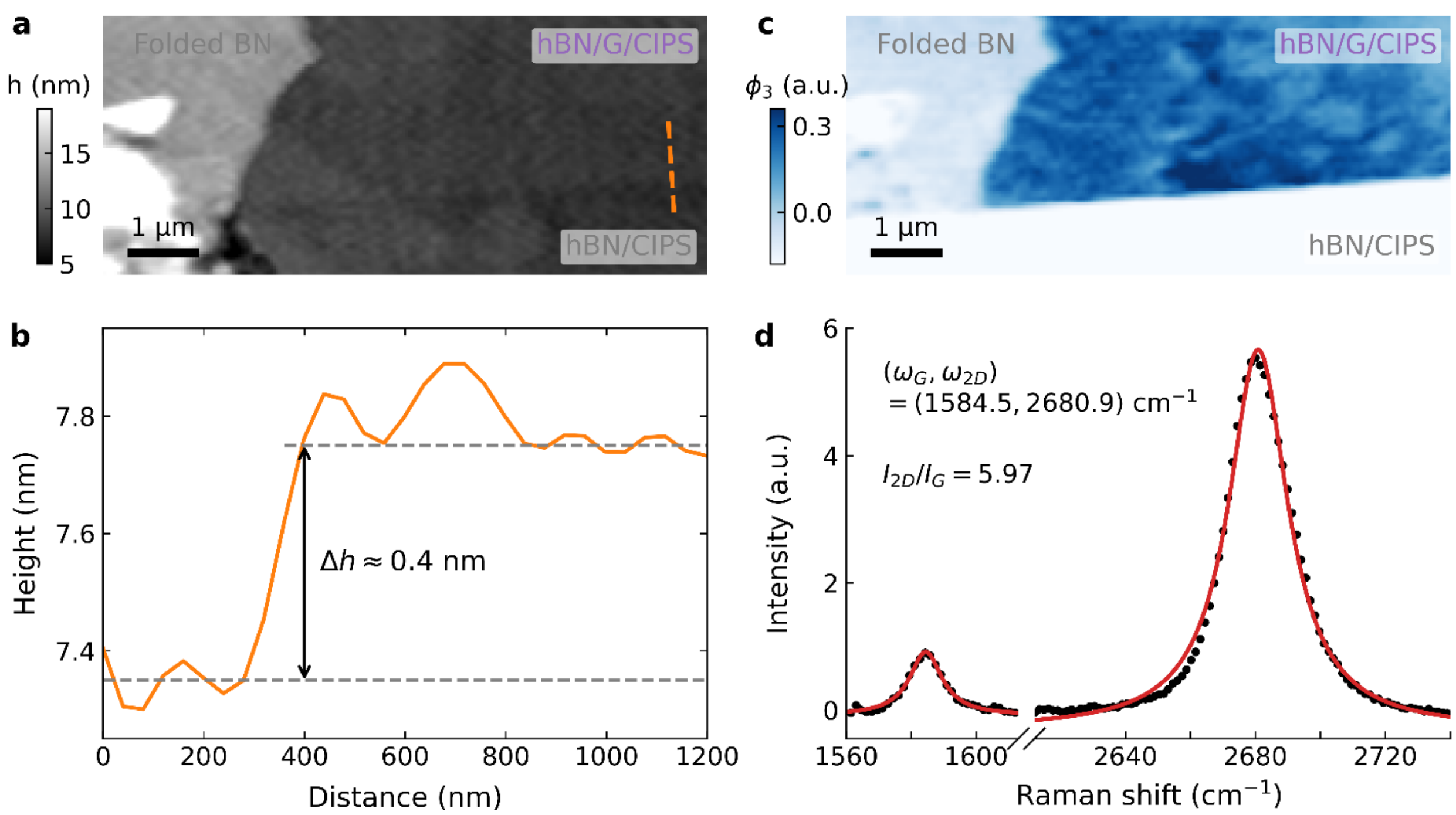

Figure S22. Verification of monolayer graphene in the hBN/G/CIPS (5 nm) sample in main text Fig. 2. **a**, AFM topography of the folded-hBN boundary and adjacent hBN/G/CIPS and hBN/CIPS regions. The dashed orange line marks the linecut location across the graphene edge. **b**, AFM height profile across the dashed line in **a**, showing a step height of approximately 0.4 nm, consistent with monolayer graphene. **c**, Near-field phase image (ϕ_3) of the same region in **a**, showing clear contrast when graphene is present. **d**, Raman spectrum from the same graphene flake (gray dot in Fig. 2f). The fitted G and 2D peak positions are consistent with undoped graphene, with intensity ratio I_{2D}/I_G = 5.97, further confirming the monolayer nature of graphene[21].

References

1. Studenyak, I. P. *et al.* Disordering effect on optical absorption processes in CuInP2S6 layered ferrielectrics. *phys. stat. sol. (b)* **236**, 678–686 (2003).
2. Zhang, Y. *et al.* Low-force pulse switching of ferroelectric polarization enabled by imprint field. *Nat. Commun.* **16**, 5271 (2025).
3. Xu, X. *et al.* Towards compact phase-matched and waveguided nonlinear optics in atomically layered semiconductors. *Nat. Photon.* **16**, 698–706 (2022).
4. Malitson, I. H. Interspecimen Comparison of the Refractive Index of Fused Silica*,†. *J. Opt. Soc. Am., JOSA* **55**, 1205–1209 (1965).
5. Kuzmenko, A. B. Kramers–Kronig constrained variational analysis of optical spectra. *Rev. Sci. Instrum.* **76**, 083108 (2005).
6. Zu, R. *et al.* Optical second harmonic generation in anisotropic multilayers with complete multireflection of linear and nonlinear waves using ♯SHAARP.ml package. *npj Comput Mater* **10**, 1–15 (2024).
7. Liu, F. *et al.* Room-temperature ferroelectricity in CuInP2S6 ultrathin flakes. *Nat. Comm.* **7**, 12357 (2016).
8. Li, D., Hou, X., Kong, F., Wang, K. & Hong, X. Giant Modulation of Second-Harmonic Generation in CuInP2S6 by Interfacing with MoS2 Atomic Layers. *ACS Nano* **18**, 32890–32898 (2024).
9. Neal, S. N. *et al.* Vibrational properties of CuInP2S6 across the ferroelectric transition. *Phys. Rev. B* **105**, 075151 (2022).
10. Yao, J. *et al.* Ferroelectric Schottky diodes of CuInP2S6 nanosheet. *Applied Physics Letters* **123**, 142903 (2023).
11. Yao, X. *et al.* Anomalous polarization enhancement in a van der Waals ferroelectric material under pressure. *Nat. Comm.* **14**, 4301 (2023).
12. Wang, Q. *et al.* Extraordinary tunnel electroresistance in layer-by-layer engineered van der Waals ferroelectric tunnel junctions. *Matter* **5**, 4425–4436 (2022).

13. Ding, W. *et al.* Prediction of intrinsic two-dimensional ferroelectrics in In2Se3 and other III2-VI3 van der Waals materials. *Nat. Comm.* **8**, 14956 (2017).

14. Chiang, Y.-C. *et al.* Spatially Tunable Interfacial Ferroelectricity in Low-Symmetric WTe2. *Nano Lett.* **26**, 864–870 (2026).

15. Kim, B. S. Y. *et al.* Ambipolar charge-transfer graphene plasmonic cavities. *Nat. Mater.* **22**, 838–843 (2023).

16. Neumayer, S. M. *et al.* Giant negative electrostriction and dielectric tunability in a van der Waals layered ferroelectric. *Phys. Rev. Materials* **3**, 024401 (2019).

17. Wang, K. *et al.* Interface-Tuning of Ferroelectricity and Quadruple-Well State in CuInP2S6 via Ferroelectric Oxide. *ACS Nano* **17**, 15787–15795 (2023).

18. Rizzo, D. J. *et al.* Charge-Transfer Plasmon Polaritons at Graphene/α-RuCl3 Interfaces. *Nano Lett.* **20**, 8438 (2020).

19. Jiang, L. *et al.* Soliton-dependent plasmon reflection at bilayer graphene domain walls. *Nat. Mater.* **15**, 840–844 (2016).

20. Luan, Y. *et al.* Imaging Stacking-Dependent Surface Plasmon Polaritons in Trilayer Graphene. *Phys. Rev. Appl.* **18**, 024052 (2022).

21. Das, A. *et al.* Phonon renormalization in doped bilayer graphene. *Phys. Rev. B* **79**, 155417 (2009).
