# Supplementary material for: Ferroelectric-assisted charge-transfer plasmons in graphene/CuInP2S6
Source: Nat Commun. 2026 Aug 17;17:10102. doi: 10.1038/s41467-026-76909-2 (PMC13597499; doi:10.1038/s41467-026-76909-2)
Supplement: Supplementary file 2 [file 41467_2026_76909_MOESM2_ESM.pdf]

nature portfolio

Peer Review File

Ferroelectric-assisted charge-transfer (FACT) plasmons in graphene/CuInP2S6

Corresponding Author: Professor Yinming Shao

Parts of this Peer Review File have been redacted as indicated to remove third-party material.

This file contains all reviewer reports in order by version, followed by all author rebuttals in order by version.

Version 0:

Reviewer comments:

Reviewer #1

(Remarks to the Author)
This manuscript reports high-quality graphene plasmons arising from interfacial charge transfer in a graphene/$CuInP_2S_6$ (CIPS) van der Waals heterostructure. Using nano-infrared imaging, transport, Raman spectroscopy, and PFM measurements, the authors show that the graphene Fermi energy increases with CIPS thickness and saturates together with the ferroelectric response. The authors interpret this correlation as evidence for a ferroelectric-assisted charge-transfer (FACT) plasmon, where bulk ferroelectric polarization modulates interfacial charge transfer. Overall, the work addresses an important question in graphene plasmonics, and the experimental dataset is comprehensive. The concept is helpful for follow-up research on tunable nanophotonics and ferroic–plasmonic coupling. I would recommend publication if the authors could carefully address the following points.

1 The central claim is that ferroelectric polarization assists and controls charge transfer. However, this conclusion currently relies primarily on the correlated thickness dependence of graphene Fermi energy and PFM amplitude. It would be ideal if the authors could provide additional evidence of reversible control, for example by demonstrating modulation of plasmon dispersion or graphene doping upon polarization switching, or by discussing in more detail how bound polarization charges translate into carrier transfer in graphene.

2 Near-field images reveal scattering features attributed to bubbles or local disorder. It would be ideal if the authors could clarify whether plasmon wavelengths are extracted from spatially homogeneous regions and discuss how local inhomogeneity may affect the dispersion analysis and the extracted Fermi energy.

3 The microscopic pathway linking bulk ferroelectric polarization in CIPS to interfacial charge transfer could be articulated more clearly. In particular, it would be helpful to provide a more explicit electrostatic picture describing how polarization-induced bound charges modify the interfacial potential landscape, and how graphene screening and depolarization effects determine the final carrier density. A quantitative comparison between the expected polarization charge density ($\sigma = P$) and the experimentally extracted graphene carrier density would help clarify whether the observed doping level is consistent with simple electrostatic compensation or whether additional interface effects are involved. A brief discussion of how interface-localized electric fields are screened in graphene would further strengthen the mechanistic interpretation. In this regard, recent studies on van der Waals ferroelectric systems (e.g., Nano Lett. 2026, 26, 864) illustrate how interfacial charge redistribution and dipole formation can be treated within a quantitative electrostatic framework.

Minor issues:
1 The manuscript would benefit from a clearer operational definition distinguishing “FACT plasmons” from conventional charge-transfer plasmons.

2 Please clarify measurement conditions (environment, tip bias) for PFM and nano-IR experiments, as these may influence electrostatic contributions.

3 Adding error bars or indicating sample-to-sample variability in the thickness-dependent summary plot would improve statistical robustness.

Reviewer #2

(Remarks to the Author)
This manuscript demonstrates controllable charge-transfer between graphene and CIPS enabled by the vdW interface for engineering multi-level plasmons. The study establishes a direct correlation between the thickness of the CIPS layer, the magnitude of its ferroelectric polarization, and the resulting charge-transfer doping level. The study combines comprehensive experimental characterizations and theoretical calculations, with novel findings and solid experimental support that advance the understanding of ferroelectric-plasmonic coupling in 2D heterostructures. Several key points need clarification before acceptance:
1. Scalability of the FACT plasmon effect. Please clarify the physical behavior and performance variation of the FACT plasmon phenomenon when the device size is scaled down to the nanoscale (e.g., sub-100 nm), including nanoscale ferroelectric domain evolution, interfacial charge effects, and plasmon propagation characteristics.
2. Please clarify why 2-step lithography are needed to etch and deposit the metal electrodes, and explain its advantages over a 1-step lithography method (Ref. 75).
3. Please provide detailed characterization methods and results (Raman, AFM or XTEM) to confirm the monolayer nature of the graphene used in the experiments. In addition, please discuss whether multilayer graphene (bilayer/few-layer) would exhibit the same FACT plasmon behavior and charge-transfer doping characteristics as monolayer graphene.
4. The current thickness-dependent correlation between CIPS polarization and graphene doping is primarily established via material-level PFM characterizations on bare/partial CIPS structures, with limited device-level validation. For practical applications of FACT plasmon-based devices, device-level assessments are essential to evaluate key performance metrics, especially ferroelectric polarization retention.

Version 1:

Reviewer comments:

Reviewer #1

(Remarks to the Author)
The manuscript has been carefully revised, and the authors have addressed my previous concerns. The additional experiments, quantitative analyses, and mechanistic discussions have significantly strengthened the manuscript. I recommend acceptance for publication in Nature Communications.

Reviewer #2

(Remarks to the Author)
This article is comprehensive and informative now. My recommendation is "Accept".

Open Access This Peer Review File is licensed under a Creative Commons Attribution 4.0 International License, which

permits use, sharing, adaptation, distribution and reproduction in any medium or format, as long as you give appropriate credit to the original author(s) and the source, provide a link to the Creative Commons license, and indicate if changes were made.
In cases where reviewers are anonymous, credit should be given to 'Anonymous Referee' and the source.
The images or other third party material in this Peer Review File are included in the article's Creative Commons license, unless indicated otherwise in a credit line to the material. If material is not included in the article's Creative Commons license and your intended use is not permitted by statutory regulation or exceeds the permitted use, you will need to obtain permission directly from the copyright holder.
To view a copy of this license, visit https://creativecommons.org/licenses/by/4.0/

Reviewer #1 (Remarks to the Author):

This manuscript reports high-quality graphene plasmons arising from interfacial charge transfer in a graphene/$CuInP_2S_6$ (CIPS) van der Waals heterostructure. Using nano-infrared imaging, transport, Raman spectroscopy, and PFM measurements, the authors show that the graphene Fermi energy increases with CIPS thickness and saturates together with the ferroelectric response. The authors interpret this correlation as evidence for a ferroelectric-assisted charge-transfer (FACT) plasmon, where bulk ferroelectric polarization modulates interfacial charge transfer. Overall, the work addresses an important question in graphene plasmonics, and the experimental dataset is comprehensive. The concept is helpful for follow-up research on tunable nanophotonics and ferroic–plasmonic coupling. I would recommend publication if the authors could carefully address the following points.

We appreciate the reviewer's careful review and excellent summary of our work.

1 The central claim is that ferroelectric polarization assists and controls charge transfer. However, this conclusion currently relies primarily on the correlated thickness dependence of graphene Fermi energy and PFM amplitude. It would be ideal if the authors could provide additional evidence of reversible control, for example by demonstrating modulation of plasmon dispersion or graphene doping upon polarization switching, or by discussing in more detail how bound polarization charges translate into carrier transfer in graphene.

We thank the Reviewer for this helpful suggestion. To provide additional evidence that CIPS polarization controls graphene doping, we have fabricated additional devices and performed domain-resolved PFM and near-field optical measurements shown in Fig. R1. These new data complement the thickness-dependent E_F and PFM analysis in the original manuscript by comparing different polarization domains within the same hBN/graphene/CIPS heterostructure.

In Fig. R1a, AFM topography identifies the hBN/graphene/CIPS region, with the dashed purple lines marking the graphene-covered area. In Fig. R1b, the PFM phase image shows two main CIPS domains within this graphene-covered region, labeled $P\downarrow$ and $P\uparrow$, corresponding to opposite out-of-plane polarization orientations. In Fig. R1c, the near-field optical phase ϕ_3 measured over the same region shows domain-dependent contrast in the graphene-covered area relative to CIPS. The line profiles in Fig. R1d,e further show that the $P\downarrow$ domain produces a larger near-field phase shift, indicating higher graphene doping, whereas the $P\uparrow$ domain produces a weaker phase shift and lower doping. We note that abundant small polarization bubbles (< 100 nm) are present throughout the sample in Fig. R1b, which makes quantitative extraction of local doping unreliable. Nevertheless, these small PFM features do not by themselves produce resolvable near-field optical contrast in either the bare CIPS area or the $P\uparrow$ graphene-covered region. In contrast, these polarization bubbles scatter plasmons in the $P\downarrow$ graphene-covered region, further supporting the higher doping in this region compared with the $P\uparrow$ region. Since the main $P\downarrow$ and $P\uparrow$ domains are located within the same graphene sheet and on the same CIPS flake, this comparison directly

demonstrates modulation of graphene doping by different CIPS polarization domains. We have added this new data and the relevant discussion in the revised Supplementary Information Sec. VI.

We have also strengthened the mechanistic discussion, as detailed in our response to Question 3 and in the new Supplementary Information Sec. VII. There, we develop an electrostatic model showing how polarization bound charge in CIPS modifies the interfacial potential landscape and drives charge transfer into graphene. We agree that reversible switching of graphene doping or plasmon dispersion would be an ideal further demonstration of *ferroelectric plasmons*. We believe the ferroelectric-assisted charge-transfer (FACT) plasmons and domain-dependent doping modulation demonstrated here will motivate future studies of reversible plasmonic control in graphene/CIPS structures.

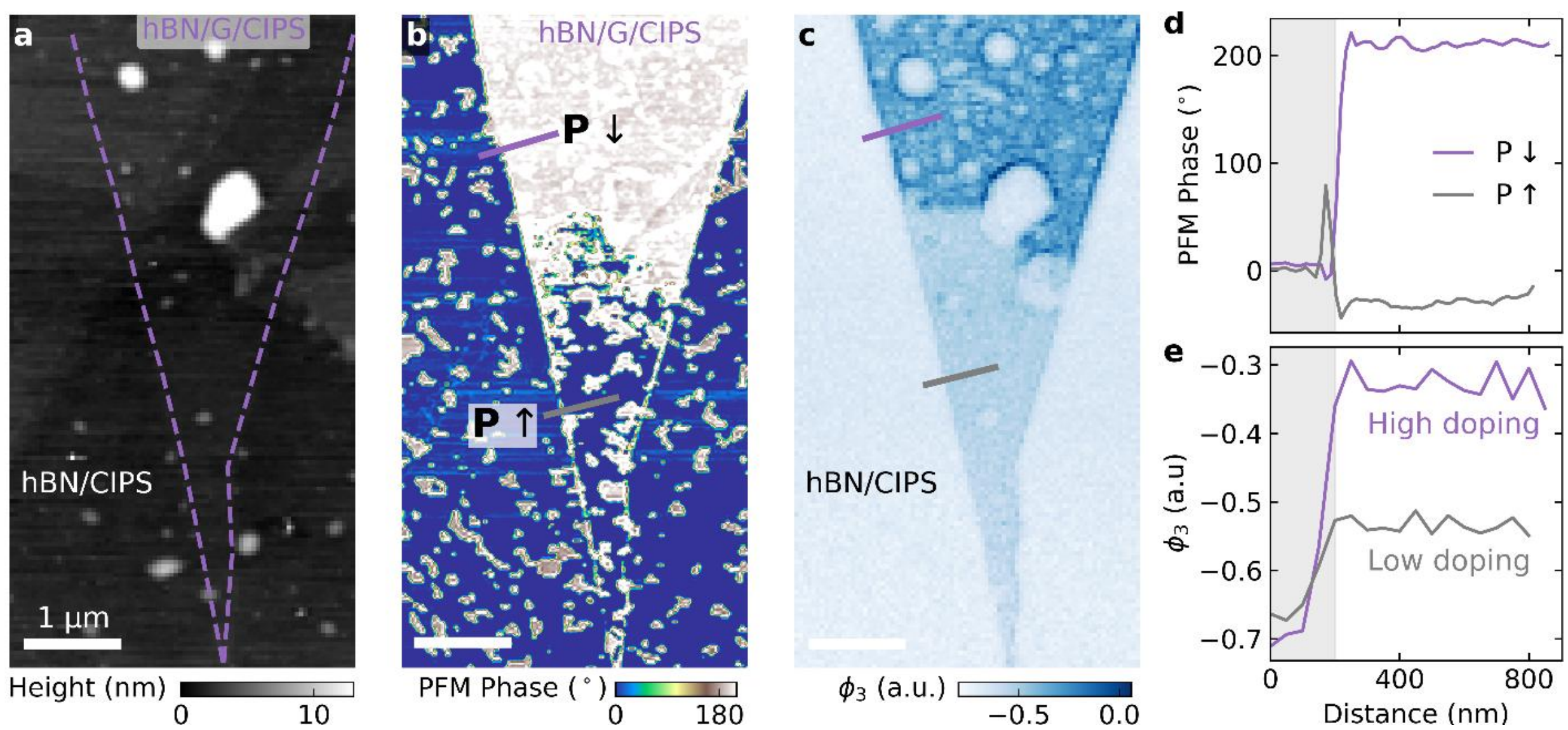

Fig. R1 | Correlation between CIPS polarization domains and graphene doping. a, AFM topography of a hBN/graphene/CIPS heterostructure region. The top hBN covers the entire CIPS region. The dashed purple lines mark the graphene-covered area. **b,** PFM phase image showing two main CIPS domains inside the graphene-covered region with opposite out-of-plane polarizations, labeled $P\downarrow$ and $P\uparrow$. **c,** Near-field optical phase ϕ_3 measured over the same region, showing varying phase contrast across the graphene-covered area relative to the surrounding CIPS. **d,e,** Line profiles taken along the colored lines in **b,c**. The $P\downarrow$ domain shows a larger near-field phase shift, indicating higher graphene doping, whereas the $P\uparrow$ domain shows weaker doping. These spatially correlated PFM and near-field measurements support polarization-dependent charge transfer at the graphene/CIPS interface. Scale bars, 1 μm.

2 Near-field images reveal scattering features attributed to bubbles or local disorder. It would be ideal if the authors could clarify whether plasmon wavelengths are extracted from spatially homogeneous regions and discuss how local inhomogeneity may affect the dispersion analysis and the extracted Fermi energy.

We agree with the reviewer that bubbles and local disorder can affect the plasmonic patterns observed in near-field images. In general, local defects such as bubbles can scatter tip-launched plasmons and produce nearly circular wavefronts, in contrast to the nearly plane-wave-like wavefronts produced by reflection from straight edges. Such radially spreading waves acquire an additional geometrical attenuation factor proportional to $1/\sqrt{r}$, in addition to intrinsic plasmon propagation loss, and therefore decay more rapidly than edge-reflected fringes with nearly planar wavefronts [Dai et al., Nano Lett. **17**, 5285 (2017)]. Similar defect- and edge-reflected plasmon fringes have also been observed in charge-transfer graphene plasmonic structures [Rizzo et al., Nano Lett. **20**, 8438 (2020); Kim et al., Nat. Mater. **22**, 838 (2023)].

In the original manuscript, the plasmon wavelengths used for the primary dispersion analysis were extracted from the relatively homogeneous left-edge region of the 59 nm CIPS device. In the revised manuscript, we have added dashed rectangles in Fig. 1e to indicate the regions used for the line-profile analysis in the original manuscript (see also Fig. R2b). We have also clarified this point in the revised manuscript.

To further assess whether bubble-induced scattering affects the extracted plasmon dispersion and Fermi energy, we performed an additional analysis of tip-launched plasmons reflected by a local bubble, as shown in Fig. R2. The bubble-reflected fringes show a frequency-dependent periodicity similar to that of the edge-reflected plasmons. By comparing the extracted plasmon momenta q_p with calculations of Im r_p, we obtain a graphene Fermi energy of $E_F = 0.60$ eV, in close agreement with $E_F = 0.63$ eV extracted from the edge-reflected plasmon dispersion of the same 59 nm CIPS device in Fig. 2c of the main text. This agreement indicates that local bubble-induced scattering modifies the real-space plasmon wavefront and attenuation, but does not significantly alter the extracted charge-transfer doping level. We have added the relevant discussion to the revised main text and added Fig. R2 in revised Supplementary Information IV.

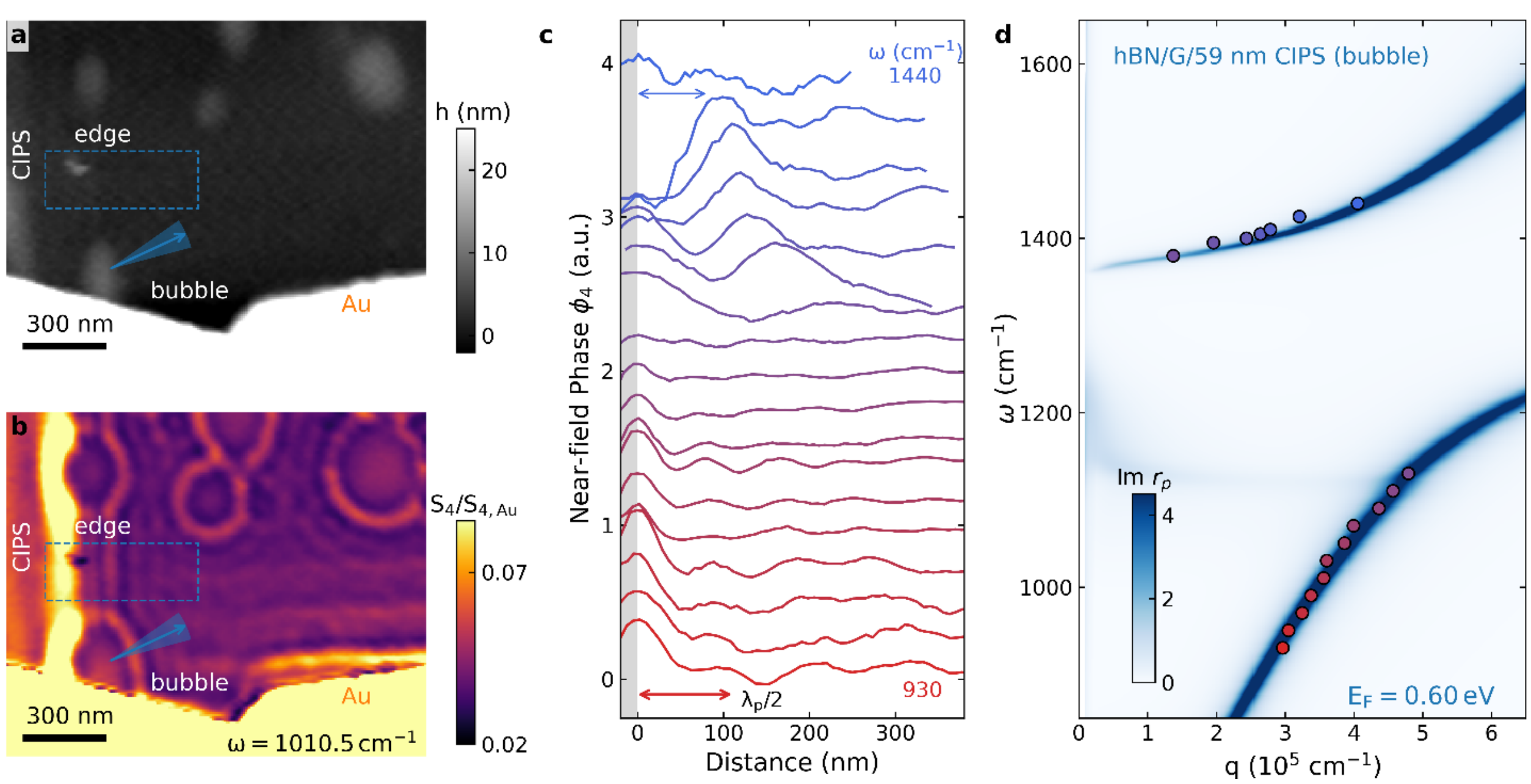

Fig. R2 | Bubble-reflected graphene plasmon dispersion in hBN/graphene/59 nm CIPS. a, AFM topography of the hBN/graphene/59 nm CIPS device near the CIPS edge and Au contact, showing local bubbles that act as nanoscale plasmon reflectors. **b,** Au-normalized near-field amplitude image, $S_4/S_{4,\mathrm{Au}}$, acquired at $\omega = 1010.5\ \mathrm{cm}^{-1}$ in the same region as **a**. Clear plasmon interference fringes are observed around the bubble and device edges. **c,** Near-field phase line profiles, ϕ_4, taken from the bubble region over laser frequencies from 930 to 1440 cm^{-1}. The fringe periodicity changes with increasing frequency and corresponds to $\lambda_p/2$, consistent with tip-launched plasmons reflected by the bubble. **d,** Extracted plasmon momenta q_p overlaid on the calculated imaginary part of the *p*-polarized reflection coefficient, $\mathrm{Im}\, r_p$, for the hBN/graphene/CIPS heterostructure. The dispersion is well described by a graphene Fermi energy of $E_F = 0.60\ \mathrm{eV}$, close to the value extracted from the edge-reflected plasmon (blue dashed rectangles) dispersion of the same device in Fig. 2c of the main text, $E_F = 0.63$ eV.

3 The microscopic pathway linking bulk ferroelectric polarization in CIPS to interfacial charge transfer could be articulated more clearly. In particular, it would be helpful to provide a more explicit electrostatic picture describing how polarization-induced bound charges modify the interfacial potential landscape, and how graphene screening and depolarization effects determine the final carrier density. A quantitative comparison between the expected polarization charge density (σ = P) and the experimentally extracted graphene carrier density would help clarify whether the observed doping level is consistent with simple electrostatic compensation or whether additional interface effects are involved. A brief discussion of how interface-localized electric fields are screened in graphene would further strengthen the mechanistic interpretation. In this regard, recent studies on van der Waals ferroelectric systems (e.g., Nano Lett. 2026, 26, 864) illustrate how interfacial charge redistribution and dipole formation can be treated within a quantitative electrostatic framework.

We thank the Reviewer for this constructive comment, which prompted us to formulate the mechanism as an explicit electrostatic model. The full model and numerical details are given in a new Supplementary Information Sec. VII; here we summarize the picture and the requested comparisons (see also new Fig. R3 below).

Recent vdW ferroelectric studies, including the WTe_2 work (*Nano Lett. 2026, 26, 864*) mentioned by the Reviewer, show that interfacial charge redistribution and dipole formation can be treated quantitatively from first principles. In our system, DFT calculations explicitly capture the atomistic graphene/CIPS interface, including the relaxed graphene/CIPS separation and charge transfer in ultrathin CIPS (Figs. 1b and 1c and Extended Data Fig. 3 in the original main text). The resulting $|E_F| \approx 0.2$ eV for graphene on bilayer CIPS provides a microscopic reference for the baseline interfacial charge transfer. For thicker CIPS (~5 – 60 nm), we extend this picture using an electrostatic model in which the CIPS depolarization field is partially screened by graphene and provides the ferroelectric-induced contribution to charge transfer.

The microscopic pathway is as follows. The thickness-dependent out-of-plane polarization of CIPS, $P_{\mathrm{eff}}(d)$, produces bound surface charge at the CIPS surfaces:

$$\sigma_b(d) = \hat{n} \cdot \mathbf{P}_{\mathrm{eff}}(d) \simeq \pm P_{\mathrm{eff}}(d). \qquad Eq. 1$$

This bound charge modifies the graphene/CIPS interfacial potential and induces an image charge in graphene. Because graphene has a finite density of states and is separated from CIPS by a van der Waals gap, this induced image charge does not fully compensate the bound polarization charge. Instead, the induced charge is set self-consistently by graphene band filling, the graphene/CIPS gap capacitance, and the residual depolarization field in CIPS.

The charge-transfer process in graphene/CIPS can be viewed as a ferroelectric extension of the work-function-mediated charge-transfer model [Kim et al., Nat. Mater. 22, 838 (2023)]. In the model of Kim et al., the graphene density is set by the work-function mismatch, graphene band filling, and the Poisson voltage drop across an insulating spacer of thickness t. Written in terms of an areal spacer capacitance, $C_{\mathrm{gap}} = \varepsilon_0 \varepsilon / t$, the conventional charge-transfer balance is:

$$\hbar v_F \sqrt{\pi n} + \frac{en}{C_{\mathrm{gap}}} = \Delta\Phi_{\mathrm{WF}}, \qquad Eq. 2$$

where n is the graphene carrier density, $E_F = \hbar v_F \sqrt{\pi n}$, and $\Delta\Phi_{\mathrm{WF}} = \Phi - \Phi_G$ is the work function mismatch between graphene (Φ_G) and the active layer (Φ). In graphene/CIPS, the residual uncompensated polarization charge gives rise to a depolarization field in CIPS, which contributes to the interfacial electrostatic potential and modifies the charge-transfer balance. We write the total graphene carrier density as:

$$n(d) = n_0 + n_{\mathrm{FE}}(d), \qquad Eq. 3$$

where n_0 is the baseline work-function charge-transfer density obtained in the ultrathin CIPS limit, and $n_{\mathrm{FE}} = n - n_0$ is the ferroelectric-induced contribution. The residual uncompensated polarization charge, $P_{\mathrm{eff}}(d) - e[n(d) - n_0]$, produces an average depolarization field in CIPS:

$$E_{\mathrm{dep}}(d) = \frac{P_{\mathrm{eff}}(d) - e(n - n_0)}{\varepsilon_0 \varepsilon_b}, \qquad Eq. 4$$

where ε_b is the static dielectric constant of CIPS. The depolarization contribution to the interfacial potential can be written using the CIPS depolarization capacitance $C_{\mathrm{dep}}(d) = \varepsilon_0 \varepsilon_b / d$, where we used $\varepsilon_b = 7$ (Phys. Rev. Mater. 3, 024401 (2019)). The charge-transfer balance is then:

$$\hbar v_F \sqrt{\pi n} + \frac{en}{C_{\mathrm{gap}}} = \Delta\Phi_{\mathrm{WF}} + \frac{P_{\mathrm{eff}}(d) - e(n - n_0)}{C_{\mathrm{dep}}(d)}. \qquad Eq. 5$$

This equation shows that graphene doping and the CIPS depolarization field can be determined self-consistently. The bound polarization charge drives charge transfer into graphene, while the induced graphene charge screens the polarization charge and reduces the residual depolarization

field. As $P_{\text{eff}}(d)$ is strongly suppressed in ultrathin CIPS and recovers with increasing thickness, the calculated rise of $|E_F|$ mainly reflects the recovery of $P_{\text{eff}}(d)$. The experimental PFM amplitude is used as an indicator of the thickness-dependent out-of-plane ferroelectric response. As shown in Fig. R3a, we fit the rising part of the experimental PFM amplitude (below ~100 nm) with a phenomenological model $A_{\text{PFM}}(d) = A_0 + A_1 f(d)$, where $f(d) = ((d - d_c)/d)^{\beta}$ and d_c is a critical thickness so that $f(d < d_c) = 0$. Using the PFM-derived $P_{\text{eff}}(d)$ as the input (green curve in Fig. R3a), the model in Eq. 5 reproduces the observed increase of graphene $|E_F|$ in the single-domain thickness range, as shown in Fig. R3b. The explicit electrostatic model and quantitative comparison support that the observed FACT plasmons are consistent with the electrostatic compensation picture. We have added the relevant discussion in the revised main text and Fig. R3 in the revised Supplementary Information Sec. VII. We have also added *Nano Lett. 2026, 26, 864* as Ref. 51 in the revised manuscript.

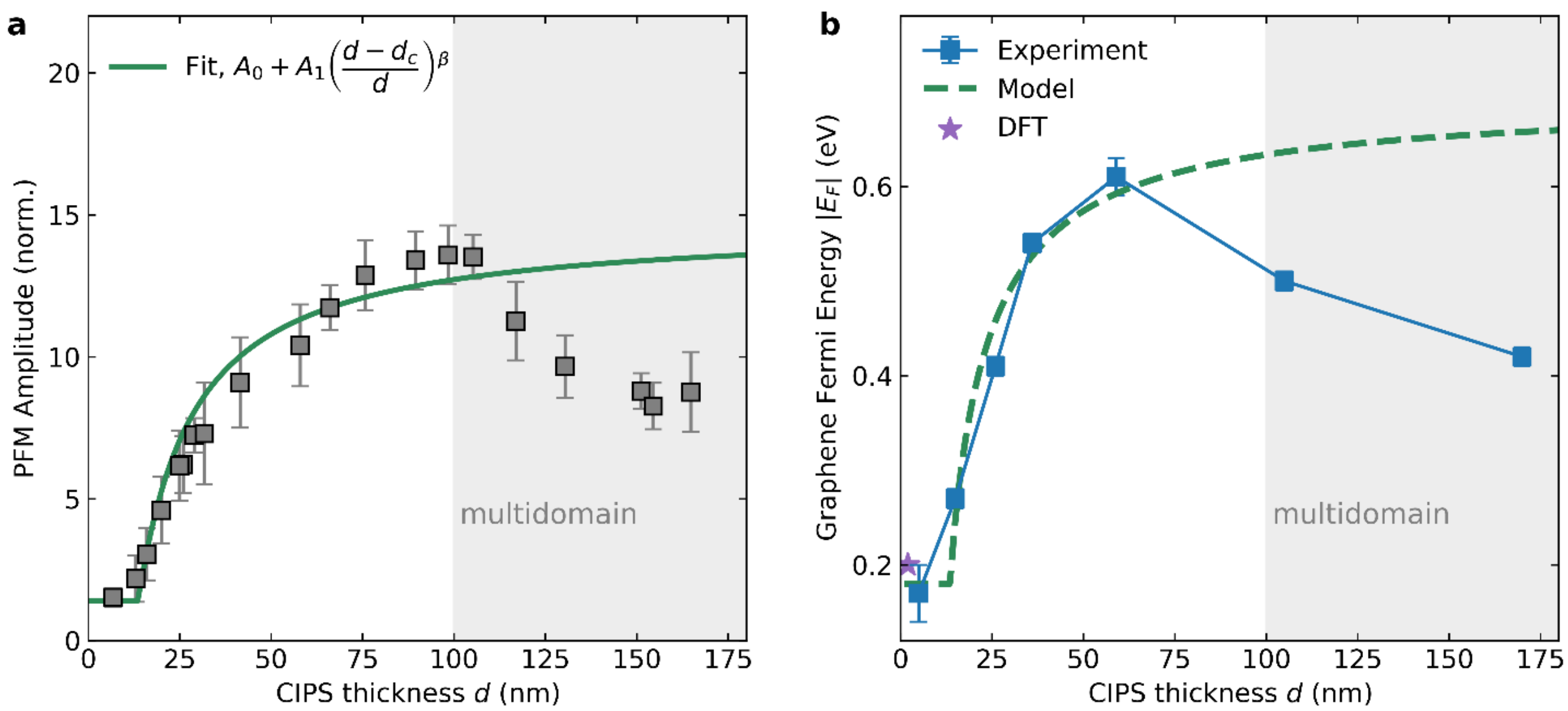

Fig. R3 | Thickness-dependent PFM response and electrostatic charge-transfer model. a, Normalized PFM amplitude as a function of CIPS thickness. The green curve is a fit to the rising PFM branch below 100 nm using $A_{\text{PFM}}(d) = A_0 + A_1 f(d)$, where $f(d) = 0$ for $d \leq d_c$ and $f(d) = ((d - d_c)/d)^{\beta}$ for $d > d_c$. The fit gives $d_c = 13.5$ nm and $\beta = 1.1$. The gray shaded region marks the thickness range where multidomain or mixed-polarization textures become important, and the single-domain fit is no longer valid. **b,** Graphene Fermi energy (blue squares) extracted from plasmon dispersion as a function of CIPS thickness. The green dashed curve is the electrostatic charge-transfer model using $P_{\text{eff}}(d) = P_{\text{bulk}}^{\text{model}} f(d)$ as the thickness-dependent polarization input. The purple star shows the DFT-calculated Fermi energy for graphene on bilayer CIPS. The model captures the rise of $|E_F|$ in the single-domain thickness range (<100 nm), while the reduced $|E_F|$ in thicker CIPS is consistent with domain averaging that lowers the net interfacial polarization charge sensed by graphene.

Minor issues:

1 The manuscript would benefit from a clearer operational definition distinguishing "FACT plasmons" from conventional charge-transfer plasmons.

We thank the reviewer for this constructive suggestion. Conventional charge-transfer plasmons in graphene are determined by the work function mismatch at the interface $\Delta\Phi$. In the original manuscript, we have demonstrated experimentally that in graphene/CIPS heterostructures the charge transfer is determined not by the interface alone, but also by the thickness of the neighboring CIPS. As detailed in our response to Question 3 and in the new Supplementary Information Sec. VII, polarization bound charge and the associated depolarization field in CIPS enter the electrostatic balance that determines the graphene carrier density.

We therefore define FACT plasmons as charge-transfer graphene plasmons whose carrier density, and hence plasmon dispersion, is controlled by ferroelectric polarization in addition to interfacial work-function mismatch (Eq. 5). We have added this definition of FACT plasmons in the revised manuscript. This definition is now supported by the thickness-dependent E_F, the full electrostatic model, and the domain-resolved PFM/near-field measurements showing polarization-dependent graphene doping.

2 Please clarify measurement conditions (environment, tip bias) for PFM and nano-IR experiments, as these may influence electrostatic contributions.

We thank the reviewer for this helpful suggestion. We have added additional measurement conditions including the environment and tip bias for the PFM and nano-IR experiments in the Methods section of the revised manuscript.

3 Adding error bars or indicating sample-to-sample variability in the thickness-dependent summary plot would improve statistical robustness.

We thank the reviewer for this important suggestion. We have added error bars in the thickness-dependent summary plot (Fig. 4e) in the revised manuscript. The error bars are determined by extraction of the plasmon wavelength from different regions of the sample and/or from comparison with calculations.

Reviewer #2 (Remarks to the Author):

This manuscript demonstrates controllable charge-transfer between graphene and CIPS enabled by the vdW interface for engineering multi-level plasmons. The study establishes a direct correlation between the thickness of the CIPS layer, the magnitude of its ferroelectric polarization, and the resulting charge-transfer doping level. The study combines comprehensive experimental characterizations and theoretical calculations, with novel findings and solid experimental support that advance the understanding of ferroelectric-plasmonic coupling in 2D heterostructures. Several key points need clarification before acceptance:

We appreciate the reviewer's careful review and constructive comments on our work.

1. Scalability of the FACT plasmon effect. Please clarify the physical behavior and performance variation of the FACT plasmon phenomenon when the device size is scaled down to the nanoscale (e.g., sub-100 nm), including nanoscale ferroelectric domain evolution, interfacial charge effects, and plasmon propagation characteristics.

We thank the reviewer for this constructive comment. The thickness dependence of FACT plasmons, including CIPS flakes below 100 nm thick, has been discussed in Fig. 4 of the original manuscript. We therefore clarify here the lateral nanoscale response of FACT plasmons near sub-100-nm structural features. As shown in Fig. R4, FACT plasmons remain visible near several local nanoscale structures: a bubble with a lateral size ~100 nm, an atomically sharp bilayer graphene domain wall, and a sharp graphene/CIPS edge. These nanoscale structures mainly modify the propagation characteristics of FACT plasmons. For example, FACT plasmons can be scattered into ring-like fringes around a bubble (Fig. R4b), reflected by a line-like domain wall (Fig. R4d), or confined and guided along the graphene edge (Fig. R4f). Thus, nanoscale structures can reshape the plasmonic propagation pattern, but they do not eliminate the FACT plasmon response.

This behavior is consistent with the mechanism of FACT plasmons, as detailed in our response to Reviewer 1, Question 3. The interfacial charge transfer is primarily determined by the local graphene/CIPS interface, CIPS polarization, and graphene screening. Reducing the lateral device size does not eliminate the charge-transfer mechanism, provided that the CIPS remains polarized and the interface remains intact. We have added this discussion to the revised Supplementary Information Sec. VIII.

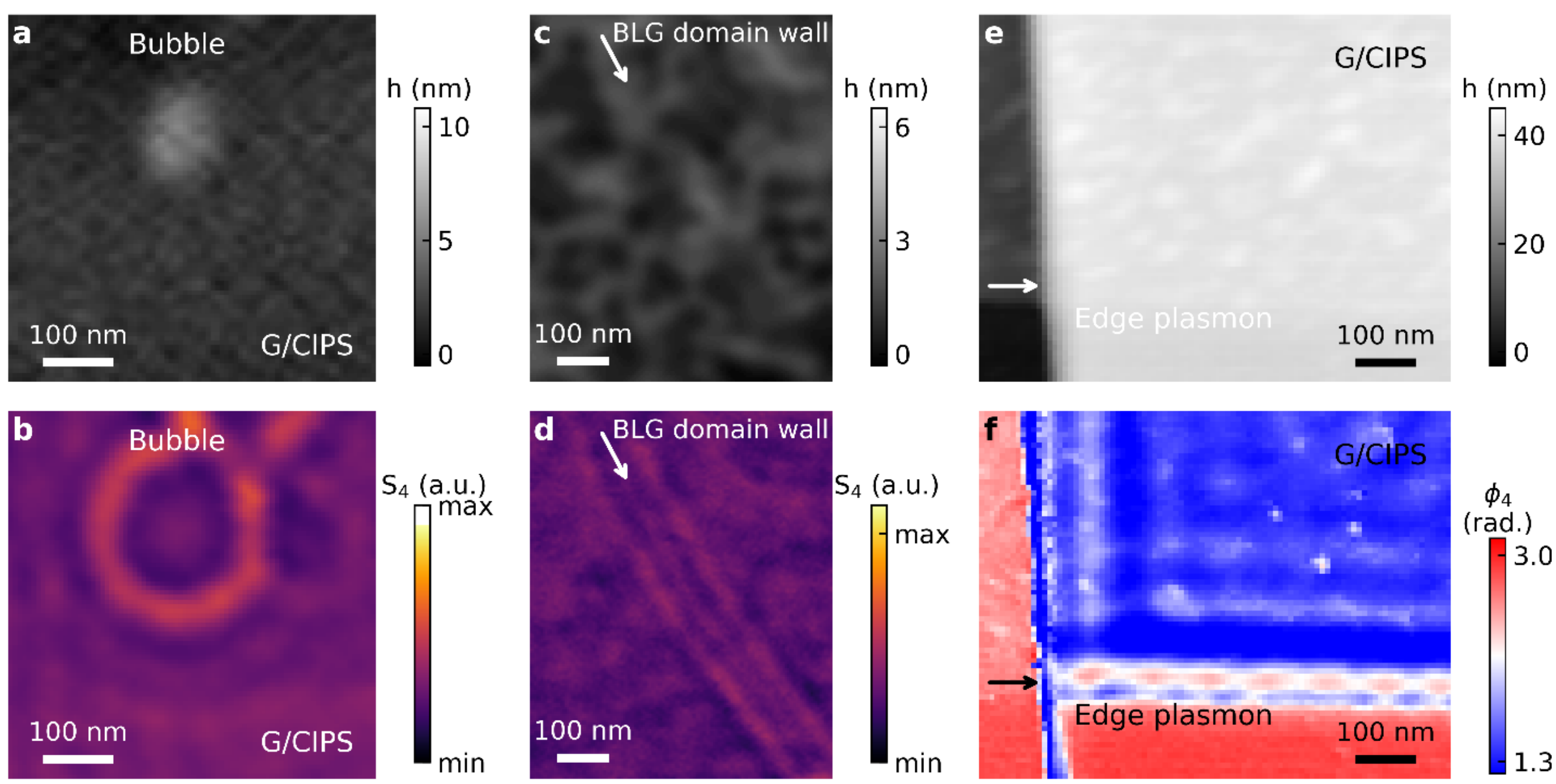

Fig. R4 | FACT plasmon response near local nanoscale structures. a,b, AFM topography and near-field amplitude S_4 around a nanoscale bubble in graphene/CIPS. The bubble, ~100 nm in lateral size, locally perturbs the charge transfer at the interface and scatters FACT plasmons,

producing a ring-like near-field fringe pattern. **c,d,** Topography and near-field amplitude S_4 image near a bilayer graphene (BLG) domain wall. The atomic-scale BLG domain wall is not resolved in the AFM topography (white arrow), but it acts as a sharp line scatterer that reflects FACT plasmons. **e,f,** Topography and near-field phase ϕ_4 near a sharp graphene/CIPS edge. In addition to plasmons propagating in the interior of graphene, the sharp boundary between G/CIPS and bare CIPS also supports edge-confined plasmons that propagate along the graphene edge (black arrow).

2. Please clarify why 2-step lithography are needed to etch and deposit the metal electrodes, and explain its advantages over a 1-step lithography method (Ref. 75).

We thank the reviewer for this helpful comment. We have clarified the reason for using two-step lithography in the revised Supplementary Information Sec. III.

In our (top) hBN/G/CIPS (bottom) device, graphene is fully covered by a thin top hBN and placed on CIPS. Therefore, direct metal deposition cannot contact graphene unless the top hBN is first opened. The first lithography step defines small contact windows, followed by reactive ion etching to locally expose the graphene contact region (Fig. S4). The second lithography step defines the larger metal leads, followed by Ti/Au deposition. In this geometry, metal contacts graphene only at the pre-defined etched windows, while the extended leads remain on top of insulating hBN.

This separation between “contact-window definition” and “lead definition” is important for controlling the effective device geometry. If etching and metal deposition were performed using the same one-step lithography pattern, the metal would be deposited over a larger etched/exposed graphene region. This could make the channel length less well defined, and could increase the risk of unintended leakage or electrical shorting. The two-step process therefore provides better control of the graphene contact area, deterministic channel geometry, and more reliable device fabrication.

This approach is commonly used in hBN-encapsulated graphene and other 2D materials devices, where an etch step is required to expose the buried 2D channel before metal deposition, and where precise definition of contact area is necessary (see *Nat. Commun.* 9, 5387 (2018), *ACS Nano* 18, 17111 (2024)).

Our approach follows the same general principle as Ref. 75 in the original manuscript (Ref. 77 in the revised manuscript), where the graphene edge is exposed by etching and then contacted by metal deposition. The key difference is the device geometry. In Ref. 75, the encapsulated hBN/graphene/hBN stack can be etched into a defined device shape, such as a van der Pauw or rectangular channel, before metal deposition. In our device, however, graphene is placed on top of CIPS and covered by the top hBN, forming a hBN/G/CIPS heterostructure. Because CIPS is not etched together with the graphene/hBN stack, we cannot define the full device shape by etching the entire heterostructure as in Ref. 75. Instead, we use the first lithography step only to open localized contact windows through the top hBN and expose the graphene edge/contact region. The second lithography step then defines the extended metal leads. Thus, our process preserves the CIPS/graphene interface while still achieving edge contacts to graphene. The advantage of this

two-step process in our device is therefore not that it replaces the method in Ref. 75, but that it adapts the established edge-contact concept to a hBN/G/CIPS geometry where the bottom CIPS layer must remain intact.

3. Please provide detailed characterization methods and results (Raman, AFM or XTEM) to confirm the monolayer nature of the graphene used in the experiments. In addition, please discuss whether multilayer graphene (bilayer/few-layer) would exhibit the same FACT plasmon behavior and chargetransfer doping characteristics as monolayer graphene.

We thank the reviewer for this important question on the impact of the number of graphene layers on the observed FACT plasmons. First, we confirm that monolayer graphene was used in the main text results through Raman spectroscopy and atomic force microscopy (AFM) measurements.

As shown in Fig. R5a, we performed AFM topography measurements near the folded-hBN boundary (Fig. 2d in the main text), where adjacent hBN/G/CIPS (5 nm) and hBN/CIPS (5 nm) regions can be identified. The height profile taken across the graphene edge, marked by the dashed orange line, shows a step height of approximately 0.4 nm (Fig. R5b), consistent with the nominal thickness of monolayer graphene (~0.33 nm). Near-field imaging of the same region further confirms the presence of graphene: the near-field phase image exhibits a clear contrast only in the hBN/G/CIPS (5 nm) region, while the neighboring hBN/CIPS region shows no comparable graphene-related response (Fig. R5c). In addition, Raman spectra taken from the same graphene flake on SiO_2 (gray dots in Fig. 2f and 2g of the main text) show a G peak at 1584.5 cm^{-1} and a 2D peak at 2680.9 cm^{-1}, with an intensity ratio I_{2D}/I_G = 5.97 (Fig. R5d). The large 2D-to-G intensity ratio and the phonon positions are characteristic of high-quality undoped monolayer graphene, further confirming the monolayer nature of the graphene used in the hBN/G/CIPS (5 nm) device. We have added Fig. R5 and the relevant discussion to the revised Supplementary Information Sec. IX to document this characterization.

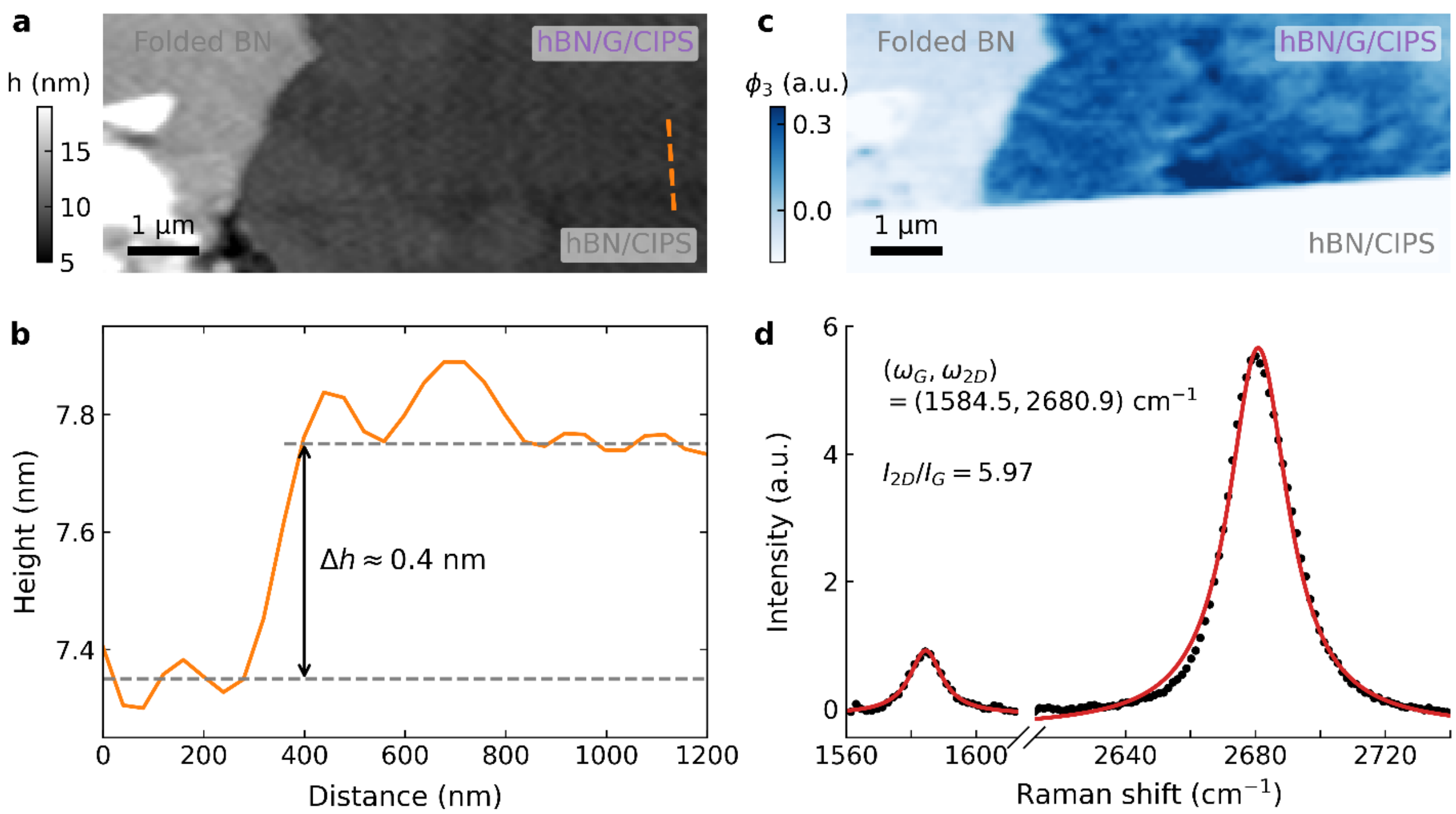

Fig. R5 | Verification of monolayer graphene in the hBN/G/CIPS (5 nm) sample in main text Fig. 2. **a**, AFM topography of the folded-hBN boundary and adjacent hBN/G/CIPS and hBN/CIPS regions. The dashed orange line marks the linecut location across the graphene edge. **b**, AFM height profile across the dashed line in **a**, showing a step height of approximately 0.4 nm, consistent with monolayer graphene. **c**, Near-field phase image (ϕ_3) of the same region in **a**, showing clear contrast when graphene is present. **d**, Raman spectrum from the same graphene flake (gray dot in Fig. 2f). The fitted G and 2D peak positions are consistent with undoped graphene, with intensity ratio $I_{2D}/I_G = 5.97$, further confirming the monolayer nature of graphene (Phys. Rev. B 79, 155417 (2009)).

As suggested by the Reviewer, we carried out new near-field optical measurements of bilayer graphene on CIPS and observed prominent plasmonic features. Prior near-field studies (Fig. R6a) showed that in bilayer graphene, tensile and shear domain-wall solitons produce distinct plasmon reflection profiles, with characteristic fringe spacings of $\lambda_p/2$ and $3\lambda_p/4$, respectively (Nat. Mater. **15**, 840–844, 2016).

In our hBN/bilayer graphene/CIPS heterostructure, the near-field amplitude measured at 900 cm^{-1} shows clear plasmonic fringes near both the graphene edge and internal line-like features assigned to bilayer graphene domain walls (Fig. R6b). The edge profile shows a fringe spacing of approximately $\lambda_p/2$, consistent with conventional edge-reflected graphene plasmons. In contrast, the two domain-wall profiles show different interference patterns: DW 1 exhibits peak spacing consistent with $3\lambda_p/4$, while DW 2 shows a spacing close to $\lambda_p/2$ (Fig. R6c). These profiles are consistent with the established near-field signatures of different bilayer graphene domain-wall solitons.

To further verify the plasmonic origin of the domain-wall contrast, we measured frequency-dependent line profiles across DW 1 from 860 to 1050 cm^{-1}. The interference peaks shift systematically with infrared frequency, as expected for dispersive plasmon polaritons rather than static topographic or electrostatic contrast (Fig. R6d). The extracted plasmon momenta agree well with the calculated hBN/bilayer graphene/CIPS plasmon dispersion using a Fermi energy of E_F= 0.3 eV (Fig. R6e). This agreement indicates that charge transfer from CIPS also dopes bilayer graphene sufficiently to support propagating plasmons without a gate voltage.

[Figure Redacted]

Fig. R6 | Domain-wall plasmons in bilayer graphene on CIPS. **a**, Reference near-field line profiles from bilayer graphene plasmons near the edge and domain walls under a gate voltage of -80 V (from Nat. Mater. **15**, 840 (2016), Ref. 11 in the revised Supplementary Information). Different types of domain walls (tensile vs shear) in bilayer graphene are characterized by different fringe spacings ($\lambda_p/2$ vs $3\lambda_p/4$). **b**, Near-field amplitude image $S_4$of the hBN/bilayer graphene (BLG)/CIPS heterostructure at 900 cm^{-1}. Colored arrows mark representative linecuts across the edge and domain-wall regions. **c**, Line profiles extracted from panel **b**. The edge profile shows a fringe spacing of approximately $\lambda_p/2$, while domain-wall profiles DW 1 and DW 2 show spacing consistent with $3\lambda_p/4$ and $\lambda_p/2$, respectively. **d**, Near-field line profiles across DW 1 measured at different infrared frequencies, with extracted peak positions indicated by colored dots. **e**, Plasmon dispersion extracted from the DW 1 fringe spacing, compared with the calculated hBN/BLG/CIPS plasmon dispersion for $E_F = 0.3$ eV.

The plasmonic response of few-layer graphene beyond bilayer graphene remains less explored than that of monolayer graphene. Recent near-field infrared measurements on trilayer graphene showed that Bernal (ABA) and rhombohedral (ABC) stacking support distinct plasmonic responses, with different plasmon wavelengths and strong plasmon reflection at ABA/ABC lateral junctions (Phys. Rev. Appl. 18, 024052 (2022)). These results indicate that, in few-layer graphene, plasmon propagation is governed not only by carrier density but also by domain walls, stacking-dependent band structure, and optical conductivity.

In summary, we have demonstrated that FACT plasmons exist in bilayer graphene on CIPS and should persist in thicker few-layer graphene, but their doping response and plasmon dispersion are complicated by stacking-dependent band structures. Given the rich correlated physics recently discovered in rhombohedral few-layer graphene, we believe our observation of high-quality charge-transfer plasmons in graphene/CIPS heterostructures will motivate future studies of FACT plasmons in trilayer and thicker few-layer graphene on vdW ferroelectric substrates. We have added this discussion in the revised Supplementary Information Sec. VIII.

4. The current thickness-dependent correlation between CIPS polarization and graphene doping is primarily established via material-level PFM characterizations on bare/partial CIPS structures, with limited device-level validation. For practical applications of FACT plasmon-based devices, device-level assessments are essential to evaluate key performance metrics, especially ferroelectric polarization retention.

We thank the Reviewer for this important comment. We agree that device-level validation and polarization retention are important metrics for future FACT plasmonic devices. In the present work, the device-level response is directly measured by transport and nano-IR measurements on hBN/graphene/CIPS heterostructure devices, such as those shown in Figs. 1d and 1e of the original manuscript. From these measurements, the graphene plasmon wavelength and doping level are extracted directly from the actual plasmonic devices. The PFM measurements on bare CIPS are used to establish the thickness-dependent ferroelectric response of CIPS, because quantitative PFM through the hBN/doped-graphene stack can be complicated by electrostatic screening and redistribution of the tip electric field.

The combined measurements therefore establish the key device-relevant correlation: CIPS thickness controls the out-of-plane ferroelectric response, which in turn controls graphene doping and FACT plasmon wavelength in graphene/CIPS heterostructures. The observation of robust plasmon propagation in completed hBN/graphene/CIPS devices indicates that the FACT plasmon mechanism is retained after multiple device fabrication steps, as also discussed in our response to Question 2. Since near-field optical contrast and plasmon wavelength are established indicators of graphene doping, repeated nano-IR measurements provide a device-level probe of the stability of the FACT plasmons. To further address the Reviewer's comment, we performed additional measurements on the same hBN/graphene/CIPS device region after ~6 months. As shown in the new Fig. R7, both the overall graphene phase contrast and the fringe spacing extracted from edge-

reflected FACT plasmons remain stable within experimental uncertainty. These results provide device-level evidence that the FACT plasmon response and the associated charge-transfer doping are retained over extended time. We have added Fig. R7 and relevant discussion in the revised Supplementary Information Sec. VIII.

We agree that more systematic retention and endurance measurements, such as repeated polarization writing/erasing, are important for practical applications. We have added this discussion to the revised manuscript as an outlook for future FACT plasmonic device studies.

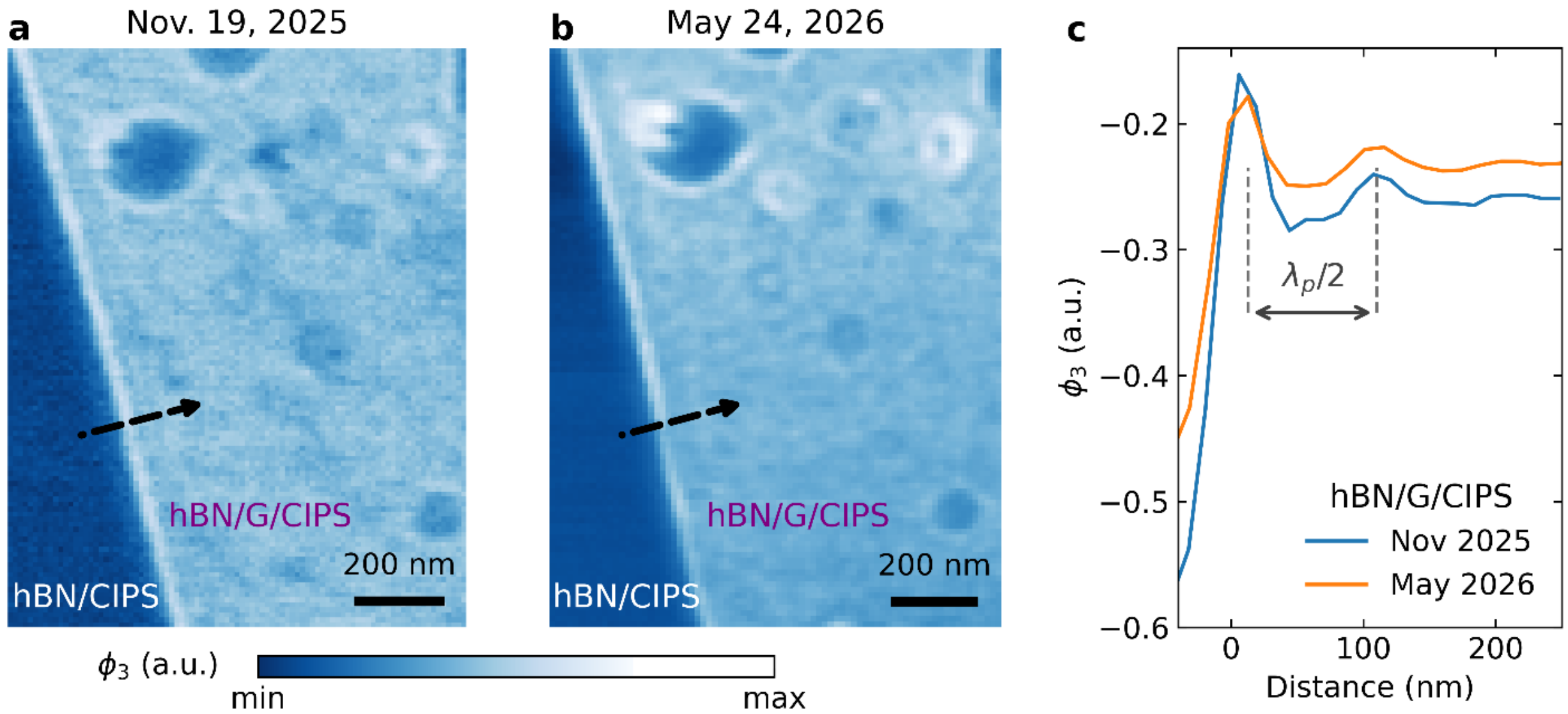

Fig. R7 | Long-term stability of the FACT plasmon response. a,b, Near-field phase ϕ_3 images of the same hBN/graphene/CIPS device region measured on Nov. 19, 2025 and May 24, 2026, respectively. The graphene/CIPS edge and the edge-reflected plasmon fringe remain clearly visible after ~6 months. Dashed arrows indicate the positions used to extract the line profiles. **c,** Line profiles of ϕ_3 across the graphene/CIPS edge for the two measurements. Similar fringe spacing indicates that the plasmon wavelength (λ_p), and therefore the charge-transfer-induced graphene doping level, remains stable over this time period. These results provide device-level evidence that the FACT plasmon response is retained in completed hBN/graphene/CIPS heterostructures over extended time.

Reviewer #1 (Remarks to the Author):

The manuscript has been carefully revised, and the authors have addressed my previous concerns. The additional experiments, quantitative analyses, and mechanistic discussions have significantly strengthened the manuscript. I recommend acceptance for publication in Nature Communications.

We thank the Reviewer for carefully re-evaluating our revised manuscript and for recommending acceptance. We appreciate the Reviewer's careful evaluation throughout the review process.

Reviewer #2 (Remarks to the Author):

This article is comprehensive and informative now. My recommendation is "Accept".

We thank the Reviewer for the positive assessment of our revised manuscript and for recommending acceptance. We appreciate the Reviewer's careful evaluation throughout the review process.
